# Poly(vinyl alcohol) Molecular Bottlebrushes Nucleate Ice

**DOI:** 10.1021/acs.biomac.2c01097

**Published:** 2022-11-28

**Authors:** Panagiotis G. Georgiou, Nina L. H. Kinney, Ioanna Kontopoulou, Alexander N. Baker, Steven A. Hindmarsh, Akalabya Bissoyi, Thomas R. Congdon, Thomas F. Whale, Matthew I. Gibson

**Affiliations:** † Department of Chemistry, University of Warwick, Gibbet Hill Road, CV4 7AL Coventry, U.K.; ‡ Division of Biomedical Sciences, Warwick Medical School, University of Warwick, Gibbet Hill Road, CV4 7AL Coventry, U.K.; § Department of Physics, University of Warwick, Gibbet Hill Road, CV4 7AL Coventry, U.K.

## Abstract

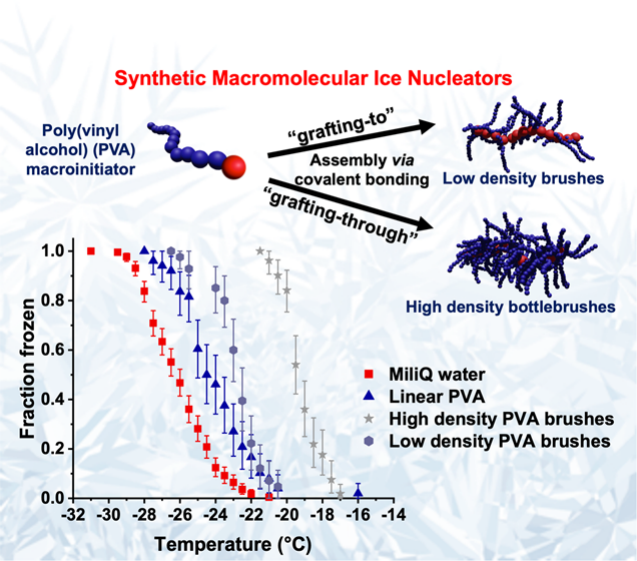

Ice binding proteins (IBP) have evolved to limit the growth of ice but also to promote ice formation by ice-nucleating proteins (INPs). IBPs, which modulate these seemingly distinct processes, often have high sequence similarities, and molecular size/assembly is hypothesized to be a crucial determinant. There are only a few synthetic materials that reproduce INP function, and rational design of ice nucleators has not been achieved due to outstanding questions about the mechanisms of ice binding. Poly(vinyl alcohol) (PVA) is a water-soluble synthetic polymer well known to effectively block ice recrystallization, by binding to ice. Here, we report the synthesis of a polymeric ice nucleator, which mimics the dense assembly of IBPs, using confined ice-binding polymers in a high-molar-mass molecular bottlebrush. Poly(vinyl alcohol)-based molecular bottlebrushes with different side-chain densities were synthesized *via* a combination of ring-opening metathesis polymerization (ROMP) and reversible addition–fragmentation chain-transfer (RAFT) polymerization, using “grafting-to” and “grafting-through” approaches. The facile preparation of the PVA bottlebrushes was performed *via* selective hydrolysis of the acetate of the poly(vinyl acetate) (PVAc) side chains of the PVAc bottlebrush precursors. Ice-binding polymer side-chain density was shown to be crucial for nucleation activity, with less dense brushes resulting in colder nucleation than denser brushes. This bio-inspired approach provides a synthetic framework for probing heterogeneous ice nucleation and a route toward defined synthetic nucleators for biotechnological applications.

## Introduction

Ice-binding proteins recognize and bind ice faces, leading to control over their formation (ice-nucleating proteins, INPs) or growth (antifreeze proteins, AFPs). AFPs are well studied and have defined ice-binding sites to engage ice either directly or *via* an anchored mechanism.^1,2^ INPs, in contrast, are less well understood^3^ despite being commercialized as extracts from the bacterium *Pseudomonas syringae*. IBPs have many potential applications across a range of biotechnological and engineering areas where it would be advantageous to control ice formation.^4^ The study of INPs is challenging due to the low solubility of the INP and the need for the transmembrane/lipid anchored domains for activity. Davies and co-workers reported recombinant mutated INPs, but it was essential to study activity while attached to the host organism (*E.coli*) to obtain ice nucleation functional data.^5^

In addition to IBPs, it has emerged that synthetic materials can also bind ice, reproducing the macroscopic properties of IBPs—presenting opportunities not only for biomedical applications but also to understand IBP function.^6−8^ Synthetic polymers can be produced with excellent control over architecture and composition, allowing molecular shape and composition to be tuned to probe the material/ice interface. The most active and widely studied ice-binding polymer is poly(vinyl alcohol) (PVA).^9,10^ PVA’s ice recrystallization inhibition (IRI) activity can be tuned by varying molecular weight,^11^ shape,^12,13^ and assembly into particles.^14^ PVA binds to ice *via* hydrogen bonding of the hydroxyl groups,^15,16^ but is unique in that most other hydroxylated polymers do not show this function.^17^ Antifreeze (glyco)proteins, in contrast, have hydrophobic segments that bind to ice (rather than the glycan hydroxyls),^18−20^ underpinning the concept that a molecular mechanisms can lead to similar measurable macroscopic effects.

Considering the success of mimicking the macroscopic property of ice recrystallization inhibition, there remain remarkably few nonprotein ice nucleators, none of which have been designed from the bottom-up. Silver iodide, which has a very similar structure to hexagonal ice, is probably the best known.^21,22^ Feldspar (an aluminosilicate) is a potent ice nucleator,^23^ and its function appears to be caused by defects rather than the bulk structure.^24^ Graphene nanomaterials can also nucleate ice,^25^ and the size of the flakes proves crucial.^26^ The critical ice nucleus size is predicted to be ∼20 nm for nucleation at −5 °C,^27^ supporting the need for aggregates or molecularly large species. Arrays of cholesterol crystals^28^ and fractions of lignin (a heterogeneous material) have also been reported to have some activity,^29^ alongside marine biogenic particles.^30^ Ice-nucleating macromolecules of protein and polysaccharide origin have also been reported,^27,31^ but their chemical structures remain unknown.

In general, effective ice nucleators are thought to have a good lattice match to ice, meaning that water molecules bind to the nucleator in spatial locations similar to those they would occupy in ice. According to classical nucleation theory (CNT), the barrier to ice nucleation is due to the energetic cost of forming an interface between supercooled water and ice. By templating ice, a nucleator allows the formation of a smaller ice critical cluster reducing the energy barrier to nucleation and causing ice to form at warmer temperatures. For ice nucleation to occur at warmer temperatures, more significant nucleation sites possessing a lattice match to ice, or at least an ability to bind water in an icelike structure, are required.^3,27^

There are currently no design rules for ice nucleators, but some key observations have been made. There is evidence that when the size of an AFP increases, it can become an INP supported by experimental^32^ and modeling evidence.^3^ Small IBPs (∼2 nm) nucleate ice at cold temperatures, near to where homogeneous ice nucleation occurs^32^ but ice-nucleating proteins (INPs) maintain a coherent lattice match to ice across nucleation sites spanning as many as 30 individual INPs, nucleating ice at −2 °C:^3^ This corresponds to 50 nm x 50 nm nucleation sites. Ogawa et al. reported that linear poly(vinyl alcohol), which is known to bind ice, is capable of nucleating ice at temperatures a few degrees above the homogeneous nucleation temperature but did not show a molecular-weight effect in the range tested.^33^ Taken together, the above shows an exciting possibility that a chemically defined synthetic ice binder could be engineered to become an ice nucleator if sufficient size, density, and placement of the ice binders can be accessed. A synthetic ice nucleator would enable the first true structure–property relationships to be determined, without using heterogeneous mixtures.

Here, we introduce an ice nucleator platform based on well-defined poly(vinyl alcohol) molecular brushes obtained *via* a combination of ring-opening metathesis polymerization (ROMP) and reversible addition–fragmentation chain-transfer (RAFT) polymerization, using “grafting-to” and “grafting-through” approaches. We demonstrate that densely grafted PVA bottlebrush polymers exhibit ice nucleation activity and are the first example of a synthetic polymer ice nucleator. Lower-density brush copolymers had reduced activity, demonstrating that confinement of the PVA is essential. These results show that synthetic ice nucleators can be accessed by macromolecular engineering.

## Experimental Section

### Materials

All chemicals were used as supplied unless otherwise stated. ROMP catalyst G3 ((H_2_IMes)(pyr)_2_(Cl)_2_Ru = CHPh), *cis*-5-norbornene-*exo*-2,3-dicarboxylic anhydride (95%), *exo*-5-norbornene carboxylic acid (97%), *N*-boc-ethylenediamine (≥98%), 1-hydroxypyrrolidine-2,5-dione (98%), ethyl vinyl ether (EVE) (≥99%), trifluoroacetic acid (TFA, 99%), mPEG_10K_-succinimidyl carboxymethyl ester (NHS-mPEG_227_, *M_n_* ∼ 10,000 Da), poly(ethylene glycol) methyl ether methacrylate (PEGMA_20_, *M_n_* ∼ 1000 Da), 4-cyano-4-(phenylcarbonothioylthio)pentanoic acid (97%), 4,4′-azobis(4-cyanovaleric acid) (ACVA, ≥98%), poly(vinyl alcohol) (*M_n_* ∼ 146,000–186,000 Da, ≥99% hydrolyzed), hydrazine hydrate (reagent grade, 50–60%), lithium aluminum hydride solution (LiAlH_4_, 1.0 M in THF), 1-hydroxybenzotriazole hydrate (HOBt, ≥97%), triethylamine (TEA, ≥99%), PBS (tablets), and sucrose (≥99.5%) were purchased from Sigma-Aldrich. The monomer vinyl acetate (VAc, ≥99%) was also purchased from Sigma-Aldrich and passed through a column of basic alumina to remove inhibitors prior to use. Potassium ethyl xanthate (98%) was obtained from Alfa Aesar. *N*-(3-Dimethylaminopropyl)-*N*′-ethylcarbodiimide hydrochloride (EDC.HCI, >98%) was purchased from Carbosynth. 2-Bromo-2-methyl-propionic acid (≥98%) and lanolin (anhydrous, USP) was purchased from Acros Organics. Paraffin oil (technical grade) was purchased from VWR International. Solvents of toluene (anhydrous, ≥99.8%), dichloromethane (DCM, anhydrous, ≥99.8%), *N*,*N*-dimethylformamide (DMF, anhydrous, ≥99.8%), and tetrahydrofuran (THF, anhydrous, ≥99.9%) were also purchased from Sigma-Aldrich. Dialysis membranes (MWCO = 3.5/300 kDa) were purchased from Spectra/Por. Formvar-carbon-coated (300 mesh) copper grids were purchased from EM Resolutions. Photo-polymerization reactions of vinyl acetate were conducted using an EvoluChem PhotoRedOx Temperature Controlled Box fitted with an EvoluChem LED spotlight (P201-18-2 450–455 nm) with total irradiance of 30 mW cm^–2^ and light beam angle of 25° operating at a wavelength of λ = 450–455 nm. Ultrapure water used for buffers was of Milli-Q grade (18.2 mΩ resistance).

### Photo-Polymerization of Vinyl Acetate Using 2-(Ethoxycarbonothioylthio)-2-methylpropanoic Acid NHS-Ester

2-(Ethoxycarbonothioylthio)-2-methylpropanoic acid NHS-ester (0.10 g, 0.33 mmol, 1 eq) and vinyl acetate (VAc) (8.46 g (9.06 mL), 98.2 mmol, 300 eq) were dissolved in 2.1 mL of dioxane in a 20 mL vial (80% w/w solids content). The resulting solution was degassed by sparging with N_2_(g) for 30 min, and the sealed vial was incubated at 37 °C with magnetic stirring under 460 nm light irradiation for 8 h. After that time, polymerization was quenched by removing the sealing and exposing it to air. An aliquot of crude polymerization mixture was withdrawn for ^1^H NMR in CDCl_3_ for conversion and *M*_*n*,NMR_ analysis. The reaction was rapidly cooled in liquid nitrogen and precipitated into diethyl ether. The polymer was reprecipitated into hexane from THF twice to yield a pale-yellow sticky polymer product that was further dried under vacuum. *M*_*n*,NMR_ was calculated by end-group analysis by comparing the integrations of the −(C***H***_**2**_)_2_ signals (s, 2.83 ppm) of NHS group with those of the corresponding signals of the −C***H*** signal (d, 3.69–4.02 ppm) of the polymer backbone. ^1^H NMR (400 MHz, CDCl_3_): δ (ppm) = 5.05–4.76 5.05–4.76 (br s, 210H, CH_2_C***H***O of polymer backbone), 2.83 (s, 4H, C***H***_**2**_C***H***_**2**_ of NHS), 2.17–1.94 (br m, 630H, OC(O)C***H***_**3**_ of polymer side chain), 1.94–1.58 (br m, 420H, C***H***_**2**_CHO of polymer backbone), 1.34 (t, 3H, C***H***_**3**_CH_2_O). Conversion = 69%, *M*_*n*,NMR_ = 18400 g mol^–1^ (DP_PVAc,NMR_ = 210). SEC (5 mM NH_4_BF_4_ in DMF) *M*_*n*,SEC RI_= 22800 g mol^–1^, *Đ*_M,SEC RI_ = 1.6.

### Synthesis of Poly(amino exo-norbornene imide), P(NB-NH_2_) Homopolymer Precursors *via* Ring-Opening Metathesis Polymerization (ROMP)

A typical procedure for the synthesis of P(NB-NBoc)_50_ homopolymer *via* solution ROMP is described. A stock solution of 100 mg/mL of G3 in CHCl_3_ and a solution of 173 mg of NB-NBoc (50 eq, 0.57 mmol) in 3.3 mL of CHCl_3_ were first prepared. Then, 100 μL of G3 stock solution (10 mg, 1 eq, 0.011 mmol) was added to the vial of NB-NBoc monomer solution with rapid stirring, and polymerization was allowed to proceed at room temperature for 1 h (final [NB-NBoc] = 50 mg/mL, final [G3] = 2.9 mg/mL). The polymerization was then quenched by the addition of a few drops of ethyl vinyl ether, and P(NB-NBoc)_50_ homopolymer was precipitated from diethyl ether, isolated by vacuum filtration, and dried under vacuum prior to ^1^H NMR and SEC analyses. The procedure was repeated for [NB-NBoc]/[G3] ratios of 100, 200, and 400. ^1^H NMR (400 MHz, CDCl_3_) *M*_*n*,theo._ = 15,300 g mol^–1^. SEC (DMF + 5 mM NH_4_BF_4_) *M*_*n*,SEC_ = 17,600 g mol^–1^, *Đ*_M,SEC_ = 1.10.

Next, P(NB-NBoc)_50_ homopolymer (100 mg, 0.003 mmol) was dissolved in a 1:1 TFA/DCM solution (5 mL) and stirred at room temperature for 4 h. The polymer reaction mixture was concentrated to dryness under reduced pressure to afford a brown residue that was precipitated by the addition of diethyl ether. Removal of the solvent resulted in the isolation of the amino-deprotected polymer product as a brown solid (P(NB-NH_2_)50). The resulting polymer was then dissolved in DI water and dialyzed against DI water (dialysis membrane MWCO = 3.5 kDa) for approximately 14 h and lyophilized to afford the polymer products as brown solid. The procedure was repeated for P(NB-NBoc)100, 200, and 400 homopolymers. SEC characterization of the resulting deprotected poly(amino exo-norbornene imide) polymers was not possible due to high sample–column interactions. ^1^H NMR (400 MHz, DMSO-*d*_6_): δ (ppm) = 8.04–7.92 (br s, CH_2_N***H_2_*** of polymer side chain), 5.73–5.56 (br m, C***H***CHCH_2_ of polymer backbone), 5.56–5.33 (br m, C***H***CHCH_2_ of polymer backbone), 3.74–3.48 (br s, CH_2_C***H_2_***NH_2_ of polymer side chain), 3.48–3.24 (br m, C***H_2_***CH_2_NH_2_ of polymer side chain), 3.24–2.83 (br s, C(O)C***H***C***H*** C(O)of polymer backbone), 2.83–2.56 (br s, C***H***CH_2_C***H*** of polymer backbone), 2.13–1.80 (br s, CHC***H_2_***CH of polymer backbone), 1.62–1.32 (br s, CHC***H_2_***CH of polymer backbone).

### Synthesis of P((P(NB-NH)-*g*-PVA_210_)-*stat*-P(NB-NH_2_))_n_ Graft Copolymers *via* “Grafting-To” Approach

A representative synthesis of P((P(NB-NH)-*g*-PVA_210_)-_stat_-P(NB-NH_2_))_50_ by “grafting-to” is as follows: NHS-PVAc_210_ (2.0 mg, 0.0002 mmol, 1 eq), P(NB-NH_2_)_50_ (0.34 mg, 0.018 mmol, 100 eq), and EDC.HCl (3.5 mg, 0.018 mmol, 100 eq) were dissolved in 6.5 mL of anhydrous DMF, followed by the addition of Et_3_N (2 μL, 0.018 mmol, 100 eq), and the reaction mixture was stirred at 70 °C for 16 h. An aliquot of the crude reaction mixture was then taken for SEC analysis in DMF (+0.1% LiBr). The resulting polymer mixture was precipitated twice in diethyl ether. The resulting P(NB-NH_2_)_50_-*g*-PVAc_210_ branched copolymer was then dissolved in methanol (2 mL) following by the addition of hydrazine hydrate solution (5 mL, 50–60% in water) in a stoppered vial. The reaction mixture was stirred at room temperature overnight. The resulting graft copolymer was then dissolved in DI water and dialyzed against DI water (dialysis membrane MWCO = 300 kDa) for approximately 2 weeks to ensure full removal of unfunctionalized PVA210 and lyophilized to afford the polymer product as spongy pale-yellow solid. Procedure was repeated for the synthesis of P((P(NB-NH)-*g*-PVA_210_)-*stat*-P(NB-NH_2_))100, 200, 400 and control sample P((P(NB-NH)-*g*-PEG_227_)-*stat*-P(NB-NH_2_))_200_. Complete hydrolysis was confirmed by ^1^H NMR analysis in DMSO-*d*_6_. ^1^H NMR (400 MHz, DMSO-*d*_6_): δ (ppm) = 4.74–4.63 (br s, CH_2_CHO***H*** of polymer side chain), 4.58–4.41 (br s, CH_2_CHO***H*** of polymer side chain), 4.32–4.18 (br m, CH_2_CHO***H*** of polymer side chain), 3.99–3.73 (br m, CH_2_C***H***OH of polymer backbone), 1.71–1.14 (br m, C***H_2_***CHOH of polymer backbone).

### Photo-Polymerization of Vinyl Acetate Using NB-CTA

2-(Ethoxycarbonothioylthio)-2-methylpropanoic acid exo-5-norbornene-2-methylamide (0.10 g, 0.32 mmol, 1 eq) and vinyl acetate (VAc) (8.24 g (8.82 mL), 95.7 mmol, 300 eq) were dissolved in 2.1 mL of dioxane in a 20 mL vial (80% w/w solids content). The resulting solution was degassed by sparging with N_2_(g) for 30 min, and the sealed vial was incubated at 37 °C with magnetic stirring under 460 nm light irradiation for 8 h. After that time, polymerization was quenched by removing the sealing and exposing it to air. An aliquot of crude polymerization mixture was withdrawn for ^1^H NMR in CDCl_3_ for conversion and *M*_*n*,NMR_ analysis. The reaction was rapidly cooled in liquid nitrogen and precipitated into diethyl ether. The polymer was re-precipitated into hexane from THF twice to yield a pale-yellow sticky polymer product that was further dried under vacuum. *M*_*n*,NMR_ was calculated by end-group analysis by comparing the integrations of the −(C***HCH***) signals (s, 6.28 ppm) of norbornene group with those of the corresponding signals of the −C***H*** signal (d, 3.69–4.02 ppm) of the polymer backbone. ^1^H NMR (400 MHz, CDCl_3_): δ (ppm) = 6.28 (s, 2H, C***H***C***H*** of norbornene end group), 5.05–4.76 (br s, 208H, CH_2_C***H***O of polymer backbone), 2.83 (s, 4H, C***H***_**2**_C***H***_**2**_ of NHS), 2.17–1.94 (br m, 630H, OC(O)C***H***_**3**_ of polymer side chain), 1.94–1.58 (br m, 420H, C***H***_**2**_CHO of polymer backbone), 1.34 (t, 3H, C***H***_**3**_CH_2_O). Conversion = 67%, *M*_*n*,NMR_ = 18,400 g mol^–1^ (DP_PVAc, NMR_ = 208). SEC (5 mM NH_4_BF_4_ in DMF) *M*_*n*,SEC RI_ = 17,900 g mol^–1^, *Đ*_M,SEC RI_ = 1.40.

### Synthesis of PNB_n_-*g*-PVA_208_ Bottlebrush Polymers *via* ROMP “Grafting-Through” Approach

A representative synthesis of a PNB_20_-*g*-PVAc_208_ bottlebrush polymer by ROMP “grafting-through” is as follows: NB-PVAc_208_ (100 mg, 0.006 mmol, 20 equiv) was dissolved in 1.9 mL of anhydrous THF in a 3 mL vial equipped with a magnetic stirring bar. To the vial was added 100 μL of a 2.0 mg mL^–1^ stock solution of G3 in THF (final [NB-PVAc_208_]/[G3] = 20, [NB-PVAc_208_] = 50 mg mL^–1^). The reaction mixture was stirred at room temperature for 2 h. The polymerization was then quenched *via* addition of a few drops of ethyl vinyl ether. An aliquot of the crude reaction mixture was then taken for SEC analysis in DMF (+0.1% LiBr). The resulting polymer, PNB_20_-*g*-PVAc_208_, was isolated by precipitation from diethyl ether and was dried under vacuum.

The resulting PNB_20_-*g*-PVAc_210_ (50 mg) bottlebrush polymer was then dissolved in methanol (0.5 mL) followed by the addition of and hydrazine hydrate solution (3 mL, 50–60% in water) in a stoppered vial. The reaction mixture was stirred at room temperature overnight. The resulting polymer was then dissolved in DI water and dialyzed against DI water (dialysis membrane MWCO = 300 kDa) for approximately 1 week to ensure full removal of unfunctionalized PVA**208** and lyophilized to afford the polymer products as a white solid. The procedure was repeated for the synthesis of PNB_n_-*g*-PVAc_210_ (*n* = 30, 40). Complete hydrolysis was confirmed by ^1^H NMR analysis in DMSO-*d*_6_. ^1^H NMR (400 MHz, DMSO-*d*_6_): δ (ppm) = 4.74–4.63 (br s, CH_2_CHO***H*** of polymer side chain), 4.58–4.41 (br s, CH_2_CHO***H*** of polymer side chain), 4.32–4.18 (br m, CH_2_CHO***H*** of polymer side chain), 3.99–3.73 (br m, CH_2_C***H***OH of polymer backbone), 1.71–1.14 (br m, C***H_2_***CHOH of polymer backbone).

### Polymerization of Poly(ethylene glycol) Methyl Ether Methacrylate Using 4-Cyano-4-(phenylcarbonothioylthio)pentanoic Acid

A vial was charged with 4-cyano-4-(phenylcarbonothioylthio)pentanoic acid (5.0 mg, 0.018 mmol, 1 eq), PEGMA_20_ (3.58 g, 3.6 mmol, 200 eq), ACVA (1.0 mg, 0.004 mmol, 0.2 eq), and 17 mL of DMF. The vial was then sealed and deoxygenated using three successive cycles of freeze–pump–thaw to remove O_2_(g). The vial was placed into an aluminum heating block, which had been preheated to 70 °C to initiate polymerization. After 24 h, the polymerization was quenched by exposing the vial to air and submerging it into liquid N_2_. An aliquot was withdrawn for the determination of monomer conversion by ^1^H NMR spectroscopy. The polymer was precipitated into diethyl ether from dioxane twice to yield a white polymer product that was further dried under vacuum. ^1^H NMR (400 MHz, methanol-*d*_4_): δ (ppm) = 3.88–3.51 (br s, 16,000H, OC***H***_**2**_C***H***_**2**_ of polymer side chain), 2.48–1.58 (br m, 400H, C(CH_3_)C***H***_**2**_ of polymer backbone), 1.41–0.76 (br m, 600H, C(C***H***_**3**_)CH_2_ of polymer backbone), *M*_*n*,theo_ = 200,000 g mol^–1^. SEC (5 mM NH_4_BF_4_ in DMF) *M*_*n*,SEC RI_ = 134,300 g mol^–1^, *Đ*_M,SEC RI_ = 2.0.

## Results and Discussion

**Figure 1:**
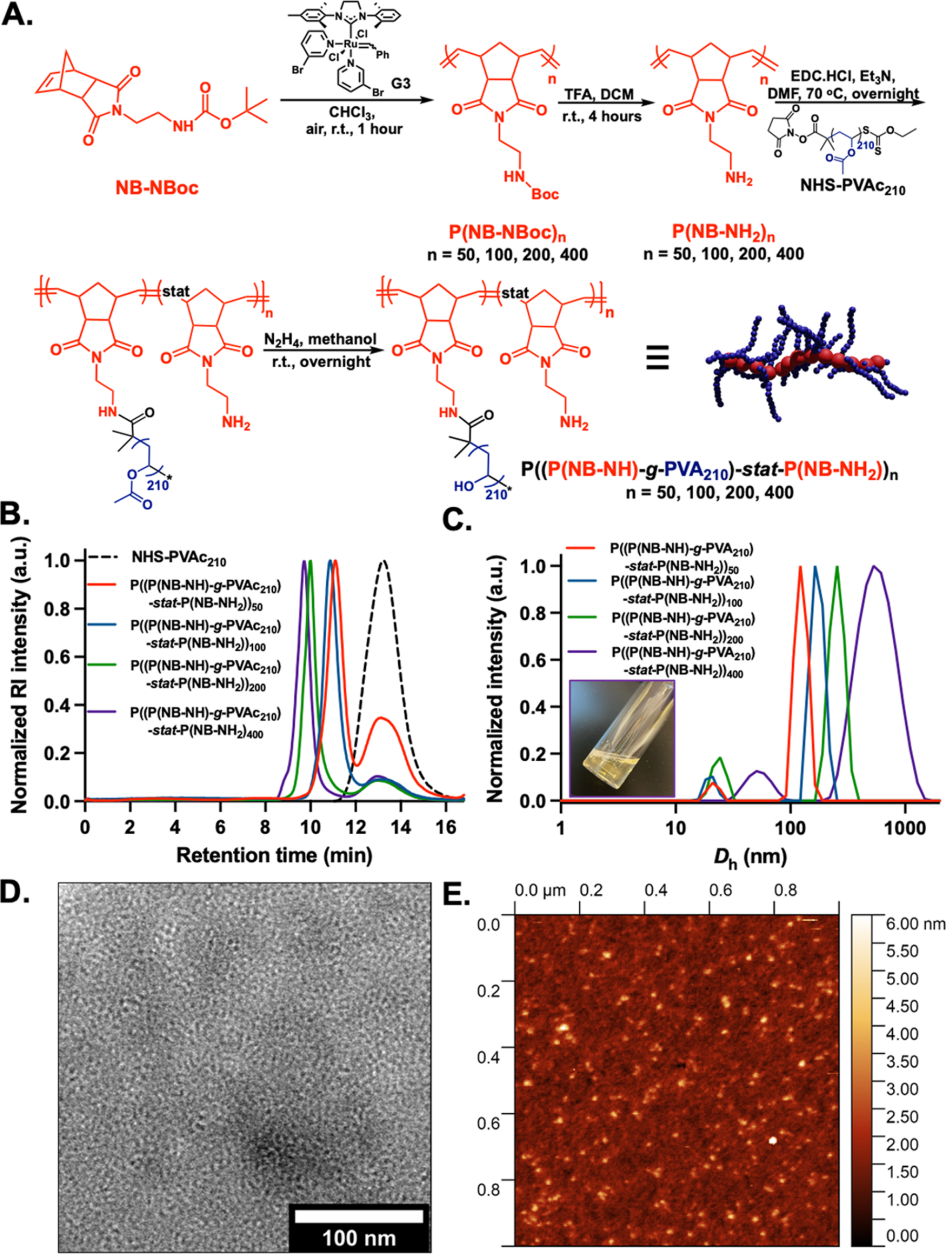
(A) Design and synthesis of graft PVA-based brush copolymers generated *via* “grafting-to” procedure. Schematic representation of the synthetic route followed for the preparation of P((P(NB-NH)-*g*-PVA_210_)-*stat-*P(NB-NH_2_))_n_ (*n* = 50, 100, 200, 400) graft copolymers. (B) Normalized SEC-RI chromatograms for P((P(NB-NH)-*g*-PVAc_210_)-*stat*-P(NB-NH_2_))*_n_* copolymer precursors in DMF + 0.1% LiBr and solution characterization of resulting P((P(NB-NH)-*g*-PVA_210_)-*stat*-P(NB-NH_2_))*_n_* (*n* = 50, 100, 200, 400) in water *via* (C) DLS analysis (insets: representative photograph of 1 mg mL^–1^ aq solutions of P((P(NB-NH)-*g*-PVA_210_)-*stat*-P(NB-NH_2_))_400_). (D) Dry-state TEM image obtained from a 1 mg mL^–1^ solution of P((P(NB-NH)-*g*-PVA_210_)-*stat*-P(NB-NH_2_))_400_ in water. (E) Representative AFM image obtained from a 5 mg mL^–1^ solution of P((P(NB-NH)-*g*-PVA_210_)-*stat*-P(NB-NH_2_))_50_ graft copolymer in water.

Our initial hypothesis was that densely packed PVA side chains, to mimic the regular arrays of IBPs which lead to nucleation, were essential. A “grafting-to” approach was first employed, which results in low grafting densities due to steric repulsions between bulky side chains (Figure 1A).^34,35^ Poly(norbornene)s bearing primary amines were first synthesized *via* an *N*-Boc-protected intermediate monomer (NB-NBoc, Figures S1 and S2), initiated by Grubbs third-generation bispyridyl complex (G3), [M]/[G3] of 50, 100, 200, 400. Full characterization by ^1^H NMR and size exclusion chromatography (SEC) analysis is shown in Table 1 and Figures S17 and S18. Deprotection of P(NB-NBoc)*_n_* with trifluoroacetic acid (TFA) gave the desired poly(amino norbornene imide) P(NB-NH_2_)*_n_* homopolymers (Figure S19).

**Table 1:** Molecular Characteristics of P(NB-NBoc)*_n_* Prepared *via* Solution ROMP Using Different Initial [NB-NBoc]/[G3] Ratios, as Determined by ^1^H NMR Spectroscopy and SEC Analysis

[NB-NBoc]/[G3]	% conv.^a^	*M*_*n*,theo._ (kDa)^b^	*M*_*n*,SEC_ (kDa)^c^	*Đ*_M,SEC_^c^
50	>99	15.3	17.6	1.11
100	>99	30.6	34.5	1.11
200	>99	61.3	61.4	1.15
400	>99	122.4	103.8	1.23

a Monomer conversion calculated from ^1^H NMR spectroscopy in CDCl_3_.

b Calculated from conversion.

c *M_n_* and *Đ*_M_ values calculated from PMMA standards using DMF + 5 mM NH_4_BF_4_ as the eluent.

ω-*N*-Hydroxysuccinimide-poly(vinyl acetate) (NHS-PVAc) was then prepared by photo-RAFT/MADIX to maximize end-group fidelity and was subsequently grafted to P(NB-NH_2_) homopolymers. See the Synthetic Procedures section in the Supporting Information (NHS-PVAc_210_, Conv. = 69%, *M*_*n*,SEC RI_ = 22.8 kDa, *Đ*_M_ = 1.56) (Figures S3–S7, Table 2).^36,37^ Following isolation, P((P(NB-NH)-*g*-PVAc_210_)-*stat*-P(NB-NH_2_))*_n_* (*n* = 50, 100, 200, 400) graft copolymers were characterized by SEC analysis revealing bimodal peaks at high-molecular-weight direction. Figure 1B shows corresponding SEC curves of the NHS-PVAc_210_ side chain (dashed black curve) and the resultant graft copolymers, in which the peak positions of the graft copolymers completely shifted to higher molecular weights. A significant amount of unfunctionalized NHS-PVAc_210_ was also apparent for all graft copolymer formulations. Grafted side chains (%) of the resulting graft copolymers from SEC analysis were estimated to be ca. 20–40% (Table 2), due to steric repulsions between bulky side chains, limiting grafting density.^34,35^ The acetate protecting groups were quantitively removed by an excess of hydrazine hydrate to yield P((P(NB-NH)-*g*-PVA_210_)-*stat*-P(NB-NH_2_))*_n_* graft copolymers that were purified by dialysis (MWCO = 300 kDa) to remove unfunctionalized PVA_210_ and characterized by ^1^H NMR and FTIR spectroscopic analysis (Figures S20 and S21, Supporting Information).

**Table 2:** Molecular Characteristics of P((P(NB-NH)-*g*-PVAc_210_)-*stat*-P(NB-NH_2_))*_n_* (*n* = 50, 100, 200, 400) Graft Copolymers as Determined by SEC Analysis^c^

sample	*M*_*n*,SEC_ (kDa)^a^	*Đ*_M,SEC_^a^	grafted side chains (%)^b^
NHS-PVAc_210_	22.8	1.56	------
P((P(NB-NH)-*g*-PVAc_210_)-*stat*-P(NB-NH_2_))_50_	378.6	1.10	33.0
P((P(NB-NH)-*g*-PVAc_210_)-*stat*-P(NB-NH_2_))_100_	557.4	1.23	24.0
P((P(NB-NH)-*g*-PVAc_210_)-*stat*-P(NB-NH_2_))_200_	1013.4	1.21	22.0
P((P(NB-NH)-*g*-PVAc_210_)-*stat*-P(NB-NH_2_))_400_	1539.4	1.44	17.0

a *M_n_* and *Đ*_M_ values calculated from PMMA standards using DMF + 0.1% LiBr as the eluent.

b Calculated from SEC curves of reaction mixture before and after modification.

c Grafted side chains (%) = (*M*_*n*,SEC RI_, _graft copolymer_ – (*M*_*n*,P(NB-NH2), theo_)/*M*_*n*,SEC RI, NHS-PVAc210_).

The solution behavior of the resulting PVA graft copolymers was investigated by dynamic light scattering (DLS), transmission electron microscopy (TEM), and atomic force microscopy (AFM). DLS analysis showed that graft copolymer samples exhibited an increase in length upon increasing the backbone of P(NB-NH_2_)*_n_* (Figure 1C). The fact that these traces are multimodal can be rationalized by considering additional relaxations occurring, which contributed to the scattering profile due to the anisotropy of the bottlebrush polymers in solution.^38^ Dry-state TEM images of P((P(NB-NH)-*g*-PVA_210_)-*stat*-P(NB-NH_2_))*_n_* confirmed the presence of small cylindrical nanostructures (Figures 1D and S22). Importantly, no large aggregates were observed in the TEM images, indicating that neither graft copolymers were spontaneously self-assembled into higher-order structures when dissolved in aqueous media at room temperature. AFM analysis also showed the formation of some spherical particles (Figures 1E and S23). While the PVA graft copolymers appear to be spherical in the AFM images, they likely exist as cylindrical nanostructures in aqueous environments (as confirmed by TEM analysis) due to their anisotropic dimensions, as has been reported for brush polymers in solution measured using X-ray or neutron scattering analyses.^39,40^ Moreover, brush polymers have been shown to de-wet various substrates due to unfavorable surface interactions, causing them to present as globules in AFM images.^13,41^

**Figure 2:**
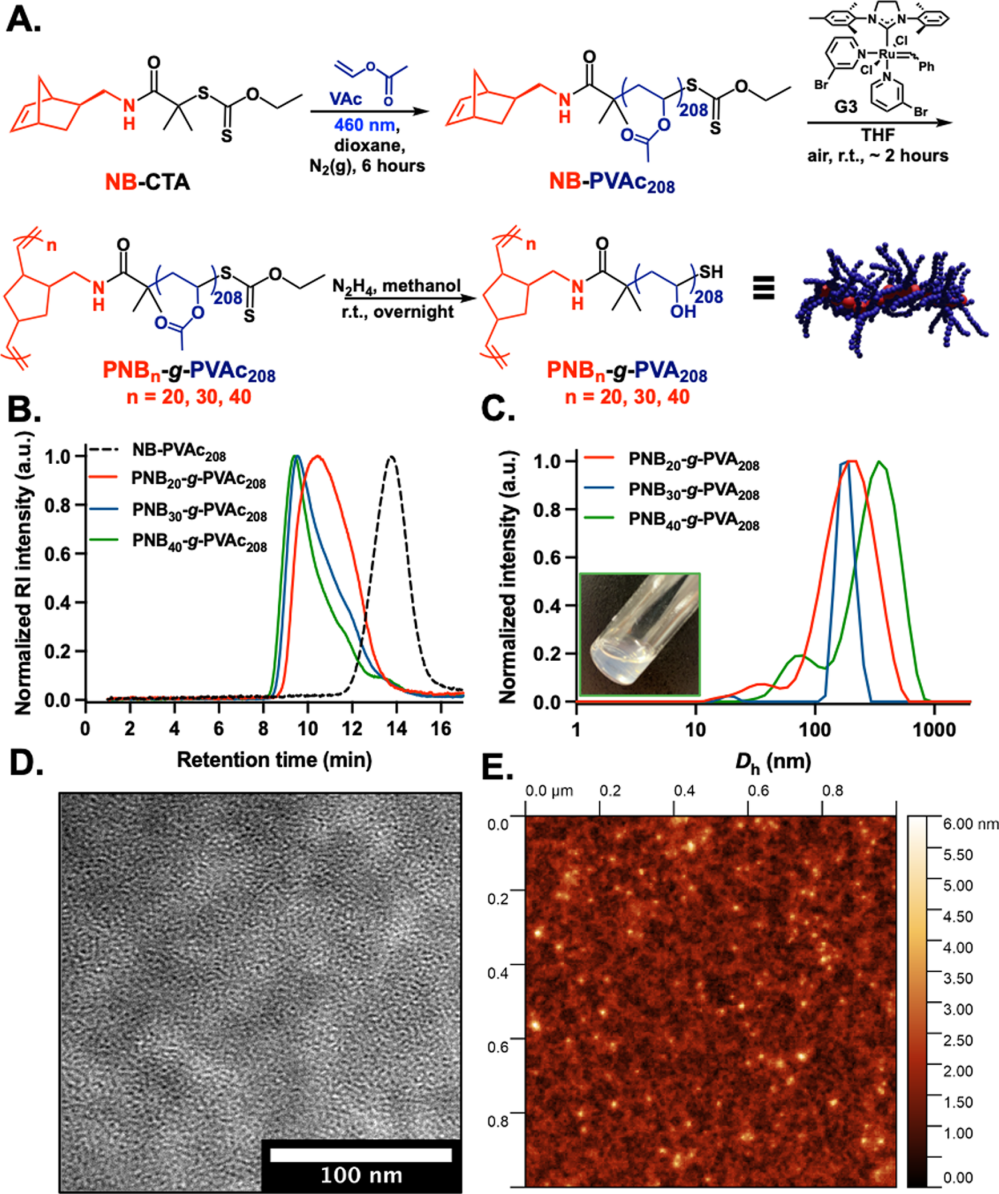
(A) Design and synthesis of dense PVA-based brush copolymers generated *via* “grafting-through” procedure. Schematic representation of the synthetic route followed for the preparation of PNB*_n_*-*g*-PVA_208_ (*n* = 20, 30, 40) bottlebrush polymers. (B) Normalized SEC-RI chromatograms for PVAc bottlebrush precursors in DMF + 0.1% LiBr and solution characterization of resulting PVA bottlebrushes in water *via* (C) DLS analysis (inset: representative photograph of 1 mg mL^–1^ aq solutions of PNB_40_-*g*-PVA_208_). (D) Dry-state TEM images obtained from a 1 mg mL^–1^ solution of PNB_40_-*g*-PVA_208_ in water; (E) representative AFM images obtained from a 5 mg mL^–1^ solution of PNB_20_-*g*-PVA_208_ bottlebrush polymer in water.

Next, a series of high-density PVA bottlebrush polymers were prepared using ROMP “grafting-through” approach using a norbornene-end poly(vinyl acetate) macromonomer (NB-PVAc), Figure 2A. *Exo*-5-norbornene-2-methylamine was synthesized in this case (Figures S8–S15; see the Synthetic Procedures section in the Supporting Information), as it was demonstrated by Matson and co-workers that the choice of anchor group dramatically affects overall ROMP propagation rates with norbornene anhydride-based macromonomers leading to poorer chain extensions and conversions when targeting high *M*_w_ bottlebrushes.^42,43^

A norbornyl xanthate chain-transfer agent (NB-CTA) of 2-(ethoxycarbonothioylthio)-2-methylpropanoic acid *exo*-5-norbornene-2-methylamide was synthesized and used to prepare an NB-PVAc_208_ macromonomer *via* photo-initiated RAFT/MADIX polymerization (NB-PVAc_208_, Conv. = 67%, *M*_*n*,SEC RI_ = 17.9 kDa, *Đ*_M_ = 1.4). Bottlebrush polymers were synthesized through ROMP of the norbornene amide end group on the PVAc macromonomer by varying the molar ratio of G3 catalyst ([NB-PVAc_208_]/[G3]) to achieve PNB*_n_*-*g*-PVAc_208_ bottlebrush polymers (*n* = 20, 30 and 40).

SEC analysis revealed low-molecular-weight shoulders, which may correspond to unreacted NB-PVAc_208_ macromonomer. However, the long low-molecular-weight fraction could also be explained by the premature termination of ROMP polymers. Large *Đ*_M_ values can be primarily attributed to the employment of a high *M*_w_ of PVAc macromonomer with a relatively large dispersity (Figure 2B and Table 3) as well as the above-mentioned premature termination events. In addition, number-average molecular weight values (*M_n_*) determined by SEC analysis were evidently lower than theoretically expected values determined from polymerization conversions. This discrepancy in theoretical and observed *M_n_* likely originates from the branched nature of the bottlebrush polymers, leading to reduced hydrodynamic volumes relative to linear polymers of the same molecular weight and thus increased retention on the SEC columns. Acetate protecting groups were removed by hydrazine hydrate, as confirmed by ^1^H NMR and FTIR analyses (Figures S24 and S25) to yield PNB*_n_*-*g*-PVA_208_ (*n* = 20, 30, 40) bottlebrush polymers that were dialyzed (MWCO = 300 kDa) to ensure removal of unreacted NB-PVA_208_ macromonomer.

**Table 3:** Molecular Characteristics of PNB*_n_*-*g*-PVAc_208_ (*n* = 20, 30, 40) Bottlebrush Polymers Prepared *via* ROMP Using Different Initial [NB-PVAc208]/[G3] Ratios, as Determined by ^1^H NMR Spectroscopy and SEC Analysis

[NB-PVAc_208_]/[G3]	% conv.^a^	*M*_*n*,theo._ (kDa)^b^	*M*_*n*,SEC_ (kDa)^c^	*Đ*_M,SEC_^c^
20	>99	358.0	162.6	2.11
30	>99	537.0	241.1	2.41
40	>99	716.0	340.3	2.39

a Monomer conversion calculated from ^1^H NMR spectroscopy in CDCl_3_.

b Calculated from conversion.

c *M_n_* and *Đ*_M_ values calculated from PMMA standards using DMF + 0.1% LiBr as the eluent.

DLS analysis also confirmed the formation of anisotropic particles and exhibited an increase in size upon increasing the backbone, as expected (Figure 2C). Dry-state TEM images of PNB*_n_*-*g*-PVA_208_ (*n* = 20, 30, 40) also confirmed the presence of small cylindrical nanostructures (Figures 2D and S26). AFM analysis again showed the formation of spherical particles, which is likely due to unfavorable surface interactions of PVA side chains with silicon surface, causing them to present as globules (Figures 5 and S27). Since PVA’s side-chain length is by far larger than bottlebrush backbones, it may appear spherical/ellipsoid, but further investigation is beyond the scope of this study as the extended local bottlebrush structure is shown (below) to be sufficient for the intended application.

**Figure 3:**
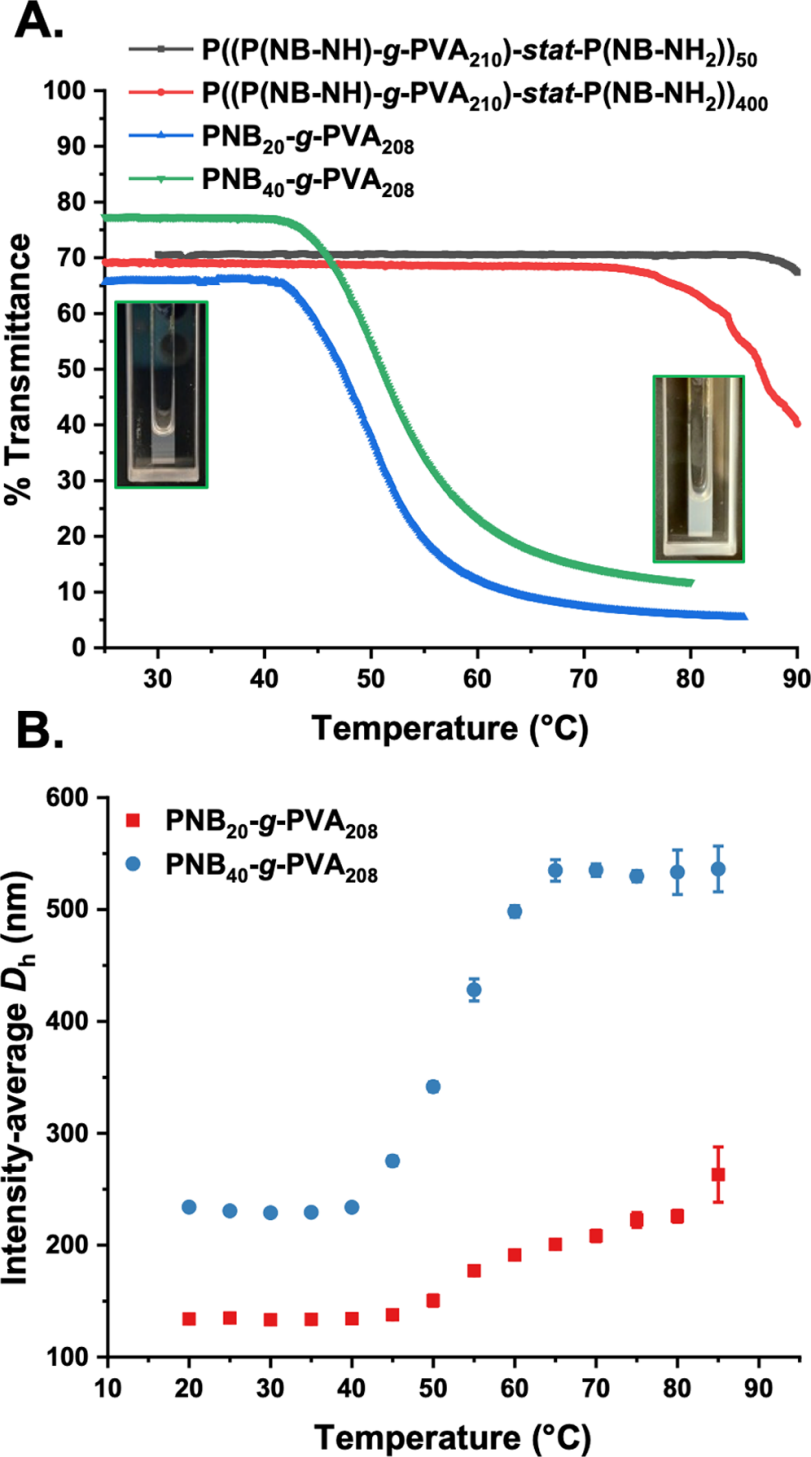
Evaluation of the thermoresponsive behavior for P((P(NB-NH)-*g*-PVA_210_)-*stat*-P(NB-NH_2_))*_n_* (*n* = 50, 400) and PNB*_n_*-*g*-PVA_208_ (*n* = 20, 40) bottlebrush polymers by (A) UV–vis spectroscopy and (B) variable-temperature DLS analysis. Samples were run from 20 to 85 °C at 1 mg mL^–1^ in water, with the UV–vis transmittance spectra recorded at λ = 700 nm (inset: UV–vis cuvettes containing 1 mg mL^–1^ solution of PNB_40_-*g*-PVA_208_ at 25 °C (left) before and after heating at 85 °C (right)).

During the handling of these materials, LCST (lower critical solution temperature) behavior was also observed for dense bottlebrush polymers prepared by ROMP “grafting-through”. This has not previously been reported, due to the absence of high-density PVA copolymers, and was hence explored. It is, however, anticipated that the backbone hydrophobicity for bottlebrushes prepared by “grafting-through” will lead to some self-assembly which may contribute to this behavior. Variable-temperature ultraviolet–visible (UV–vis) spectroscopy showed a cloud point of approximately 51 °C for PNB_20_-*g*-PVA_208_ and 54 °C for PNB_40_-*g*-PVA_208_ (Figure 3A). No apparent LCST behavior was observed for PVA graft copolymers prepared by “grafting-to” except for P((P(NB-NH)-*g*-PVA_210_)-*stat*-P(NB-NH_2_))_400_ that exhibited a decrease in transmittance at 75 °C, confirming that the side chain density is having a significant impact on the solution behavior of these polymers. DLS temperature ramp experiment for dense bottlebrush polymers showed a stepwise increase in the size of the dissolved nanostructures that occurred as the solution was heated above 40 °C (Figure 3B). Linear PVA homopolymers are known to possess LCST behavior in aqueous media close to water’s boiling point (∼100 °C), which is reduced by alkylation and increases with molecular weight, which agrees with these observations here.^44^ The close packing of polymer chains in this bottlebrush state facilitates the exclusion of water upon heating, lowering the temperature at which this transition occurs, and confirms that the solution properties compared to linear polymers are distinct.^45^

**Figure 4:**
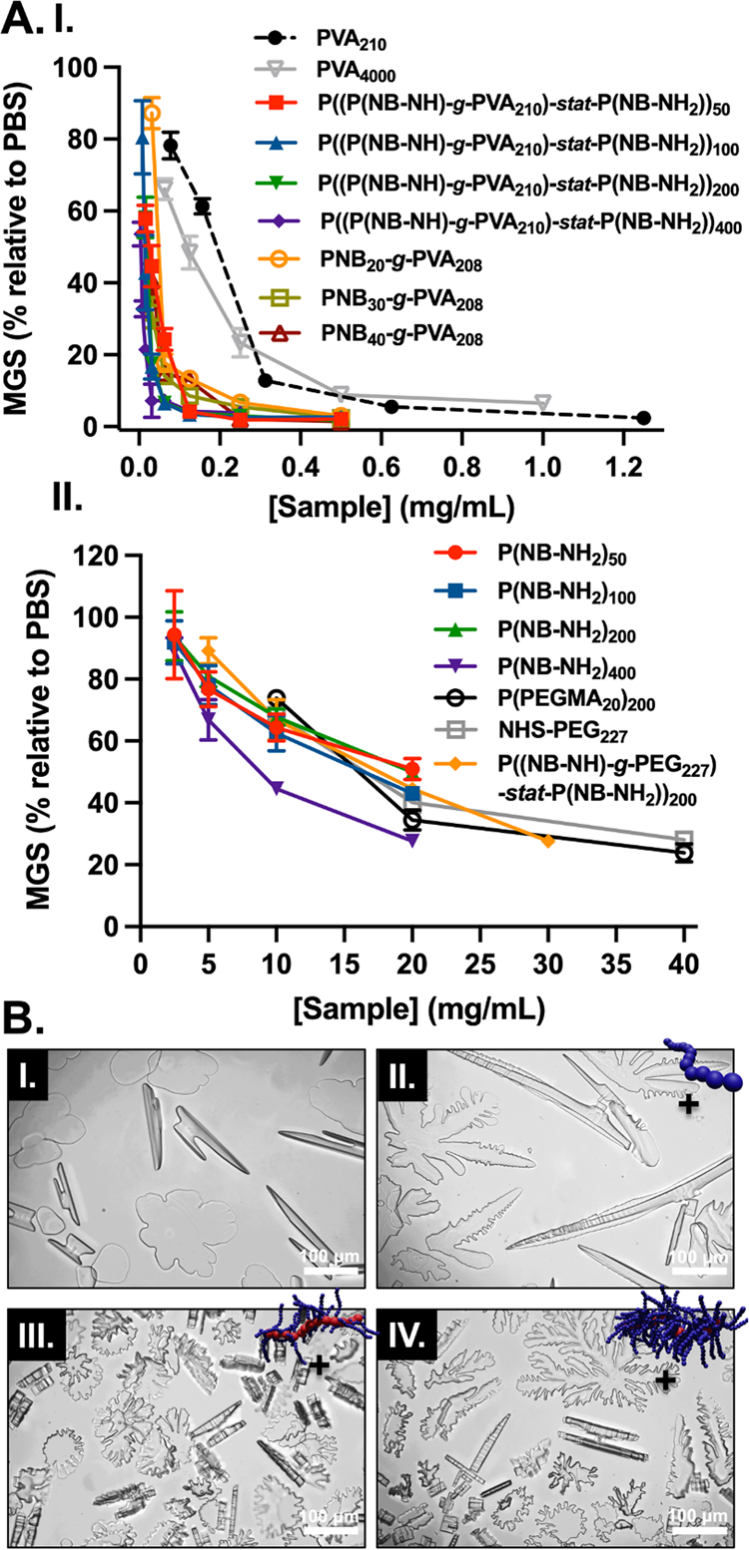
Assessment of ice recrystallization inhibition activity and ice-binding. (A) IRI activity summary of (I) P((P(NB-NH)-*g*-PVA_210_)-*stat-*P(NB-NH_2_))*_n_* (*n* = 50, 100, 200, 400) graft copolymers, PNB*_n_*-*g*-PVA_208_ (*n* = 20, 30, 40) bottlebrush polymers and (II) P(NB-NH_2_)*_n_* (*n* = 50, 100, 200, 400) and PEG-based control samples. Error bars are ±SD from a minimum of three repeats. The percent mean grain size (MGS) was reported relative to PBS control. (B) Modified “sucrose sandwich” ice shaping assay images for no additive (I) and 1 mg mL^–1^ of PVA_210_ (II), P((P(NB-NH)-*g*-PVA_210_)-*stat*-P(NB-NH_2_))_400_ (III) and PNB_40_-*g*-PVA_208_ (IV) in 45 wt % sucrose solution.

With this panel of bottlebrush polymers prepared, and their solution properties evaluated, the ice-binding interactions were then investigated, by assessment of their IRI (ice recrystallization inhibition) activity (Figure 4A). This was achieved by the “splat assay”, whereby small ice crystals are formed at −78 °C, then allowed to grow at −8 °C, and their growth was measured.^11,46^ Smaller MGS (mean grain size) means more ice recrystallization inhibition activity. Figure 4A(I) shows dose-dependent IRI of the polymer library vs controls of linear PVA. In all cases, there was a small but significant increase in IRI activity, with the bottlebrushes (high and low density) inhibiting below 0.1 mg mL^–1^. It has been previously reported that increasing the molecular weight of PVA increases IRI activity^11^ explaining these observations, although architecture-related enhancements cannot be fully ruled out. Voets and co-workers have previously prepared an acrylate-based PVA bottlebrush polymer using ATRP, which also showed no significant change in IRI compared to linear.^13^ In this previous work, there was no investigation of ice nucleation activity (see below). Note it was not possible to compare against linear PVA of the same MW, as linear PVA becomes increasingly insoluble in aqueous solutions at high MW, justifying our nonlinear synthetic approach to access PVA, which could not be obtained by conventional methods. The ROMP-derived backbones P(NB-NH_2_)*_n_* (*n* = 50, 100, 200, 400) were tested as a control (Figure 4A(II)) showing weak IRI. It should be noted that we have previously shown that the facial amphiphilicity of ROMP-derived polymers can lead to observable IRI activity. The magnitude of IRI of the control amino-functional polymers P(BN-NH_2_) was significantly less than this previous report but is not negligible.^47^

A PEG-based graft copolymer (as a non-PVA, nonlinear control) was also synthesized using an *N*-hydroxysuccinimide-poly(ethylene glycol) (NHS-PEG_227_) and grafted into the presynthesized poly(amino norbornene imide) P(NB-NH_2_)_200_ homopolymer to yield P((P(NB-NH)-*g*-PEG_227_)-*stat-*P(NB-NH_2_))_200_ (see the Synthetic Procedures section in the Supporting Information, Figures S28 and S29). A PEG methacrylate monomer (PEGMA_20_) was also employed to prepare P(PEGMA_20_)_200_ homopolymer (Figures S30 and S31). In both cases, nonlinear PEG controls were shown to have no appreciable IRI activity. Ice shaping (in sucrose solutions) showed that the PVA graft copolymers and bottlebrushes lead to more faceting of ice crystals, compared to linear PVA homopolymer (Figures 4B and S32–S35), as the large polymers can span multiple crystals as seen previously for PVA-grafted nanoparticles^14^ and AFP dendrimers.^48^ Single crystal assays (using a nanoliter osmometer) in contrast did not show shaping, suggesting that steric confinement does impact the ice/polymer interface (Figure S36, Supporting Information).

With the confirmation of ice binding (shown above), ice nucleation measurements could be undertaken. Pure liquid water can supercool to temperatures as low as −40 °C. It is thought that heterogeneous ice nucleators must template an ice critical cluster of a size predicted by classical nucleation theory (CNT).^32^ To achieve ice nucleation at −5 °C, a nucleator is predicted to support a cap-shaped cluster possessing a diameter of around 10 nm.^32^ As shown in Figures 1C and 2C, the bottlebrushes are above this size threshold.

**Figure 5:**
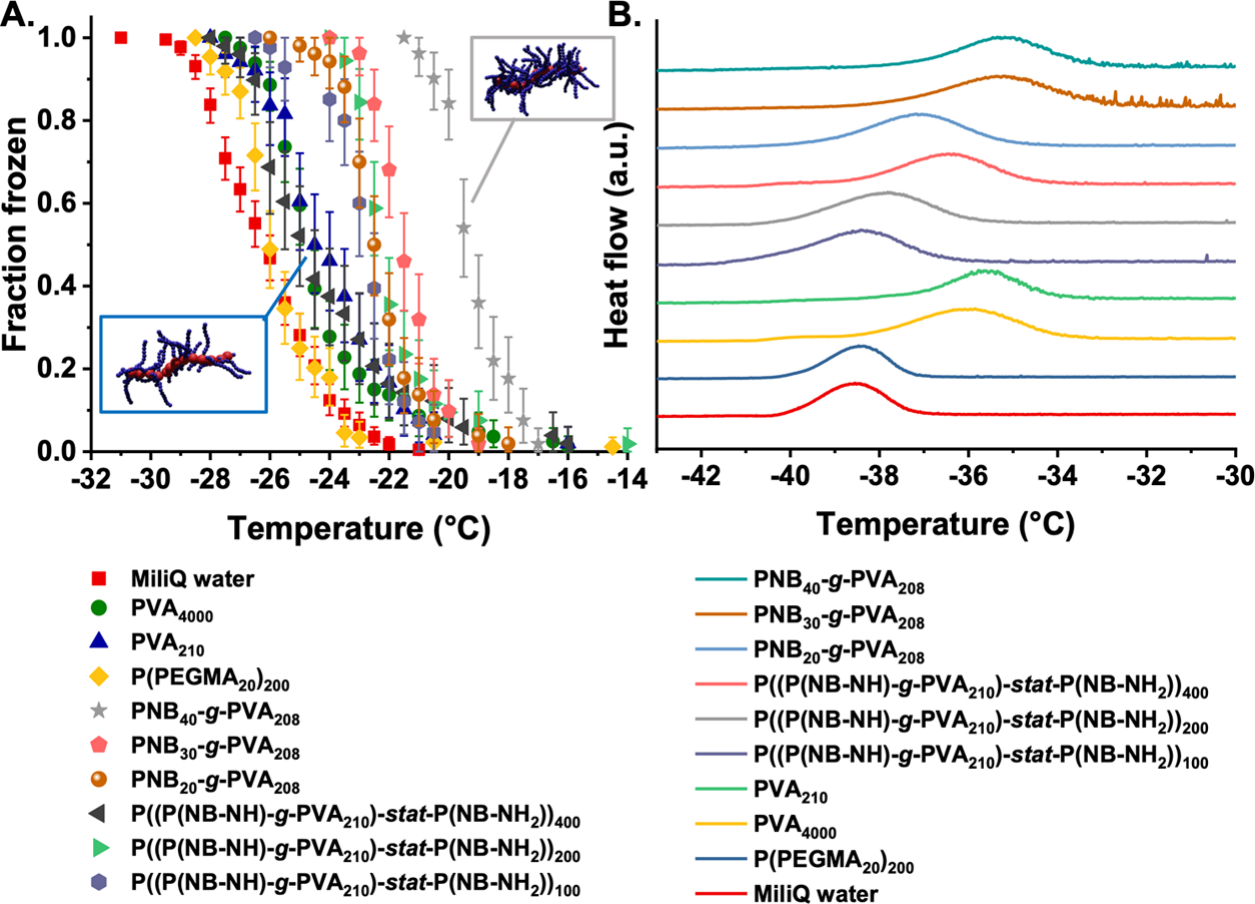
Assessment of ice nucleation activity of P((P(NB-NH)-*g*-PVA_210_)-*stat*-P(NB-NH_2_))*_n_* (*n* = 100, 200, 400) graft copolymers and PNB*_n_*-*g*-PVA_208_ (*n* = 20, 30, 40) bottlebrush polymers in (A) droplet fraction frozen for 1 μL water droplets containing various polymers. Confidence intervals were calculated using a Monte Carlo simulation described in the SI. (B) DSC analysis of picoliter water-in-oil emulsion droplets containing the polymers.

Two ice nucleation measurement techniques were employed here. To investigate the ice nucleation ability at temperatures close to the homogeneous freezing temperature, water-in-oil emulsion droplets of roughly 2 pL (Figures S37 and S38, Supporting Information) were frozen in a differential scanning calorimeter (DSC) by cooling at 1 °C/min, using a technique similar to that of Marcolli et al.^49^Figure 5B shows the DSC curves obtained, with the onset point of the exothermic peak caused by water crystallization defined as the ice nucleation temperature, *T*_f_. Marcolli et al. found a *T*_f_ of −36.5 °C for droplets for emulsions containing pure water droplets of roughly the same size, while Ogawa et al. found a*T*_f_ of −37.5 °C for a pure water emulsion of smaller droplets. We determined *T*_f_ for pure Milli-Q water to be −36.9 °C. This temperature is taken to represent the onset of homogeneous ice nucleation in our system. Similar emulsions containing 2 mg mL^–1^ linear and bottlebrush PVA samples were prepared so that each emulsion droplet will contain more than a million of even the largest bottlebrush. As shown in Figure 5 linear PVA_4000_ increased *T*_f_ by 3.5 °C compared to pure water (i.e Δ*T*_f_ = 3.5 °C). For linear PVA_210_, a Δ*T*_f_ of 3.2 °C was determined. This is qualitatively consistent with the findings of Ogawa et al., who found that the mass proportion of PVA present in an experiment determined the shift in nucleation temperature, rather than molar concentration (Figure S39).^33^ The Δ*T*_f_ observed for linear PVA is larger than that measured by Ogawa et al. who found a maximum value of approximately 2 °C for 2 mg mL^–1^ PVA solutions. The reason for this disparity is not clear. We observed that low-density bottlebrushes nucleated at lower temperatures than linear PVA, while high-molecular-weight high-density bottlebrushes nucleated at warmer temperatures, with a maximum Δ*T*_f_ of 4.5 °C for PNB_40_-*g*-PVA_208_, equivalent to a nucleation onset of −32.4 °C. For both high- and low-density bottlebrushes, a trend to higher nucleation temperatures was observed as the total molecular weight of the polymers increased, noting the large error in SEC of bottlebrushes.

To investigate the nucleation ability of larger quantities of the PVAs, microliter droplet freezing assays^50^ were conducted. In this assay, pure water droplets usually freeze at around −33 °C, above the homogeneous nucleation temperature. This is due to the inevitable presence of ice-nucleating impurities in the water and supporting substrates employed.^51^ As with the DSC measurements, 2 mg mL^–1^ solutions were prepared, and each droplet contains >10^12^ individual PVA molecules. The results are shown in Figure 5A.

Due to the variability of background measurements in this type of experiment, great care needs to be applied in interpreting results. The two linear PVAs (PVA_4000_ and PVA_210_) and P(PEGMA_20_)_200_ nucleated at temperatures slightly warmer temperatures than the instrument background. As neither of these polymers can bind ice, they provide a negative control for the PVA bottlebrushes. In contrast, the various PVA bottlebrushes were observed to all nucleate ice at temperatures higher than the freezing background and higher than the linear PVAs, showing that this macromolecular engineering approach to high-density PVA does lead to polymeric ice nucleating agents.

The less densely grafted P((P(NB-NH)-*g*-PVA_210_)-*stat-*P(NB-NH_2_))*_n_* (*n* = 50, 100, 200, 400) showed intermediate activity, while the densely grafted PNB*_n_*-*g*-PVA_208_ (*n* = 20, 30, 40) were more active, suggesting that dense presentation of PVA side chains is essential for nucleation activity—these trends are shown in Figure S39. With the much smaller droplets frozen in the DSC measurements, where each droplet contains molecules of the order of 10^6^, the low-density bottlebrushes nucleated ice less well than linear PVA, while the high-density bottlebrushes nucleated ice better than linear PVA.

High-density PNB_40_-*g*-PVA_208_ was observed to nucleate ice at average temperatures as warm as −19.2 °C, which is unprecedented for a fully synthetic soluble material. We have previously reported that large (>200 nm) polymer nanoparticles with a poly(vinyl pyrrolidone), PVP, corona also nucleated ice at similar temperatures.^52^ The large size and dense display in those particles may be related, but the particles did not have variable density coronas so the structure–property relationship was not clear. These particles did show weak ice binding (and IRI) and hence agree with the results here. Soluble extracts from pollen, which are thought to be polysaccharides, have been reported to nucleate ice, but they have not been characterized.^31^ It is worth noting that the largest bottlebrushes did show some aggregation in solution, so the contribution of a small number of larger aggregates is impossible to exclude, in this initial report.

There is evidence that ice nucleating protein activity is related to other ice-binding proteins based on their size and packing.^3,32,53^ The mechanism(s) of heterogeneous ice nucleation is not fully understood, even for the long-studied ice nucleation protein from *Pseudomonas syringae*. This is in part due to the challenge of studying membrane-anchored proteins, and that isolation of these as pure protein domains does not recapitulate the 3D assembly. Protein expression techniques only allow for linear extension (in a single protein), whereas this polymeric strategy provides defined macromolecules with multiple ice binding domains in a constrained manner, therefore providing experimental evidence that crowding and size are essential for nucleation, in agreement with modeling of IBPs.^3^ CNT suggests that a nucleation site must have a diameter of between 2 and 5 nm, to nucleate ice at −19 °C, with the size depending on the strength of the interaction with the ice critical cluster. The bottlebrushes are each at least 100 nm across and therefore larger than the ice cluster. It seems likely that the occasional patch of bottlebrush surface assumes a conformation that is sufficiently icelike to act as a nucleation site. In our ice nucleation experiments, we kept the mass of polymer per droplet the same while varying the size of the polymers.

This means that our experiments using larger bottlebrushes, which induce ice nucleation at higher temperatures, had less polymer surface area in contact with water. Simulations suggest that curved surfaces nucleate ice less well than flat surfaces.^54^ We tentatively suggest that the lesser curvature of the larger bottlebrushes increases the likelihood that they harbor an active site, and that for more rigid conformation, high-density bottlebrushes similarly favor the emergence of larger flat areas of icelike structure in the polymer surfaces. While the magnitude of ice nucleation activity observed here is much less than that for ice nucleating proteins, the ability to sequentially modify a bottom-up synthesized chemical nucleator will enable new avenues of discovery in the study of ice nucleators, not possible with biological materials, or the poorly defined inorganic nucleator.

## Conclusions

Here, we report a synthetic polymer capable of nucleating ice, inspired by how ice-binding proteins assemble into ice-nucleating structures. Our design was based on molecular bottlebrushes with (ice binding) PVA side chains, with the side-chain density tuned using ROMP to enable “grafting-through” (high density) or “grafting-to” (low density). The bottlebrush polymers were fully characterized, confirming the confinement of the PVA chains in the higher-density brushes, which also gave rise to LCST-type behavior, not seen for linear equivalents. The bottlebrush polymers were found to bind ice and retain their potent ice-recrystallization inhibition activity, with a slight increase relative to linear PVA. However, due to the limited solubility of high-MW linear PVA, this effect could be due to the size increase, rather than a pure architectural effect. Using ice nucleation assays, we observed that high-density PVA bottlebrushes significantly increased the ice nucleation temperature compared to other materials. Lower-density PVA brushes also favored nucleation in microliter droplet freezing assays, although they nucleated ice at colder temperatures than linear PVA in an emulsion freezing experiment using picoliter scale droplets. We hypothesize that larger bottlebrushes are more likely to possess an ice nucleation site due to their low degree of curvature and that the conformational rigidity of high-density bottlebrushes is again expected to favor the formation of flat, icelike water–polymer interfaces that can act as ice nucleation sites. The role of aggregates of these brushes could not be excluded. Our strategy of controlling side-chain density would be challenging with protein engineering tools alone. These materials may guide the development of new ice nucleators for cryobiology as synthetic probes to elucidate fundamental mechanisms of action and show that programmable ice nucleation from bottom-up synthesis may be achievable.

## Supporting Information Available

Materials and additional synthetic methods; experimental procedures; synthetic procedures; supplementary NMR, SEC, FTIR, DLS, TEM, and AFM data; and additional ice shaping/nucleation data (PDF)
