# Supplementary material for: Poly(vinyl alcohol) Molecular Bottlebrushes Nucleate Ice
Source: Biomacromolecules. 2022 Nov 28;23(12):5285–96. doi: 10.1021/acs.biomac.2c01097 (PMC9748936; doi:10.1021/acs.biomac.2c01097)
Supplement: Supplementary file 1 — bm2c01097_si_001.pdf [file bm2c01097_si_001.pdf]

Supporting Information

Poly(Vinyl Alcohol) Molecular Bottlebrushes Nucleate Ice

Panagiotis G. Georgiou,[1] Nina L. H. Kinney,[1] Ioanna Kontopoulou,[1] Alexander N. Baker,[1] Steven A. Hindmarsh,[3] Akalabya Bissoyi,[2] Thomas R. Congdon,[1] Thomas F. Whale,[1] and Matthew I. Gibson*[1,2]*

[1] Department of Chemistry, University of Warwick, Gibbet Hill Road, CV4 7AL, Coventry, UK

[2] Division of Biomedical Sciences, Warwick Medical School, University of Warwick, Gibbet Hill Road, CV4 7AL, Coventry, UK

[3] Department of Physics, University of Warwick, Gibbet Hill Road, CV4 7AL, Coventry, UK

*Corresponding Authors: tom.whale@warwick.ac.uk and m.i.gibson@warwick.ac.uk

Table of Contents

Materials and Methods S2

Materials........ S2

Characterization Techniques........ S2

Experimental Procedures........ S4

Synthetic Procedures S7

Synthesis of *exo*-Norbornene imide *N*-Boc S7

Synthesis of 2-(ethoxycarbonothioylthio)-2-methylpropanoic acid NHS-ester S8

Photo-polymerization of vinyl acetate using 2-(ethoxycarbonothioylthio)-2-methylpropanoic acid NHS-ester........ S11

Synthesis of poly(amino *exo*-norbornene imide), P(NB-NH_2) homopolymer precursors *via* ring-opening metathesis polymerization (ROMP)........ S12

Synthesis of P((P(NB-NH)-*g*-PVA_{210})-*stat*-P(NB-NH_2))$_n$ graft copolymers *via* "*grafting-to*" approach........ S13

Synthesis of *exo*-5-norbornene-2-methylamine........ S14

Synthesis of 2-(ethoxycarbonothioylthio)-2-methylpropanoic acid *exo*-5-norbornene-2-methylamide (NB-CTA) S19

Photo-polymerization of vinyl acetate using NB-CTA........ S20

Synthesis of PNB_n-*g*-PVA_{208} bottlebrush polymers *via* ROMP "*grafting-through*" approach S21

Polymerization of poly(ethylene glycol) methyl ether methacrylate using 4-cyano-4-(phenylcarbonothioylthio)pentanoic acid S22

Supplementary Characterization Data for P((P(NB-NH)-*g*-PVA_{210})-*stat*-P(NB-NH_2))$_n$ Graft Copolymers........ S23

Supplementary Characterization Data for PNB_n-*g*-PVA_{208} Bottlebrush Polymers S29

Supplementary Characterization Data for P((P(NB-NH)-*g*-PEG_{227})-*stat*-P(NB-NH_2))$_{200}$ and P($PEGMA_{20}$)$_{200}$ Control Samples S33

Supplementary Ice Shaping/Nucleation Data........ S35

References........ S42

Materials and Methods

Materials

All chemicals were used as supplied unless otherwise stated. Catalyst G3 ((H_2IMes)(pyr)$_2$(Cl)$_2$Ru=CHPh), cis-5-norbornene-*exo*-2,3-dicarboxylic anhydride (95%), *exo*-5-norbornene carboxylic acid (97%), *N*-Boc-ethylenediamine (≥98%), 1-hydroxypyrrolidine-2,5-dione (98%), ethyl vinyl ether (EVE) (≥99%), trifluoroacetic acid (TFA, 99%), mPEG$_{10K}$-succinimidyl carboxymethyl ester (NHS-mPEG$_{227}$, M_n ~ 10,000 Da), poly(ethylene glycol) methyl ether methacrylate (PEGMA$_{20}$, M_n ~ 1000 Da), 4-cyano-4-(phenylcarbonothioylthio)pentanoic acid (97%), 4,4'-azobis(4-cyanovaleric acid) (ACVA, ≥98%), poly(vinyl alcohol) (M_n ~ 146,000-186,000 Da, ≥99% hydrolyzed), hydrazine hydrate (reagent grade, 50-60 %), lithium aluminum hydride solution ($LiAlH_4$, 1.0 M in THF), 1-hydroxybenzotriazole hydrate (HOBt, ≥97%), triethylamine (TEA, ≥99%), PBS (tablets) and sucrose (≥99.5%) were purchased from Sigma Aldrich. The monomer vinyl acetate (VAc, ≥99%) was also purchased from Sigma Aldrich and passed through a column of basic alumina to remove inhibitor prior to use. Potassium ethyl xanthate (98%) was obtained from Alfa Aesar. *N*-(3-dimethylaminopropyl)-*N′*-ethylcarbodiimide hydrochloride (EDC.HCI, >98%) was purchased from Carbosynth. 2-bromo-2-methyl-propionic acid (≥98%) and lanolin (anhydrous, USP) was purchased from Acros Organics. Paraffin oil (technical grade) was purchased from VWR International. Solvents of toluene (anhydrous, ≥99.8%), dichloromethane (DCM, anhydrous, ≥99.8%), *N*,*N*-dimethylformamide (DMF, anhydrous, ≥99.8%), tetrahydrofuran (THF, anhydrous, ≥99.9%) were also purchased from Sigma Aldrich. Dialysis membranes (MWCO = 3.5/ 300 kDa) were purchased from Spectra/Por. Formvar-carbon coated (300 mesh) copper grids were purchased from EM Resolutions. Photo-polymerization reactions of vinyl acetate were conducted using an EvoluChem™ PhotoRedOx Temperature Controlled Box fitted with an EvoluChem™ LED spotlight (P201-18-2 450-455 nm) with total irradiance of 30 mW.cm^{-2} and light beam angle of 25° operating at a wavelength of λ = 450–455 nm. Ultra-pure water used for buffers was MilliQ grade (18.2 mΩ resistance).

Characterization techniques

NMR Spectroscopy. ^{1}H-NMR and ^{13}C-NMR spectra were recorded at 300 MHz or 400 MHz on a Bruker DPX-300 or DPX-400 spectrometer respectively, with chloroform-*d* ($CDCl_3$), methanol-d_4 (CD_3OD) and DMSO-d_6 (($CD_3)_2SO$) as the solvent. Chemical shifts of protons are reported as δ in parts per million (ppm) and are relative to tetramethyl silane (TMS) at δ = 0 ppm when using $CDCl_3$ or solvent residual peak (CH_3OH, δ = 3.31 ppm/ DMSO δ = 2.50 ppm).

Size Exclusion Chromatography in DMF. Size exclusion chromatography (SEC) analysis of graft and bottlebrush polymers was performed on an Agilent Infinity 1260 MDS instrument equipped with differential refractive index (DRI), viscometry (VS), dual angle light scatter (LS) and variable

wavelength UV detectors. The system was equipped with 2 x PLgel Mixed D columns (300 x 7.5 mm) and a PLgel 10 µm guard column. The mobile phase used was DMF (HPLC grade) containing 0.1% LiBr at 50 ^{o}C at flow rate of 1.0 mL.min^{-1}. Poly(methyl methacrylate) (PMMA) standards (Agilent EasyVials) were used for calibration between 2,200,000 – 550 g.mol^{-1}. Analyte samples were filtered through a nylon membrane with 0.22 μm pore size before injection. Number average molecular weights (M_n), weight average molecular weights (M_w) and dispersities ($Đ_M$ = M_w/M_n) were determined by conventional calibration and universal calibration using Agilent GPC/SEC software.

Size exclusion chromatography (SEC) analysis of poly(vinyl acetate) (PVAc) and poly(norbornene) (PNB-NBoc) homopolymer precursors was performed on an Agilent Infinity II MDS instrument equipped with differential refractive index (DRI), viscometry (VS), dual angle light scatter (LS) and variable wavelength UV detectors. The system was equipped with 2 x PLgel Mixed D columns (300 x 7.5 mm) and a PLgel 5 µm guard column. The mobile phase used was DMF (HPLC grade) containing 5 mM NH_4BF_4 at 50 ^{o}C at flow rate of 1.0 mL.min^{-1}. Poly(methyl methacrylate) (PMMA) standards (Agilent EasyVials) were used for calibration between 955,000 – 550 g.mol^{-1}. Analyte samples were filtered through a nylon membrane with 0.22 μm pore size before injection. Number average molecular weights (M_n), weight average molecular weights (M_w) and dispersities ($Đ_M$ = M_w/M_n) were determined by conventional calibration and universal calibration using Agilent GPC/SEC software.

FTIR Spectroscopy. Fourier Transform-Infrared (FTIR) spectroscopy measurements were carried out using an Agilent Cary 630 FT-IR spectrometer, in the range of 650 to 4000 cm^{-1}.

Turbidimetry. Turbidimetric analysis was performed an Agilent Cary 60 UV-vis spectrophotometer equipped with a Peltier heating and cooling system. Aqueous solutions of PNB_n-g-PVA_{208} (n = 20, 40) bottlebrush polymer samples were prepared at 1 mg.mL^{-1} with changes in transmittance monitored at λ = 700 nm by heating each sample from 20 °C to 85 °C at a rate of 5 °C.min^{-1}. The inflection point of each thermal phase transition curve was used to determine the lower critical solution temperature (LCST) in each case.

Dynamic Light Scattering. Hydrodynamic diameters (D_h) and size distributions of bottlebrush polymer samples were determined by dynamic light scattering (DLS) using a Malvern Zetasizer Nano ZS with a 4 mW He-Ne 633 nm laser module operating at 25 ^{o}C. Measurements were carried out at an angle of 173° (back scattering), and results were analyzed using Malvern DTS 7.03 software. All determinations were repeated 5 times with at least 10 measurements recorded for each run. For heating ramp experiments, D_h and PD determination for aqueous BBP solutions at appropriate analysis concentration was performed by heating each sample from 20 °C to 85 °C. D_h values were calculated using the Stokes-Einstein equation where particles are assumed to be spherical, while for cylindrical particles DLS was primarily used to detect multiple populations and obtain dispersity information.

Transmission Electron Microscopy. Dry-state stained TEM imaging was performed on either a JEOL JEM-2100 or a JEOL JEM-2100Plus microscope operating at an acceleration voltage of 200 kV. All dry-state samples were diluted with MilliQ water and then deposited onto formvar-coated copper grids. After roughly 1 min, excess sample was blotted from the grid and the grid was stained with an aqueous 1 wt% uranyl acetate (UA) solution for 1 min prior to blotting, drying and microscopic analysis.

Atomic Force Microscopy. (AFM) imaging was performed on a Bruker Icon AFM microscope. Samples were drop cast from 1 mg.mL^{-1} aqueous solutions onto a silicon wafer. The substrate was dried under a gentle N_2(g) flow prior to imaging. The tips for the AFM analysis (Bruker ScanAsyst-air) were purchased from BRUKER, with resonance frequency in the range of 45-95 kHz and spring constant in the range of 0.2-0.8 N/m. Acquired images taken in peak force tapping mode and were analyzed using the Gwyddion software.

Experimental Procedures

Splat Ice Recrystallization Inhibition Assay. Splat cooling assays were performed as previously described by Tomczak *et al*.[1] Briefly, a 10 µL sample was dropped 1.40 m onto a chilled glass coverslip, resting on a thin aluminium block cooled to -78 °C placed on dry ice. Upon hitting the coverslip, a wafer with diameter of approximately 10 mm and thickness 10 µm was formed instantaneously. The glass coverslip was transferred onto the Linkam cryostage and held at -8 °C using liquid nitrogen for 30 minutes. Photographs were obtained using an Olympus CX 41 microscope with a UIS-2 20x/0.45/∞/0-2/FN22 lens and crossed polarisers (Olympus Ltd), equipped with a Canon DSLR 500D digital camera. Images were taken of the initial wafer (to ensure that a polycrystalline sample had been obtained) and again after 30 minutes. Image processing was conducted using ImageJ. In brief, the number of ice crystals in the field of view was measured for each photograph. The average (mean) of these three measurements was then calculated to find the mean grain area (MGS). The average value and error were compared to that of PBS solution, as appropriate, as a negative control. Bottlebrush polymer sample solutions were first prepared upon dissolution in PBS on the range of [sample] = 0.5 – 0.008 mg.mL^{-1}.

Modified Sucrose Sandwich Ice Shaping Assay. Briefly, 1 mg.mL^{-1} of nanoparticle samples were dispersed in 45 wt % sucrose solution and sandwiched between two glass coverslips and sealed with immersion oil. Samples were cooled to −50 °C on a Linkam Biological Cryostage BCS196 with T95-Linkpad system controller equipped with a LNP95-Liquid nitrogen cooling pump, using liquid nitrogen as the coolant (Linkam Scientific Instruments UK). The temperature was then increased to −8 °C and held for 1 h to anneal. The samples were then heated at 0.5 °C.min^{-1} until few ice crystals remained and then cooled at 0.05 °C.min^{-1} and the shape of ice crystals observed. Micrographs were obtained every

0.1 °C using an Olympus CX41 microscope equipped with a UIS-2 20x/0.45/∞/0−2/FN22 lens (Olympus Ltd.) and a Canon EOS 500D SLR digital. Image processing was conducted using ImageJ.

Ice Shaping using Nanoliter Osmometer. An Otago nanoliter osmometer (Otago Osmometers, Dunedin, New Zealand) was used to measure polymer ice shaping. Briefly, 20 nL droplets of 2 mg.mL^{-1} samples were suspended in type B immersion oil (Type B, cargille immersion oil) on a 6-well cooling plate using a microcapillary system.[2,3] The samples were rapidly frozen by cooling the osmometer to ~ −40 °C. A rapid temperature increase was then conducted until the melting point was reached, and then the temperature was gradually increased (0.01 °C.min^{-1}) until only one ice crystal remained. Just before the ice crystal melted, the temperature was decreased until a discernible growth of the ice crystal was observed. It was observed and photographed with an Olympus CX41 microscope equipped with a UIS 20x/0.45/*/0-2/FN22 lens (Olympus Ltd.) and a Canon EOS 1200D digital SLR camera.

Ice Nucleation Measurements. A microlitre scale droplet freezing assay was used to establish the ice nucleating effectiveness of the polymer nanoparticles and their precursors. Ice nucleation measurements were performed in essentially the manner previously described by Whale *et al.* Although using a different temperature control apparatus.[4] Where the apparatus described by Whale *et al.* uses a commercial Stirling engine driven cryocooler to control temperature of freezing droplets the apparatus employed here employs a custom-made 40 mm by 40 mm aluminium cold stage thermally bonded to a TEC1-12704 Peltier thermoelectric cooler with Arctic Cooling MX-4 thermal compound. The hot side of the Peltier is cooled using a recirculating chiller. A Meerstetter TEC-1091-PT100 Precision Peltier Controller drives the Peltier, allowing precise control of the temperature of the cold stage. The temperature of the cold stage is independently monitored using two Netshushin PT100 platinum resistance thermometers (NR-141-100S-2-1.0-10-2000PLi-A-3) embedded directly under the measurement area of the cold stage. The platinum resistance thermometers are read by a PicoTech PT-104 Platinum Resistance Data Logger. Temperature measurement uncertainty for the PT100s is ± 0.15 °C.

For the experiments reported here, 2 mg.ml^{-1} bottlebrush and linear polymer solutions were made up gravimetrically in MilliQ water. Arrays of around 40 one µl droplets of the nucleator solutions and MilliQ water were pipetted onto a 22 mm diameter silanized slide (Hampton Research HR3-231) using a Sartorius Picus® electronic micropipette. The slides holding droplets were then placed on a temperature-controlled stage and cooled at a rate of 2 °C.min^{-1}. A video camera was used to monitor droplet freezing. A custom LabView program was used to directly link temperature measurements to video frames, allowing freezing temperatures of droplets to be determined and droplet fraction frozen curves to be constructed. The confidence intervals in Figure 3A were generated using a simple Monte-Carlo simulation.[5] Droplet fraction frozen curves were divided temperature bins with a width of 0.5°C. The number of events in any bin is expected to follow a Poisson distribution on repeated testing so 5000

Poisson distributed random numbers were generated for each bin, using the observed number of freezing events as the expectation value for the bin. The bars in Figure 3A indicate the interval in faction frozen in which 90% of the generated numbers fall. In experiments of this type, 'pure' water freezes several degrees warmer than would be expected for homogeneous ice nucleation, which occurs in the absence of any heterogeneous ice nucleating particles. Purification to the point that homogeneous nucleation occurs is generally very challenging and the reported background is typical for the size of droplets used.[6]

Ice Nucleation Measurements by Differential Scanning Calorimetry (DSC). To assess the ice nucleation effectiveness of the various polymers in small droplets water-in-oil emulsions were frozen in a TA DSC 2500 instrument under nitrogen flow. The method employed is very similar to that described by Marcolli *et al.*[7] and employed in later studies such as that of Kumar *et al.*[8] The oil matrix used consisted of 10 wt% lanolin in paraffin oil. To make emulsions a custom-made homogenizer was used to disperse 10 wt% of water or 2 mg.ml^{-1} polymer solutions in oil matrix. This process resulted in emulsions of the type shown in Figure S38. The size distributions of the emulsions were analyzed using ImageJ. Figure S39 shows a typical size distribution of droplets in an emulsion. The droplet size distribution produced by this method peaked at around 5 µm and was very similar for all emulsions tested. To conduct an experiment 10 mg of emulsion was sealed in a DSC pan and placed in the DSC. To stabilize the emulsions they were first frozen by cooling from ambient temperature to -50 °C at a rate of 10 °C.min^{-1} then warmed again to 20 °C to melt the water content of the emulsion. For measurement, the emulsions were cooled at 10 °C.min^{-1} to -20 °C then at 1 °C.min^{-1} to -50 °C. The DSC curves reported were generated during the 1 °C.min^{-1} cooling ramp.

In Figure S39 is can be seen that the DSC curve generated for pure water in our experiments is very similar to that reported by Marcolli *et al.* for pure water for a similar emulsion. It also can be seen in Figure 4B of the main text that in our experiments a 2 mg.ml^{-1} solution of P(PEGMA)$_{200}$ produces an identical DSC curve to that of pure water. Marcolli *et al.* reported slightly higher freezing temperatures for emulsions with larger 10 µm droplets, as would be expected due to the volume dependence of homogeneous nucleation. Ogawa *et al.* used a similar technique to study freezing of PVA solutions. They used emulsions containing smaller droplets than those employed here or in Marcolli *et al.* leading to lower freezing temperatures however as can be seen in Figure S39 Ogawa *et al.* recorded a much broader freezing peak than the present study or Marcolli *et al.* This is likely due to the higher cooling rate, 5 °C.min^{-1}, employed by Ogawa *et al.* For reference, Figure S39 also includes DSC curves reported by Ogawa *et al.* for their PVA$_{22}$ sample at a concentration of 2.99 mol.kg^{-1} which is 0.66 mg.ml^{-1}, close to the 2 mg.ml^{-1} we used, and at 5.09 mol.kg^{-1}, close to 112 mg.ml^{-1}, the concentration of maximum increase in ice nucleation temperature seen by Ogawa *et al.* In total, these comparisons show that our technique produces results compatible with those of other similar methods. As discussed in the main text, the analysis of ice nucleation effectiveness of different polymers in the paper depends on evaluation of the shift of onset of freezing, as in Ogawa *et al.* and Kumar *et al.*

Synthetic Procedures

Synthesis of *exo*-norbornene imide *N*-Boc

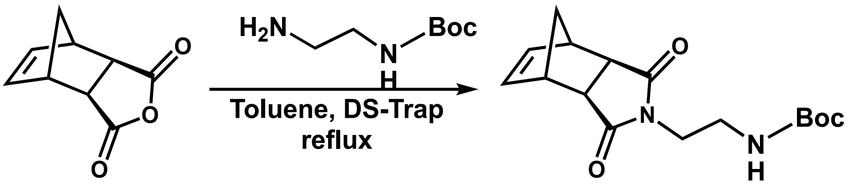

The synthesis of the *N*-Boc protected amino *exo*-norbornene imide was carried out according to a previously described process.[9,10] First, to a solution of cis-5-norbornene-*exo*-2,3-dicarboxylic anhydride (2.0 g, 12.1 mmol, 1 eq) in 100 mL of dry toluene was added *N*-Boc-ethylenediamine (2.34 g, 14.6 mmol, 1.2 eq) in 10 mL of dry toluene. The flask was fitted with a Dean-Stark trap and the reaction mixture was heated at reflux for 18 h. Upon cooling to room temperature, the reaction mixture was washed with 1 M HCl (3 × 100 mL) followed by sat. $NaHCO_3$ (1 × 100 mL). The organic layer was dried over $MgSO_4$, filtered, and concentrated to dryness under reduced pressure to afford a light brown solid as the pure product (2.62 g, 71%). ^{1}H-NMR (400 MHz, $CDCl_3$): δ (ppm) 6.29 (t, 2H, C***H***=C***H***), 4.83 (s, 1H, CH_2CH_2N***H***), 3.63 (t, 2H, CH_2C***H_2***NH), 3.33 (q, 2H, C***H_2***CH_2NH), 3.28 (p, 2H, C(O)C***H***C***H***C(O)), 2.70 (d, 2H, C***H***CH_2C***H***), 1.53 – 1.51 (d, 1H, CHC***H_2***CH), 1.41 (s, 9H, C(C***H_3***)$_3$), 1.27 – 1.24 (d, 1H, CHC***H_2***CH). ^{13}C-NMR (400 MHz, $CDCl_3$): δ (ppm) 178.2, 155.9, 137.8, 79.4, 47.9, 45.2, 42.9, 39.1, 38.5, 28.3. MS: m/z $[C_{16}H_{22}N_2O_4+Na]^+$ calc. 329.1 g.mol^{-1}, exp. 329.1 g.mol^{-1}.

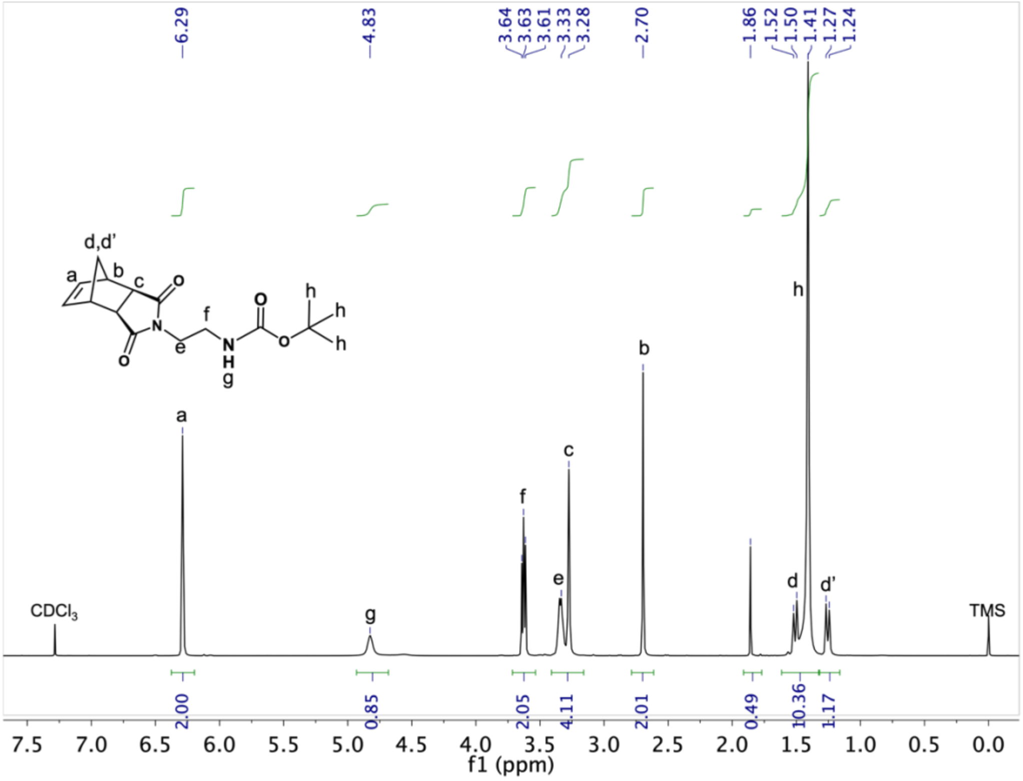

Figure S1. ^{1}H-NMR spectrum of *exo*-norbornene imide *N*-Boc in $CDCl_3$.

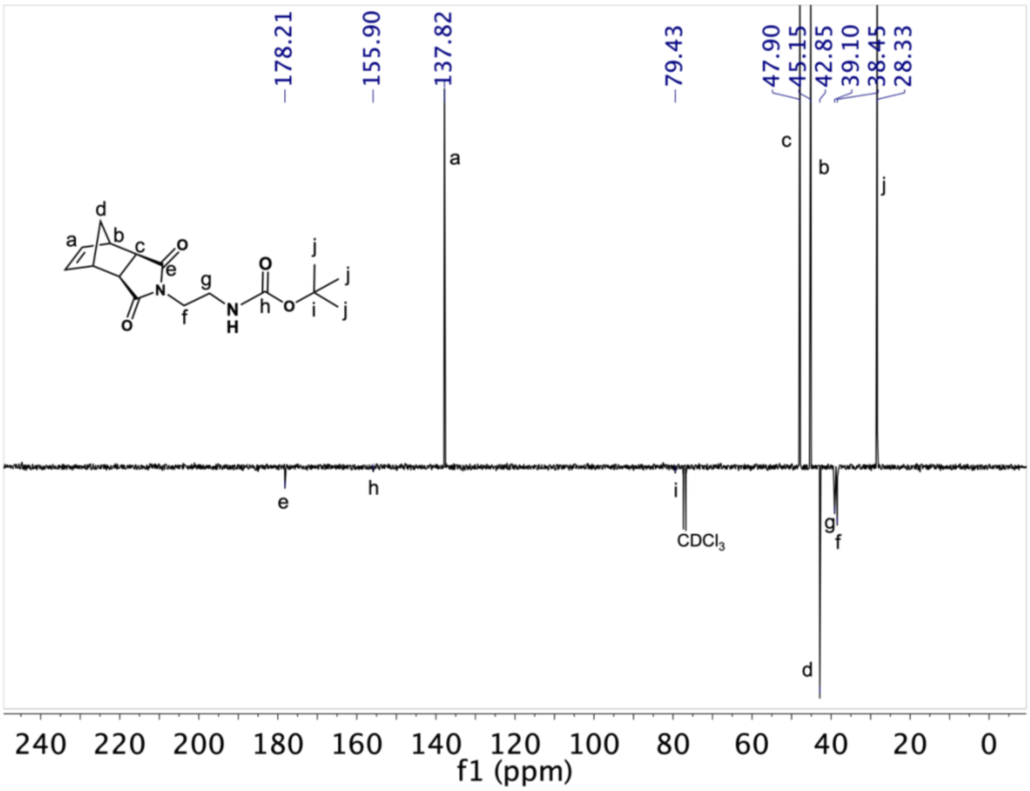

Figure S2. ^{13}C-NMR spectrum of *exo*-norbornene imide *N*-Boc in $CDCl_3$.

Synthesis of 2-(ethoxycarbonothioylthio)-2-methylpropanoic acid NHS-ester

(I) Synthesis of 2-(ethoxycarbonothioylthio)-2-methylpropanoic acid

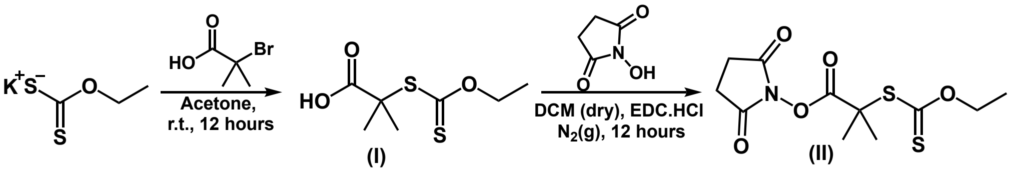

Briefly, acetone (100 mL) was placed into a round bottom flask equipped with a stirrer bar. Potassium ethyl xanthate (10.6 g, 65.9 mmol, 1.1 eq) was added and then stirred until dissolution. After 30 min, methyl 2-bromo-2-methyl-propionic acid (10.0 g, 59.9 mmol, 1 eq) was added and solution left to stir overnight at room temperature. The solution was then filtered to remove insoluble KBr, washed with cold acetone and then reduced under vacuum. The resulting residue was re-dissolved in 200 mL of DCM, washed with water (2 ×200 mL) and the aqueous layers combined and acidified with 6M HCl. The aqueous layers were extracted with DCM (3 ×200 mL) and combined with all organic layers. The solution was dried over $MgSO_4$, then filtered and concentrated *in vacuo*, affording a yellow oil. The crude product was recrystallized from hexane to afford the pure product as pale-yellow crystals (8.65 g, 69 % yield). ^{1}H-NMR (400 MHz, $CDCl_3$): δ (ppm) = 11.21 (br, 1H, COO***H***), 4.60 (q, 2H, $CH_3C\boldsymbol{H_2}O$), 1.65 (s, 6H, $SC(C\boldsymbol{H_3})_2CO$), 1.39 (t, 3H, $C\boldsymbol{H_3}CH_2O$). ^{13}C-NMR (400 MHz, $CDCl_3$): δ (ppm) = 210.4, 180.0, 70.0, 54.0, 25.6, 13.2. MS (ESI): m/z $[C_7H_{12}O_3S_2+Na]^+$calc. 231.1 g mol^{-1}, exp. 231.0 g mol^{-1}.

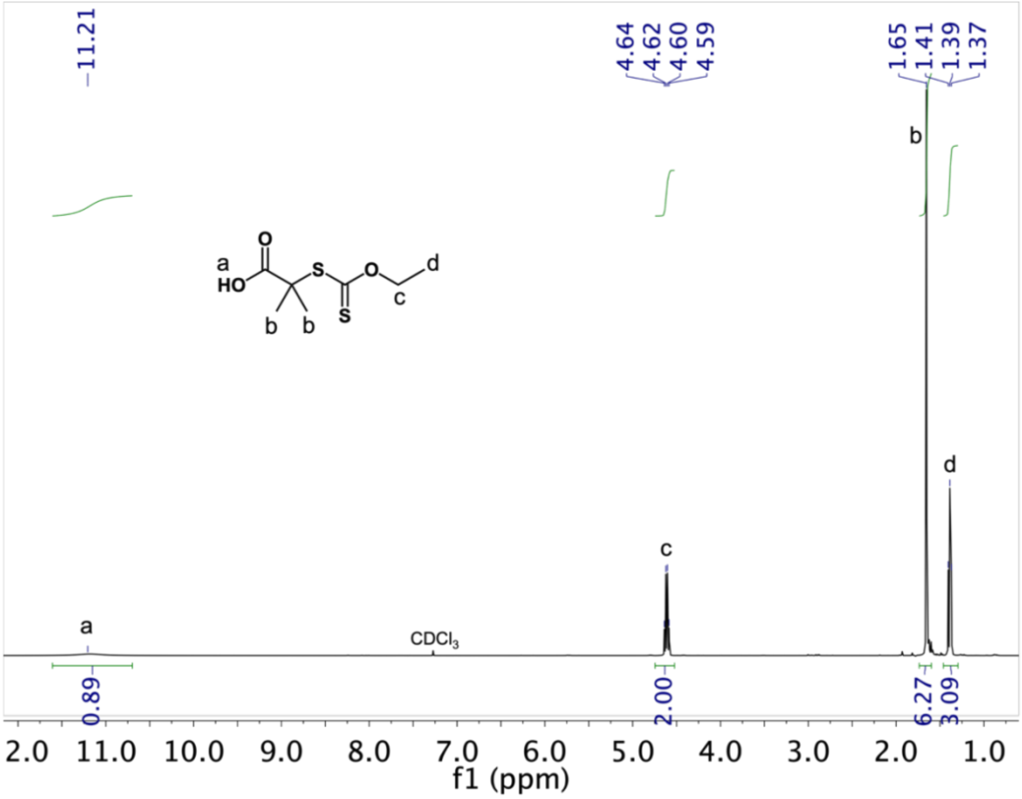

Figure S3. ^{1}H-NMR spectrum of 2-(ethoxycarbonothioylthio)-2-methylpropanoic acid in $CDCl_3$.

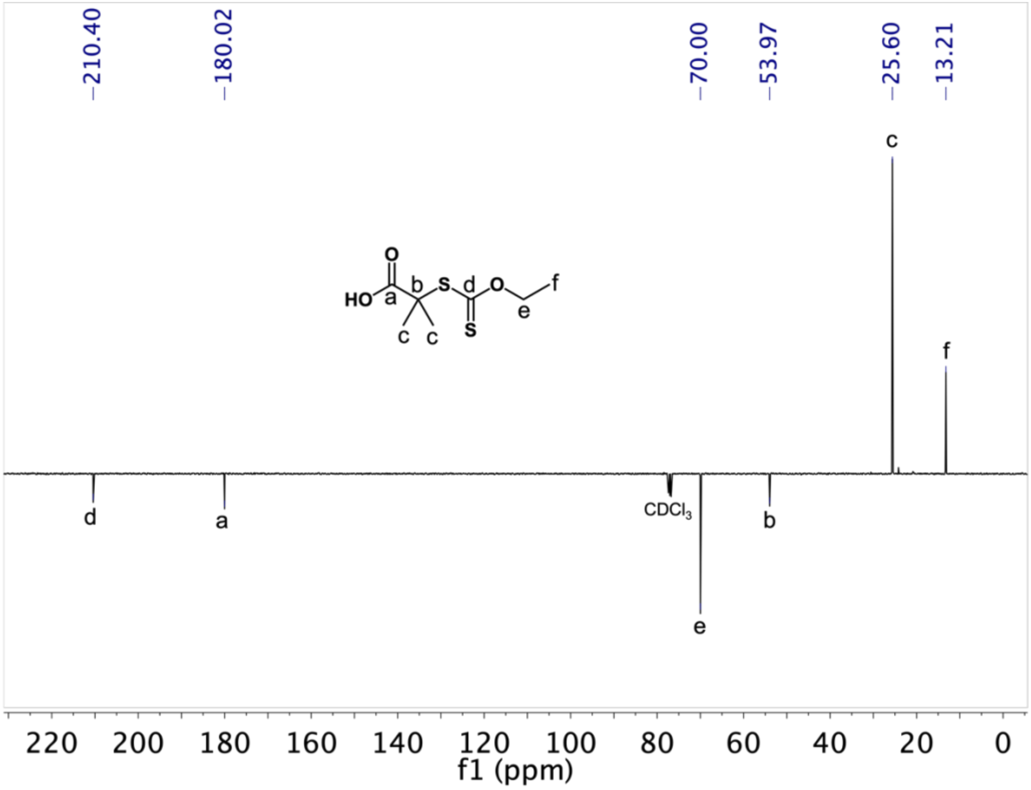

Figure S4. ^{13}C-NMR spectrum of 2-(ethoxycarbonothioylthio)-2-methylpropanoic acid in $CDCl_3$.

(II) Synthesis of 2-(ethoxycarbonothioylthio)-2-methylpropanoic acid NHS-ester

Next, 2-(ethoxycarbonothioylthio)-2-methylpropanoic acid (5.0 g, 24.0 mmol, 1 eq), EDC.HCl (5.8 g, 30.0 mmol, 1.25 eq) and 1-hydroxypyrrolidine-2,5-dione (3.5 g, 30.0 mmol, 1.25 eq) were dissolved and stirred in 100 mL of anhydrous DCM. The flask was put under positive nitrogen pressure and stirred for 48 hours. The solution was filtered under gravity and the filtrate solvent removed under vacuum. The crude solid was then dissolved in 100 mL diethyl ether and 100 mL saturated $NaHCO_3$ solution. The organic layer was washed with water (3× 100 mL), brine (1× 100 mL) and then dried with $MgSO_4$ and filtered under gravity. Solvent was then removed from the filtrate under vacuum. The crude product was further purified *via* flash column chromatography using silica gel eluting with hexane: ethyl acetate (8:2) to yield a yellow oil (4.78 g, 65% yield). ^{1}H-NMR (400 MHz, $CDCl_3$): δ (ppm) = 4.63 (q, 2H, $CH_3C\boldsymbol{H_2}O$), 2.76 (s, 4H, $C(O)C\boldsymbol{H_2}C\boldsymbol{H_2}C(O)$), 1.69 (s, 6H, $SC(C\boldsymbol{H_3})_2CO$), 1.30 (t, 3H, $C\boldsymbol{H_3}CH_2O$). ^{13}C-NMR (400 MHz, $CDCl_3$): δ (ppm) = 208.9, 169.3, 168.8, 70.9, 52.3, 26.0, 25.6, 13.0. MS (ESI): m/z $[C_7H_{12}O_3S_2+Na]^+$ calc. 328.1 g mol^{-1}, exp. 328.1 g mol^{-1}. FT-IR (neat): ν (cm^{-1}): 1780 (C=O), 1731 (C=O, NHS group), 1239 (C-O), 1042 (C=S).

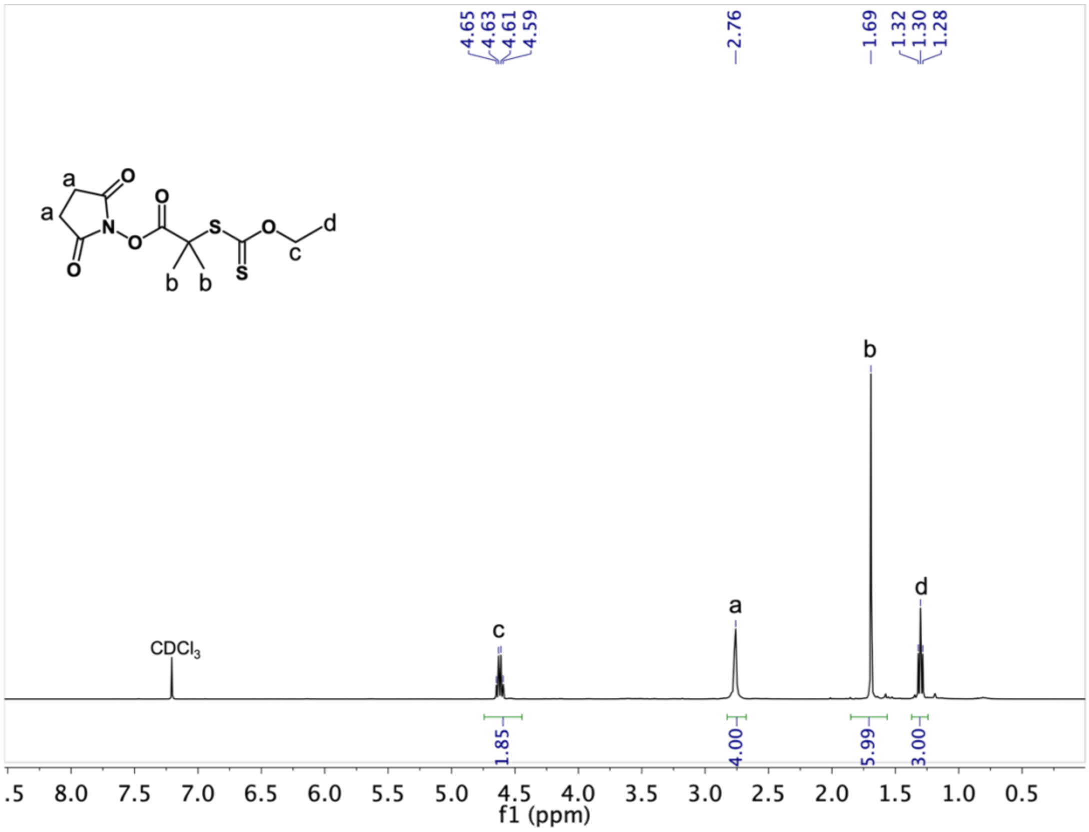

Figure S5. ^{1}H-NMR spectrum of 2-(ethoxycarbonothioylthio)-2-methylpropanoic acid NHS-ester in $CDCl_3$.

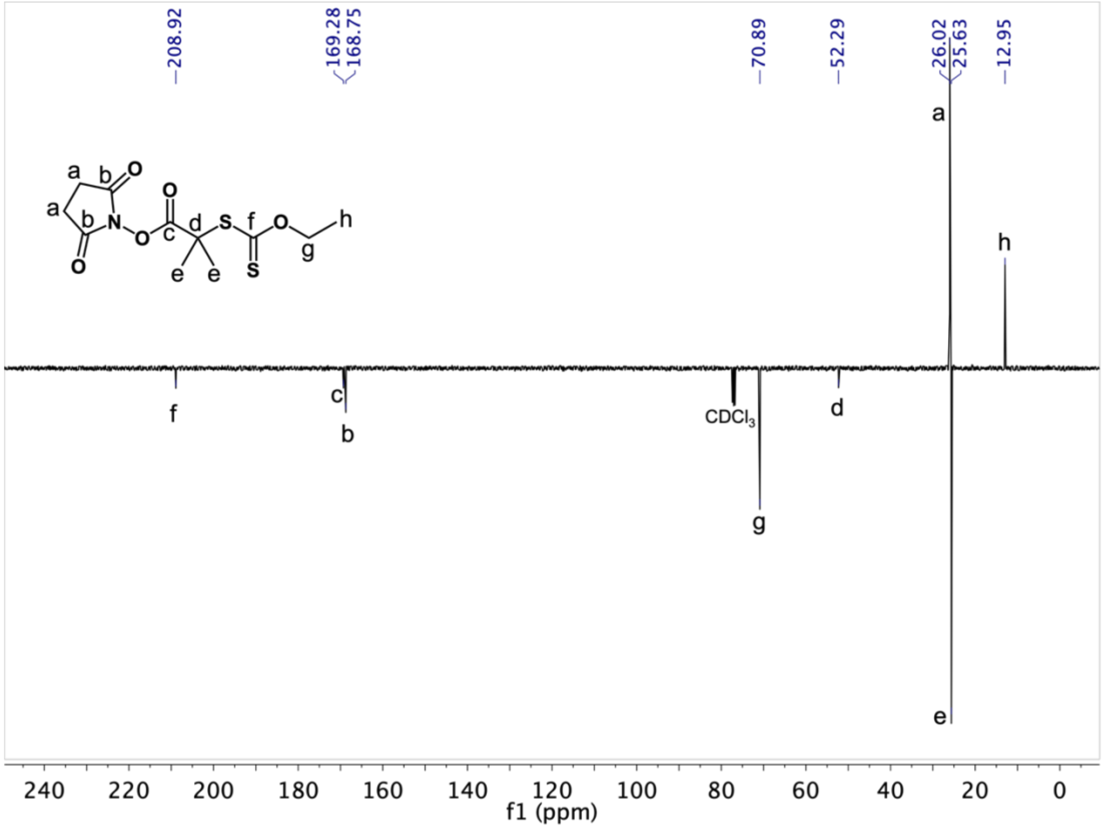

Figure S6. ^{13}C-NMR spectrum of 2-(ethoxycarbonothioylthio)-2-methylpropanoic acid NHS-ester in $CDCl_3$.

Photo-polymerization of vinyl acetate using 2-(ethoxycarbonothioylthio)-2-methylpropanoic acid NHS-ester.

2-(ethoxycarbonothioylthio)-2-methylpropanoic acid NHS-ester (0.10 g, 0.33 mmol, 1 eq) and vinyl aetate (VAc) (8.46 g (9.06 mL), 98.2 mmol, 300 eq) were dissolved in 2.1 mL of dioxane in a 20 mL vial (80% w/w solids content). Resulting solution was degassed by sparging with N_2(g) for 30 min and the sealed vial was incubated at 37°C with magnetic stirring under 460 nm light irradiation for 8h. After that time, polymerization was quenched by removing sealing and exposing it to air. An aliquot of crude polymerization mixture was withdrawn for ^{1}H-NMR in $CDCl_3$ for conversion and $M_{n,\ NMR}$ analysis. The reaction was rapidly cooled in liquid nitrogen and precipitated into diethyl ether. The polymer was re-precipitated into hexane from THF twice to yield a pale-yellow sticky polymer product that was further dried under vacuum. $M_{n,NMR}$ was calculated by end-group analysis by comparing the integrations of the –(C$\boldsymbol{H_2}$)$_2$ signals (s, 2.83 ppm) of NHS-group with those of the corresponding signals of the –C***H*** signal (d, 3.69-4.02 ppm) of polymer backbone. ^{1}H-NMR (400 MHz, $CDCl_3$): δ (ppm) = 5.05-4.76 5.05-4.76 (br s, 210H, CH_2C***H***O of polymer backcbone), 2.83 (s, 4H, C$\boldsymbol{H_2}$C$\boldsymbol{H_2}$ of NHS), 2.17-1.94 (br m, 630H, OC(O)C$\boldsymbol{H_3}$ of polymer side chain), 1.94-1.58 (br m, 420H, C$\boldsymbol{H_2}$CHO of polymer backbone), 1.34 (t, 3H, C$\boldsymbol{H_3}$$CH_2$O). Conversion = 69%, $M_{n,NMR}$ = 18400 g mol^{-1} (DP$_{PVAc,\ NMR}$ = 210). SEC (5 mM NH_4BF_4 in DMF) $M_{n,\ SEC\ RI}$= 22800 g mol^{-1}, $Đ_{M,\ SEC\ RI}$ = 1.6.

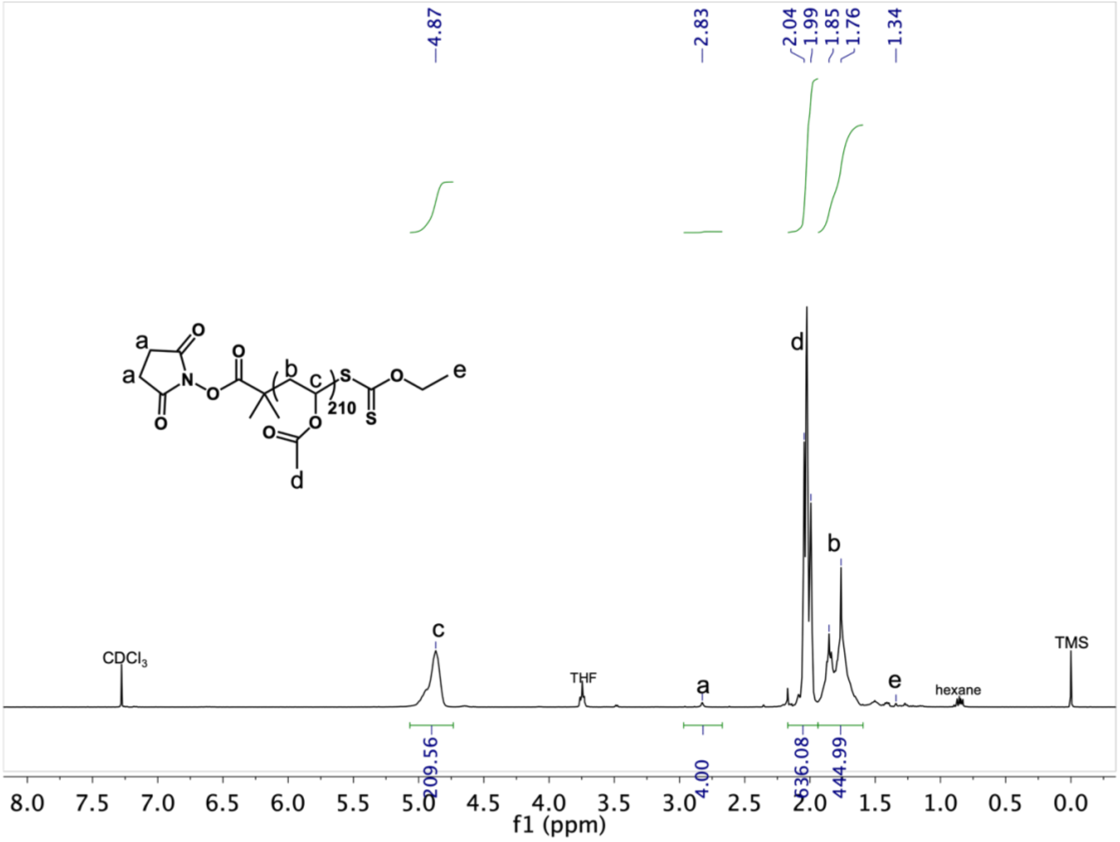

Figure S7. ^{1}H-NMR spectrum for NHS-end poly(vinyl acetate)$_{210}$ (NHS-PVAc$_{210}$) recorded in $CDCl_3$.

Synthesis of poly(amino *exo*-norbornene imide), P(NB-NH$_2$) homopolymer precursors *via* ring-opening metathesis polymerization (ROMP)

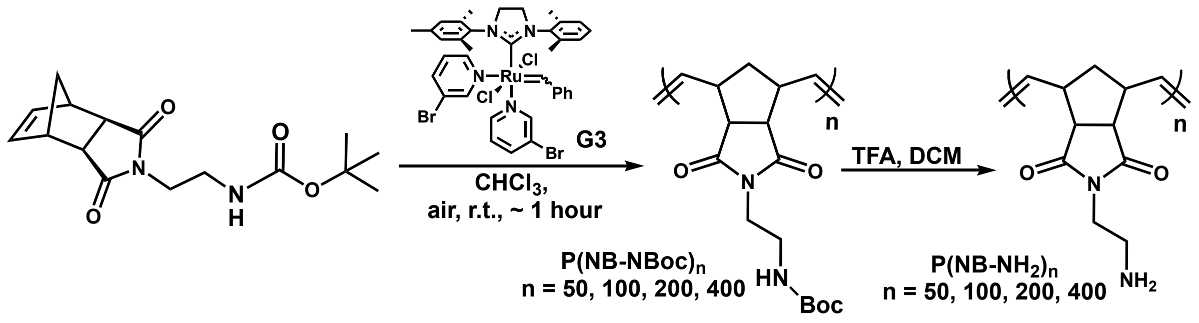

A typical procedure for the synthesis of P(NB-NBoc)$_{50}$ homopolymer *via* solution ROMP is described. A stock solution of 100 mg/mL of G3 in $CHCl_3$ and a solution of 173 mg of NB-NBoc (50 eq, 0.57 mmol) in 3.3 mL of $CHCl_3$ were first prepared. Then, 100 µL of G3 stock solution (10 mg, 1 eq, 0.011 mmol) were added to the vial of NB-NBoc monomer solution with rapid stirring and polymerization was allowed to proceed at room temperature for 1 h (final [NB-NBoc] = 50 mg/mL, final [G3] = 2.9 mg/mL). Polymerization was then quenched by addition of a few drops of ethyl vinyl ether and P(NB-NBoc)$_{50}$ homopolymer was precipitated from diethyl ether, isolated by vacuum filtration, and dried under vacuum prior to ^{1}H-NMR and SEC analyses. Procedure was repeated for [NB-NBoc]:[G3] ratios

of 100, 200 and 400. ^{1}H-NMR (400 MHz, $CDCl_3$) $M_{n,\ theo.}$ = 15,300 g.mol^{-1}. SEC (DMF + 5 mM NH_4BF_4) $M_{n,\ SEC}$ = 17,600 g.mol^{-1}, $Đ_{M,\ SEC}$ = 1.10.

Next, P(NB-NBoc)$_{50}$ homopolymer (100 mg, 0.003 mmol) was dissolved in a 1:1 TFA: DCM solution (5 mL) and stirred at room temperature for 4 hours. Polymer reaction mixture was concentrated to dryness under reduced pressure to afford a brown residue that was precipitated by the addition of diethyl ether. Removal of the solvent resulted to isolation of the amino deprotected polymer product as a brown solid (P(NB-NH$_2$)$_{50}$). Resulted polymer was then dissolved in DI water and dialyzed against DI water (dialysis membrane MWCO = 3.5 kDa) for approximately 14 h and lyophilized to afford the polymer products as brown solid. Procedure was repeated for P(NB-NBoc)$_{100,\ 200,\ 400}$ homopolymers. SEC characterization of the resulting deprotected poly(amino *exo*-norbornene imide) polymers was not possible due to high sample-column interactions. ^{1}H-NMR (400 MHz, DMSO-d_6): δ (ppm) = 8.04-7.92 (br s, $CH_2N\boldsymbol{H_2}$ of polymer side chain), 5.73-5.56 (br m, C***H***$CHCH_2$ of polymer backbone), 5.56-5.33 (br m, C***H***$CHCH_2$ of polymer backbone), 3.74-3.48 (br s, $CH_2C\boldsymbol{H_2}NH_2$ of polymer side chain), 3.48-3.24 (br m, $C\boldsymbol{H_2}CH_2NH_2$ of polymer side chain), 3.24-2.83 (br s, C(O)C***H***C***H*** C(O)of polymer backbone), 2.83-2.56 (br s, C***H***CH_2C***H*** of polymer backbone), 2.13-1.80 (br s, $CHC\boldsymbol{H_2}CH$ of polymer backbone), 1.62-1.32 (br s, $CHC\boldsymbol{H_2}CH$ of polymer backbone).

Synthesis of P((P(NB-NH)-*g*-PVA$_{210}$)-*stat*-P(NB-NH$_2$))$_n$ graft copolymers *via* "*grafting-to*" approach

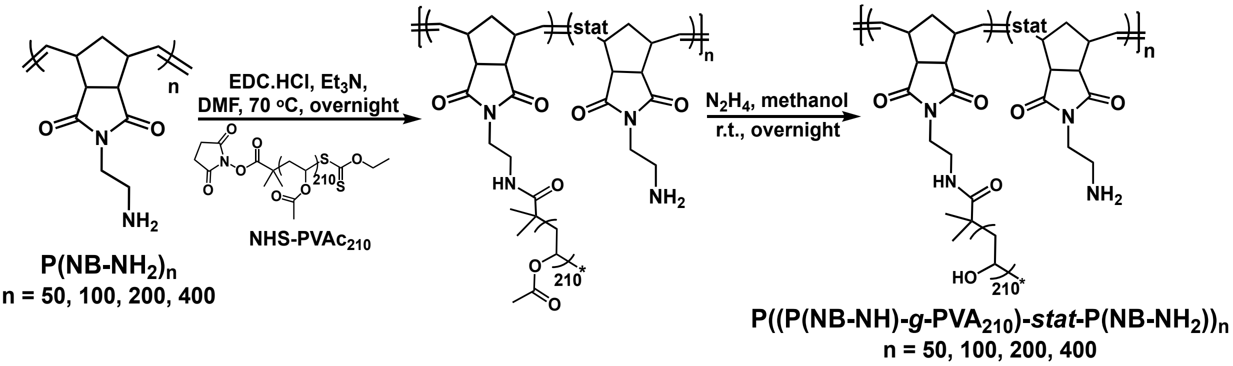

A representative synthesis of P((P(NB-NH)-*g*-PVA$_{210}$)-$_{stat}$-P(NB-NH$_2$))$_{50}$ by "*grafting-to*" is as follows: NHS-PVAc$_{210}$ (2.0 mg, 0.0002 mmol, 1 eq), P(NB-NH$_2$)$_{50}$ (0.34 mg, 0.018 mmol, 100 eq) and EDC.HCl (3.5 mg, 0.018 mmol, 100 eq) were dissolved in 6.5 mL of anhydrous DMF, following by the addition of Et_3N (2 μL, 0.018 mmol, 100 eq) and reaction mixture was stirred at 70 °C for 16 h. An aliquot of the crude reaction mixture was then taken for SEC analysis in DMF (+ 0.1% LiBr). Resulted polymer mixture was precipitated twice in diethyl ether. Resulted P(NB-NH$_2$)$_{50}$-*g*-PVAc$_{210}$ branched copolymer was then dissolved in a methanol (2 mL) following by the addition of and hydrazine hydrate solution (5 mL, 50-60 % in water) in a stoppered vial. The reaction mixture was stirred at room temperature overnight. Resulted graft copolymer was then dissolved in DI water and dialyzed against DI water

(dialysis membrane MWCO = 300 kDa) for approximately 2 weeks to ensure full removal of unfunctionalized PVA_{210} and lyophilized to afford the polymer product as spongy pale-yellow solid. Procedure was repeated for the synthesis of P((P(NB-NH)-*g*-PVA_{210})-*stat*-P(NB-NH_2))$_{100,200,400}$ and control sample P((P(NB-NH)-*g*-PEG_{227})-*stat*-P(NB-NH_2))$_{200}$. Complete hydrolysis was confirmed by ^{1}H-NMR analysis in DMSO-d_6. ^{1}H-NMR (400 MHz, DMSO-d_6): δ (ppm) = 4.74-4.63 (br s, $CH_2CHO\boldsymbol{H}$ of polymer side chain), 4.58-4.41 (br s, $CH_2CHO\boldsymbol{H}$ of polymer side chain), 4.32-4.18 (br m, $CH_2CHO\boldsymbol{H}$ of polymer side chain), 3.99-3.73 (br m, $CH_2C\boldsymbol{H}OH$ of polymer backbone), 1.71-1.14 (br m, $C\boldsymbol{H_2}CHOH$ of polymer backbone).

Synthesis of *exo*-5-norbornene-2-methylamine

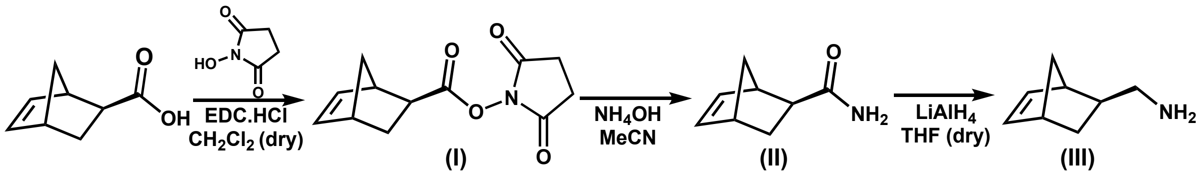

(I) *Synthesis of exo-5-norbornene NHS-ester*

Monomer of *exo*-5-norbornene NHS-ester was synthesized according to previously reported processes.[11–13] *Exo*-5-norbornene carboxylic acid (4.00 g, 29.0 mmol, 1 eq), EDC.HCl (6.94 g, 36.2 mmol, 1.25 eq) and 1-hydroxypyrrolidine-2,5-dione (4.16 g, 36.2 mmol, 1.25 eq) were stirred in anhydrous DCM (100 mL) under a nitrogen atmosphere overnight at room temperature. The mixture was diluted with water (100 mL) and then the aqueous mixture was extracted with DCM (2 × 50 mL). Afterwards, the combined organic phases were washed twice with saturated aq. $NaHCO_3$ (2 × 100 mL), followed by saturated aq. NH_4Cl (2 × 100 mL) and brine (100 mL). The organic phase was dried over $MgSO_4$ and the solvent was removed *in vacuo* to yield an off-white solid. The solid was recrystallized from ethanol to afford the pure product as white crystals (4.21 g, 62%). ^{1}H-NMR (400 MHz, $CDCl_3$): δ (ppm) 6.20 (m, 2H), 3.27 (s, 1H), 3.01 (s, 1H), 2.84 (d, 4H), 2.49 (m, 1H), 2.06 (m, 1H), 1.55-1.46 (m, 3H). ^{13}C-NMR (400 MHz, $CDCl_3$): δ (ppm) 171.7, 169.3, 138.6, 135.3, 47.1, 46.4, 41.8, 40.3, 31.0, 25.6.

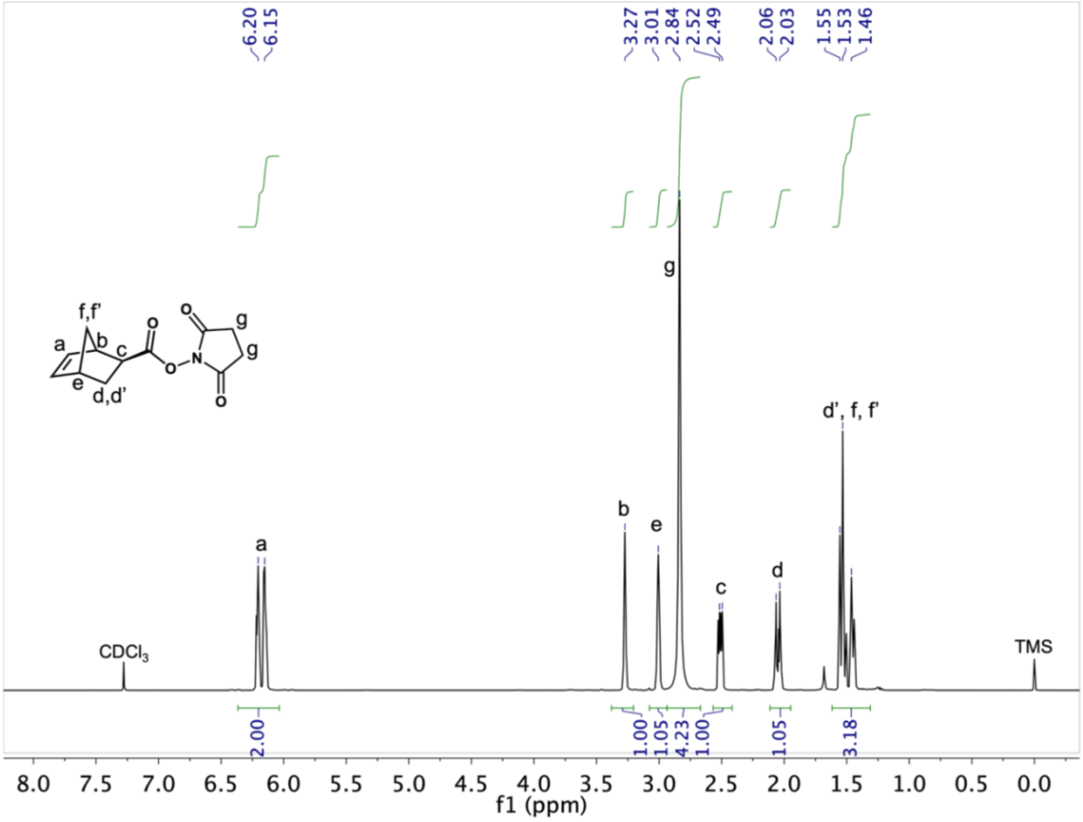

Figure S8. ^{1}H-NMR spectrum of *exo*-5-norbornene NHS-ester in $CDCl_3$.

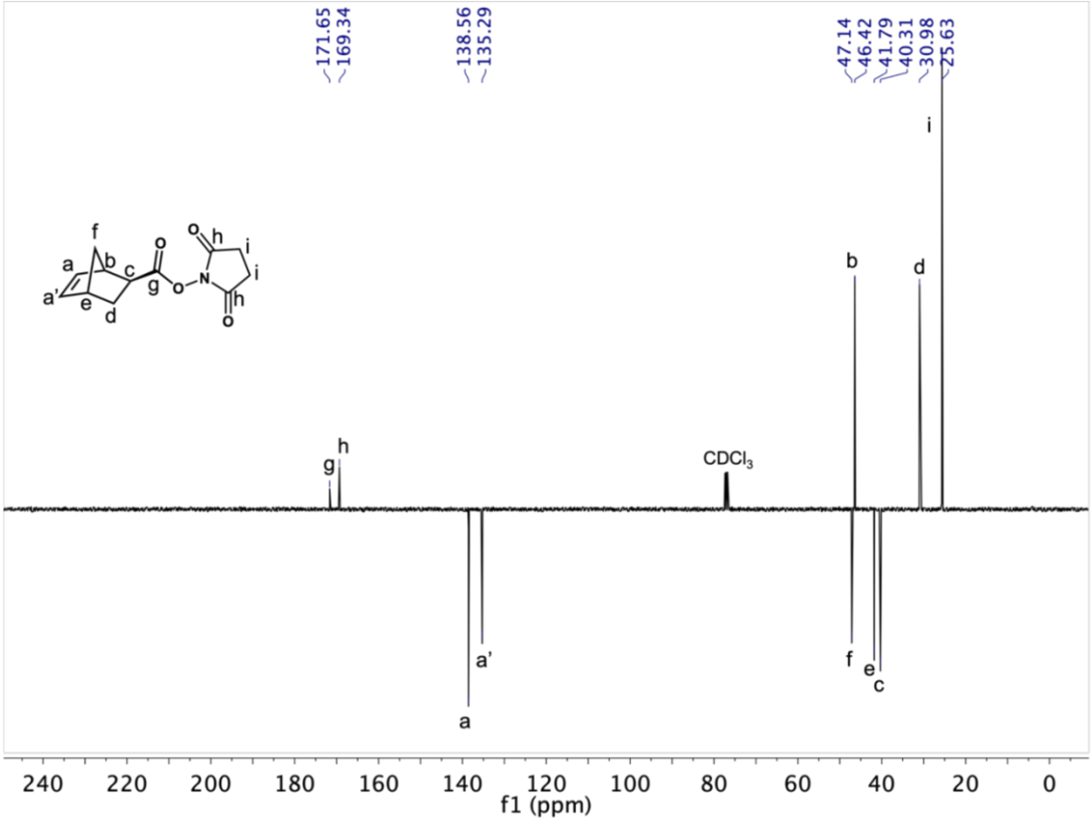

Figure S9. ^{13}C-NMR spectrum of *exo*-5-norbornene NHS-ester in $CDCl_3$.

(II) Synthesis of *exo*-5-norbornenecarboxamide

Monomer of *exo*-5-norbornenecarboxamide was synthesized according to previously reported process.[12–14] Briefly, *exo*-5-norbornene NHS-ester (4.0 g, 17 mmol, 1 eq) was dissolved in 50 mL of MeCN in a round bottom flask. Then, 160 mL of NH_4OH solution (28% in H_2O) were added to the flask. The reaction mixture was stirred for 30 min at room temperature. The reaction mixture was then diluted with 200 mL of DI H_2O. The flask was placed in an ice bath, and the solution was acidified to pH ~ 7 using concentrated HCl solution. The aqueous solution was then extracted with DCM (2 × 200 mL), and the combined organic layers were dried over $MgSO_4$ and concentrated under reduced pressure to afford the pure product as a white powder (2.04 g, 88 %). ^{1}H-NMR (400 MHz, $CDCl_3$): δ (ppm) 6.15 (m, 2H), 5.55 (s, 2H), 2.99 (s, 1H), 2.93 (s, 1H), 2.11 (m, 1H), 1.95 (m, 1H), 1.67 (d, 1H), 1.37 (m, 2H). ^{13}C-NMR (400 MHz, CDCl3): δ (ppm) 178.4, 138.3, 135.9, 47.1, 46.4, 44.1, 41.6, 30.6.

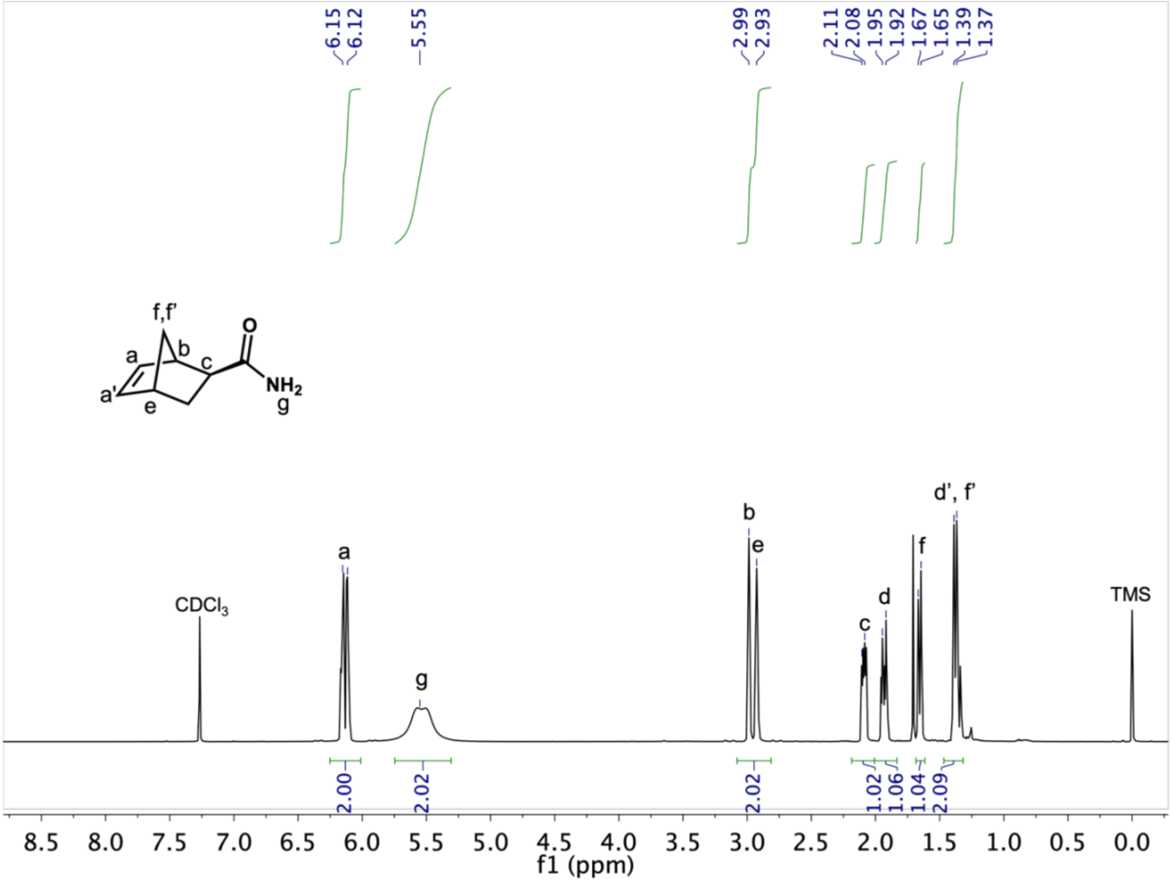

Figure S10. ^{1}H-NMR spectrum of *exo*-5-norbornenecarboxamide in $CDCl_3$.

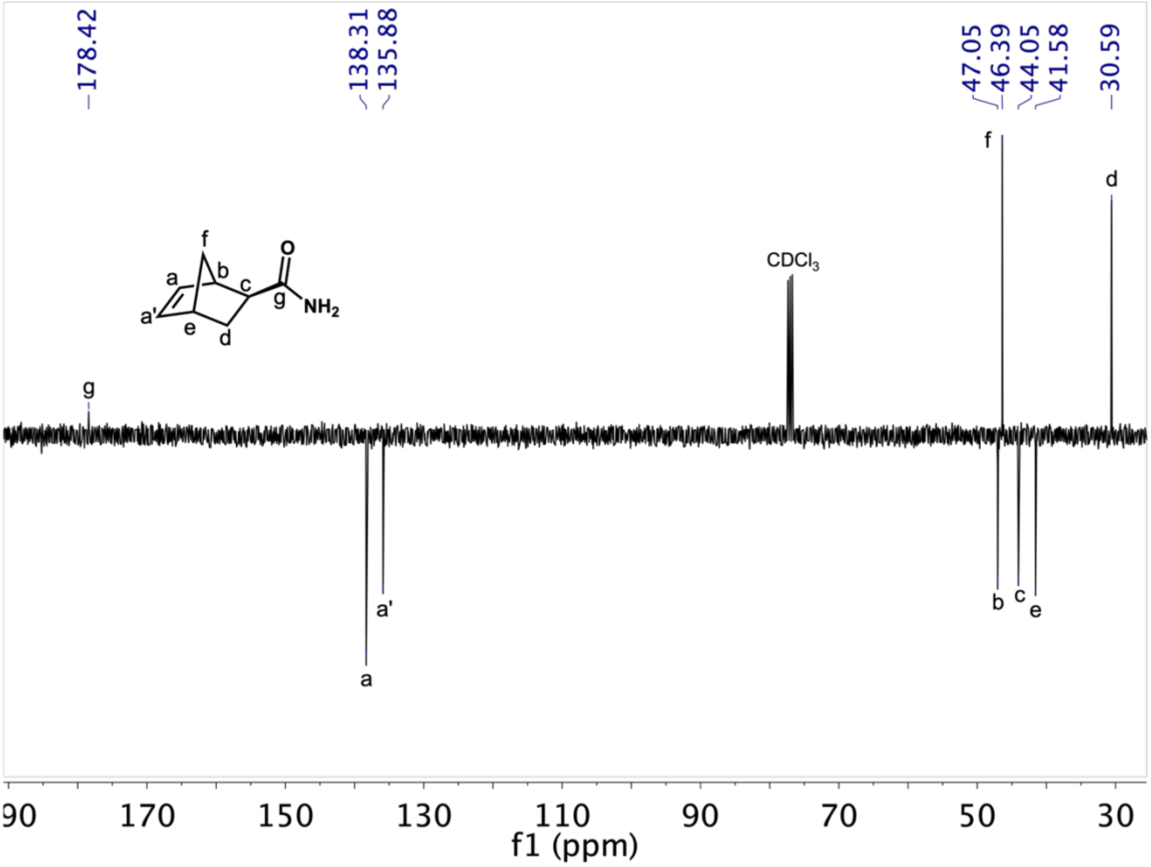

Figure S11. ^{13}C-NMR spectrum of *exo*-5-norbornenecarboxamide in $CDCl_3$.

(III) Synthesis of *exo*-5-norbornene-2-methylamine

Monomer of *exo*-5-norbornene-2-methylamine was synthesized according to previously reported process.[12] *Exo*-5-norbornenecarboxamide (2 g, 14.6 mmol, 1.0 eq) was dissolved in 50 mL of anhydrous THF. A lithium aluminum hydride solution (1.0M in THF, 22 mL, 0.83 g, 21.9 mmol, 1.5 eq) was added dropwise over 5 minutes, and the reaction was heated at reflux for 48 hours. After cooling in an ice water bath, the reaction was quenched by dropwise addition of aqueous 1 M NaOH until there was no more gas evolution upon addition, as determined visually. The aqueous solution was then extracted with DCM (2 × 100 mL), and the combined organic layers were dried over $MgSO_4$ and concentrated under reduced pressure to afford desired amino-norbornene product as a clear, pale yellow, viscous oil (1.37 g, 76%). ^{1}H-NMR (400 MHz, $CDCl_3$): δ (ppm) 6.09 (m, 2H), 2.80 (s, 1H), 2.71 (s, 1H), 2.69 (m, 1H), 2.64 (s, 1H), 1.43 (m, 1H), 1.35-1.25 (m, 5H), 1.12 (m, 1H). ^{13}C-NMR (400 MHz, $CDCl_3$): δ (ppm) 136.5, 47.8, 45.0, 44.0, 43.0, 41.6, 31.0. MS: m/z $[C_8H_{13}N+H]^+$ calc. 124.1 g mol^{-1}, exp. 124.1 g mol^{-1}.

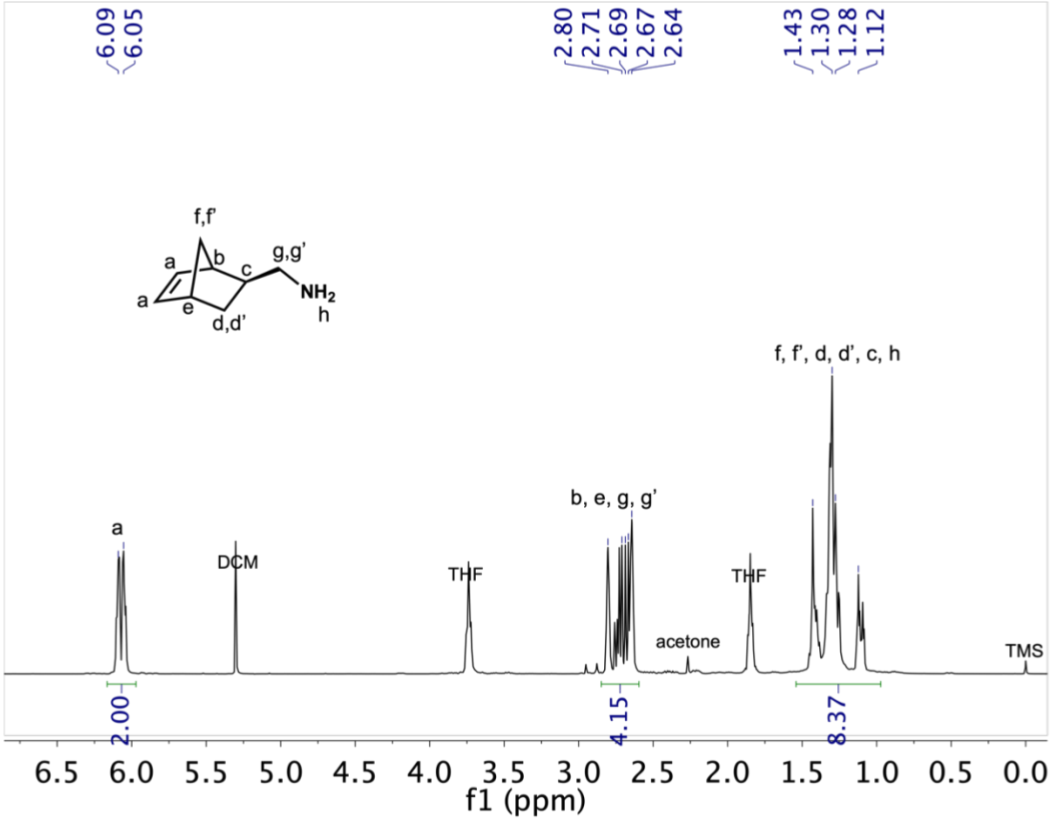

Figure S12. ^{1}H-NMR spectrum of *exo*-5-norbornene-2-methylamine in $CDCl_3$.

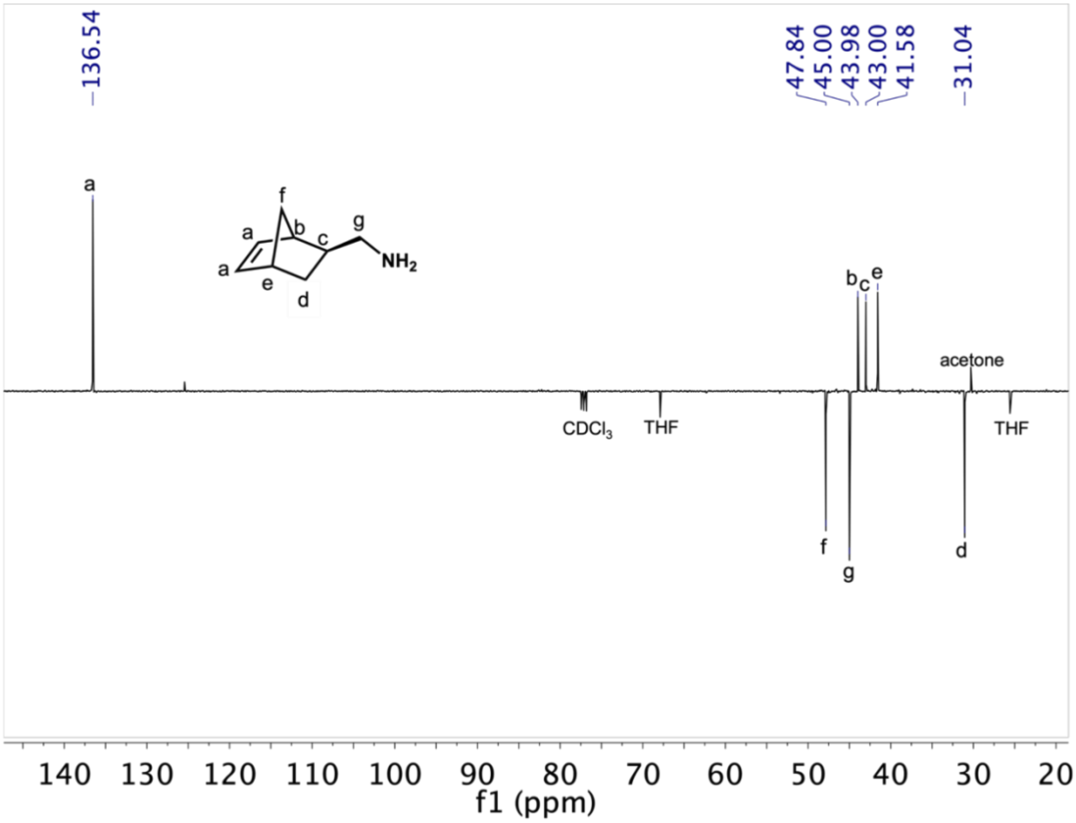

Figure S13. ^{13}C-NMR spectrum of *exo*-5-norbornene-2-methylamine in $CDCl_3$.

Synthesis of 2-(ethoxycarbonothioylthio)-2-methylpropanoic acid *exo*-5-norbornene-2-methylamide (NB-CTA)

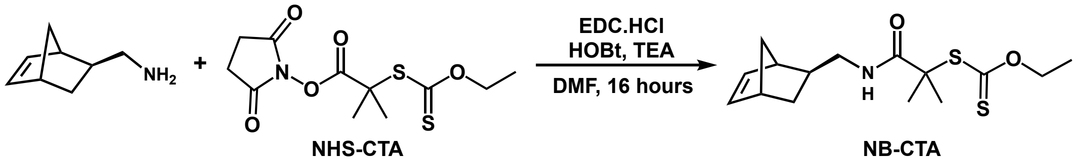

A 50 mL flask equipped with a magnetic stirring bar was charged with 2-(ethoxycarbonothioylthio)-2-methylpropanoic acid NHS-ester (0.5 g, 1.63 mmol, 1.0 eq), EDC·HCl (0.37 g, 1.96 mmol, 1.2 eq), HOBt (0.29 g, 1.96 mmol, 1.2 eq), and 20 mL of anhydrous DMF. The mixture was stirred until the solids dissolved completely (~ 10 min) and solution was degassed under N_2(g) for 30 min. To the flask was added *exo*-5-norbornene-2-methylamine (0.20 g, 1.63 mmol, 1.0 eq) followed by TEA (272 µL, 1.96 mmol, 1.2 eq). The reaction mixture was stirred at room temperature for 16 h. The solvent was removed in vacuo and the crude residue was purified by silica gel chromatography (2:8 EtOAc/hexanes) to afford the pure product, NB-CTA, as a yellow oil (0.37 g, 73% yield). ^{1}H NMR (400 MHz, $CDCl_3$) δ (ppm): 6.54 (s, 1H) ,6.07 (s, 2H), 4.62 (m, 2H), 3.30 (m, 1H), 3.3.22 (m, 1H), 2.83 (s, 1H), 2.55 (s, 1H), 1.61 (m, 6H), 1.42 (m, 1H), 1.40 (t, 3H), 1.38 (m, 1H), 1.25 (m, 1H), 1.14 (d, 2H). ^{13}C-NMR (400 MHz, $CDCl_3$) δ (ppm): 209.9, 172.7, 136.9, 136.1, 70.2, 55.6, 45.4, 45.0, 44.2, 41.7, 39.1, 30.8, 26.3, 13.4. MS (ESI): m/z $[C_{15}H_{23}NO_2S_2+Na]^+$ calc. 336.1 g mol^{-1}, exp. 336.1 g mol^{-1}. FT-IR (neat): ν (cm^{-1}): 1645 (C=C), 1239 (C-O), 1042 (C=S).

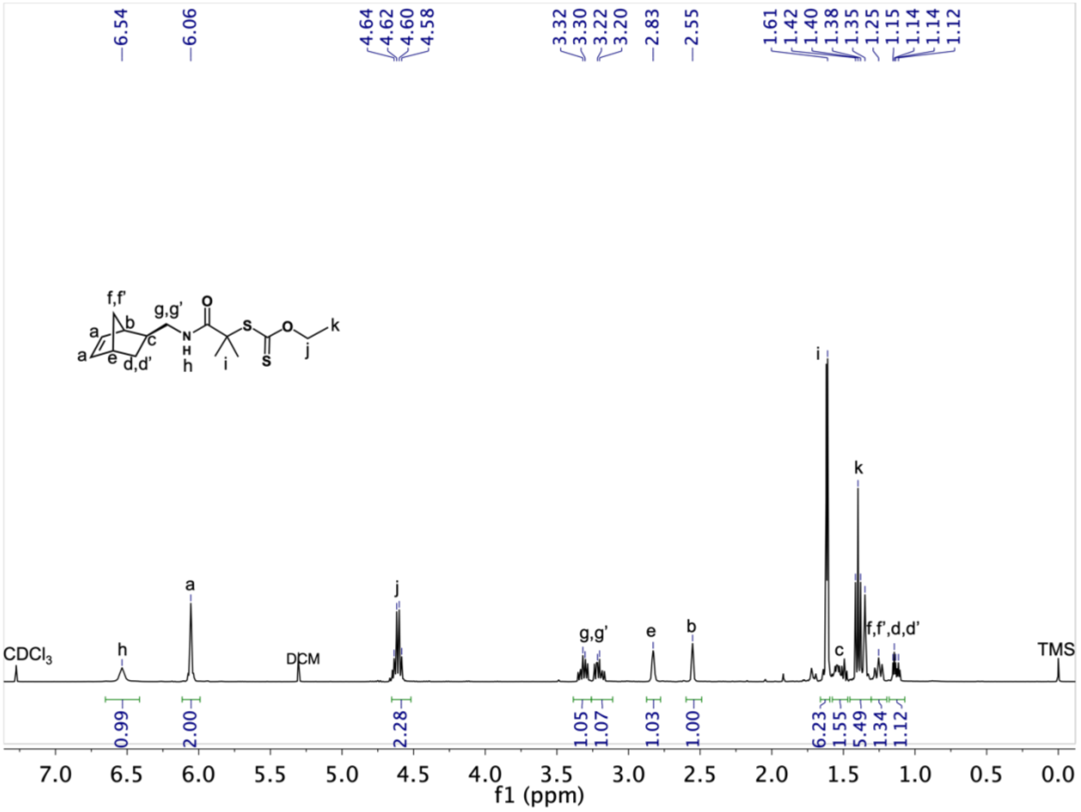

Figure S14. ^{1}H-NMR spectrum of 2-(ethoxycarbonothioylthio)-2-methylpropanoic acid *exo*-5-norbornene-2-methylamide (NB-CTA) in $CDCl_3$.

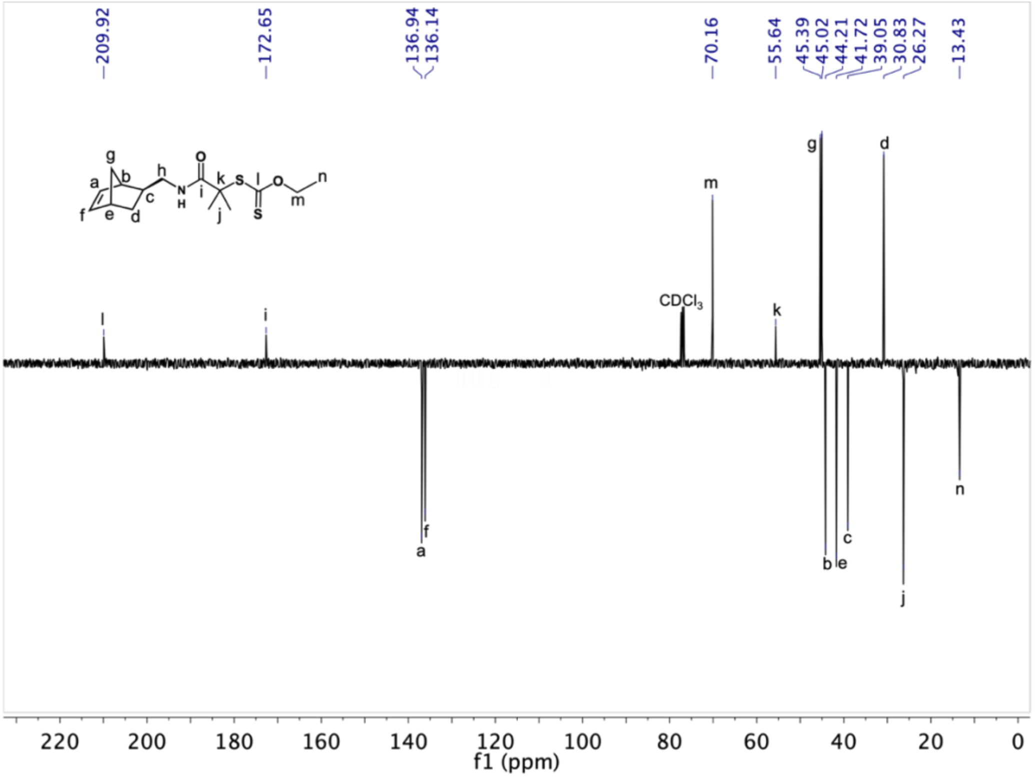

Figure S15. ^{13}C-NMR spectrum of 2-(ethoxycarbonothioylthio)-2-methylpropanoic acid *exo*-5-norbornene-2-methylamide (NB-CTA) in $CDCl_3$.

Photo-polymerization of vinyl acetate using NB-CTA.

2-(ethoxycarbonothioylthio)-2-methylpropanoic acid exo-5-norbornene-2-methylamide (0.10 g, 0.32 mmol, 1 eq) and vinyl aetate (VAc) (8.24 g (8.82 mL), 95.7 mmol, 300 eq) were dissolved in 2.1 mL of dioxane in a 20 mL vial (80% w/w solids content). Resulting solution was degassed by sparging with N_2(g) for 30 min and the sealed vial was incubated at 37°C with magnetic stirring under 460 nm light irradiation for 8h. After that time, polymerization was quenched by removing sealing and exposing it to air. An aliquot of crude polymerization mixture was withdrawn for ^{1}H NMR in $CDCl_3$ for conversion and $M_{n,NMR}$ analysis. The reaction was rapidly cooled in liquid nitrogen and precipitated into diethyl ether. The polymer was re-precipitated into hexane from THF twice to yield a pale-yellow sticky polymer product that was further dried under vacuum. $M_{n,NMR}$ was calculated by end-group analysis by comparing the integrations of the –(C***H***C***H***) signals (s, 6.28 ppm) of norbornene-group with those of the corresponding signals of the –C***H*** signal (d, 3.69-4.02 ppm) of polymer backbone. ^{1}H NMR (400 MHz, $CDCl_3$): δ (ppm) = 6.28 (s, 2H, C***H***C***H*** of norbornene-end group), 5.05-4.76 (br s, 208H, $CH_2C\boldsymbol{H}O$ of polymer backcbone), 2.83 (s, 4H, $C\boldsymbol{H_2}C\boldsymbol{H_2}$ of NHS), 2.17-1.94 (br m, 630H, $OC(O)C\boldsymbol{H_3}$ of polymer side chain), 1.94-1.58 (br m, 420H, $C\boldsymbol{H_2}CHO$ of polymer backbone), 1.34 (t, 3H, $C\boldsymbol{H_3}CH_2O$). Conversion = 67%, $M_{n,NMR}$ = 18400 g mol^{-1} ($DP_{PVAc,\ NMR}$ = 208). SEC (5 mM NH_4BF_4 in DMF) $M_{n,\ SEC\ RI}$ = 17900 g mol^{-1}, $Đ_{M,\ SEC\ RI}$ = 1.40.

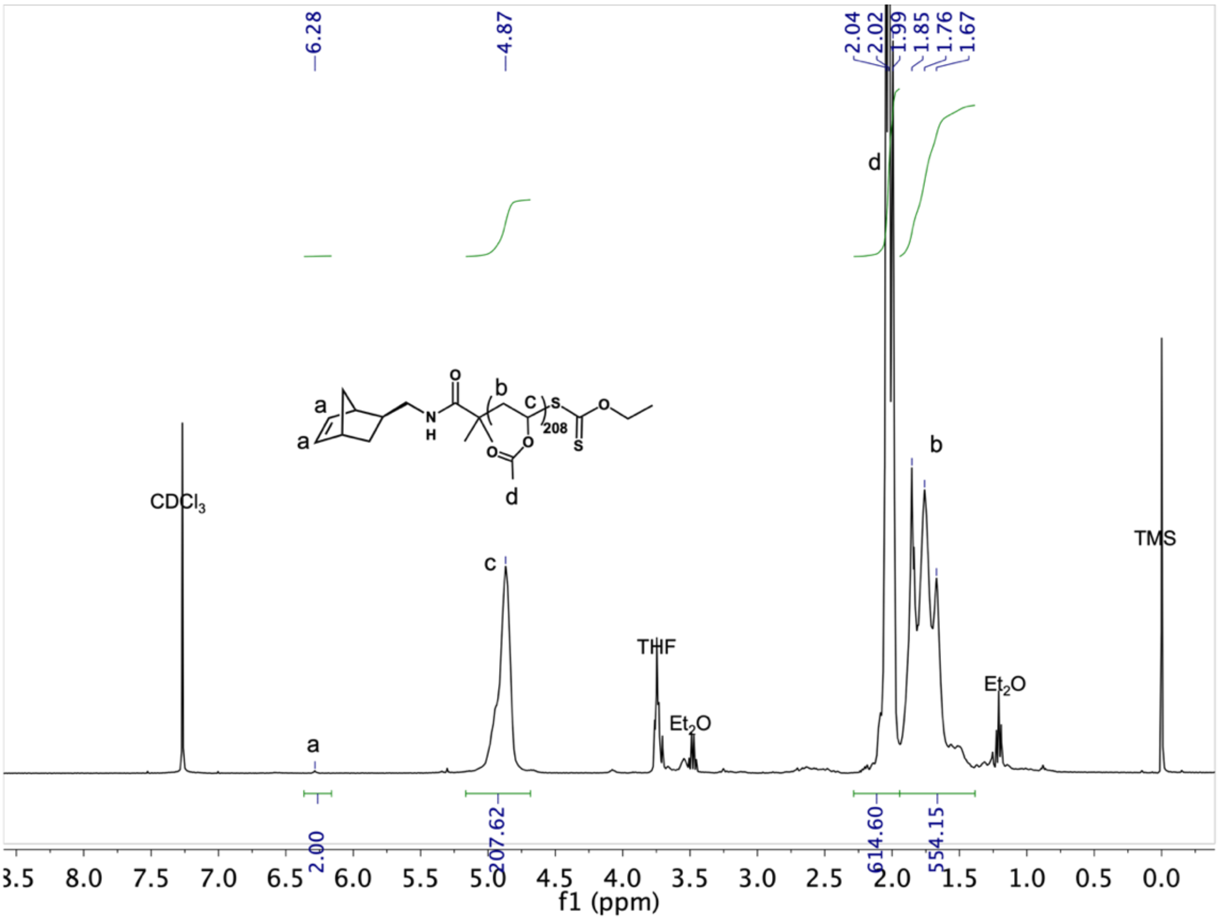

Figure S16. ^{1}H-NMR spectrum for *exo*-norbornene-end poly(vinyl acetate)$_{208}$, NB-PVAc$_{208}$ recorded in $CDCl_3$.

Synthesis of PNB$_n$-*g*-PVA$_{208}$ bottlebrush polymers *via* ROMP "*grafting-through*" approach

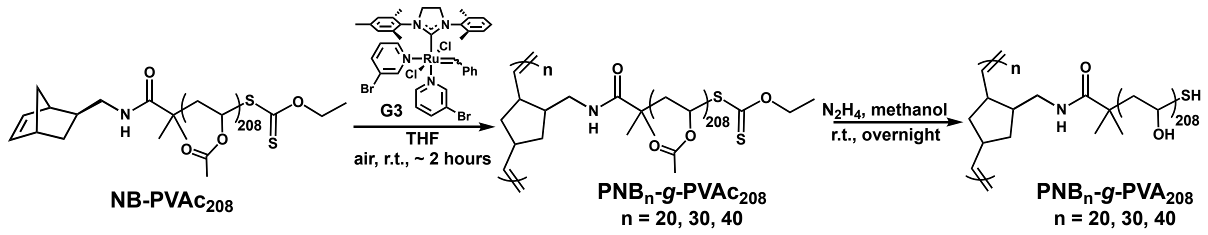

A representative synthesis of a PNB$_{20}$-*g*-PVAc$_{208}$ bottlebrush polymer by ROMP "*grafting-through*" is as follows: NB-PVAc$_{208}$ (100 mg, 0.006 mmol, 20 equiv) was dissolved in 1.9 mL of anhydrous THF in a 3 mL vial equipped with a magnetic stirring bar. To the vial was added 100 µL of a 2.0 mg.mL^{-1} stock solution of G3 in THF (final [NB-PVAc$_{208}$]/[G3] = 20, [NB-PVAc$_{208}$] = 50 mg.mL^{-1}). The reaction mixture was stirred at room temperature for 2 h. The polymerization was then quenched *via* addition of a few drops of ethyl vinyl ether. An aliquot of the crude reaction mixture was then taken for SEC analysis in DMF (+ 0.1% LiBr). The resulting polymer, PNB$_{20}$-*g*-PVAc$_{208}$, was isolated by precipitation from diethyl ether and was dried under vacuum.

Resulted PNB_{20}-*g*-$PVAc_{210}$ (50 mg) bottlebrush polymer was then dissolved in a methanol (0.5 mL) following by the addition of and hydrazine hydrate solution (3 mL, 50-60 % in water) in a stoppered vial. The reaction mixture was stirred at room temperature overnight. Resulted polymer was then dissolved in DI water and dialyzed against DI water (dialysis membrane MWCO = 300 kDa) for approximately 1 week to ensure full removal of unfunctionalized PVA_{208} and lyophilized to afford the polymer products as a white solid. Procedure was repeated for the synthesis of PNB_n-*g*-$PVAc_{210}$ (n = 30, 40). Complete hydrolysis was confirmed by ^{1}H-NMR analysis in DMSO-d_6. ^{1}H-NMR (400 MHz, DMSO-d_6): δ (ppm) = 4.74-4.63 (br s, $CH_2CHO\boldsymbol{H}$ of polymer side chain), 4.58-4.41 (br s, $CH_2CHO\boldsymbol{H}$ of polymer side chain), 4.32-4.18 (br m, $CH_2CHO\boldsymbol{H}$ of polymer side chain), 3.99-3.73 (br m, $CH_2C\boldsymbol{H}OH$ of polymer backbone), 1.71-1.14 (br m, $C\boldsymbol{H_2}CHOH$ of polymer backbone).

Polymerization of poly(ethylene glycol) methyl ether methacrylate using 4-cyano-4-(phenylcarbonothioylthio)pentanoic acid

A vial was charged with 4-cyano-4-(phenylcarbonothioylthio)pentanoic acid (5.0 mg, 0.018 mmol, 1 eq), $PEGMA_{20}$ (3.58 g, 3.6 mmol, 200 eq), ACVA (1.0 mg, 0.004 mmol, 0.2 eq), and 17 mL of DMF. The vial was then sealed and deoxygenated using three successive cycles of freeze-pump-thaw to remove O_2(g). The vial was placed into an aluminium heating block which had been pre-heated to 70 °C to initiate polymerization. After 24 h, the polymerization was quenched by exposing the vial to air and submerging it into liquid N_2. An aliquot was withdrawn for determination of monomer conversion by ^{1}H NMR spectroscopy. The polymer was precipitated into diethyl ether from dioxane twice to yield a white polymer product that was further dried under vacuum. ^{1}H NMR (400 MHz, methanol-d_4): δ (ppm) = 3.88-3.51 (br s, 16000H, $OC\boldsymbol{H_2}C\boldsymbol{H_2}$ of polymer side chain), 2.48-1.58 (br m, 400H, $C(CH_3)C\boldsymbol{H_2}$ of polymer backbone), 1.41-0.76 (br m, 600H, $C(C\boldsymbol{H_3})CH_2$ of polymer backbone), $M_{n,\, theo}$ = 200,000 g mol^{-1}. SEC (5 mM NH_4BF_4 in DMF) $M_{n,\, SEC\, RI}$ = 134,300 g mol^{-1}, $Đ_{M,\, SEC\, RI}$ = 2.0.

Supplementary Characterization Data for P((P(NB-NH)-*g*-PVA$_{210}$)-*stat*-P(NB-NH$_2$))$_n$ Graft Copolymers

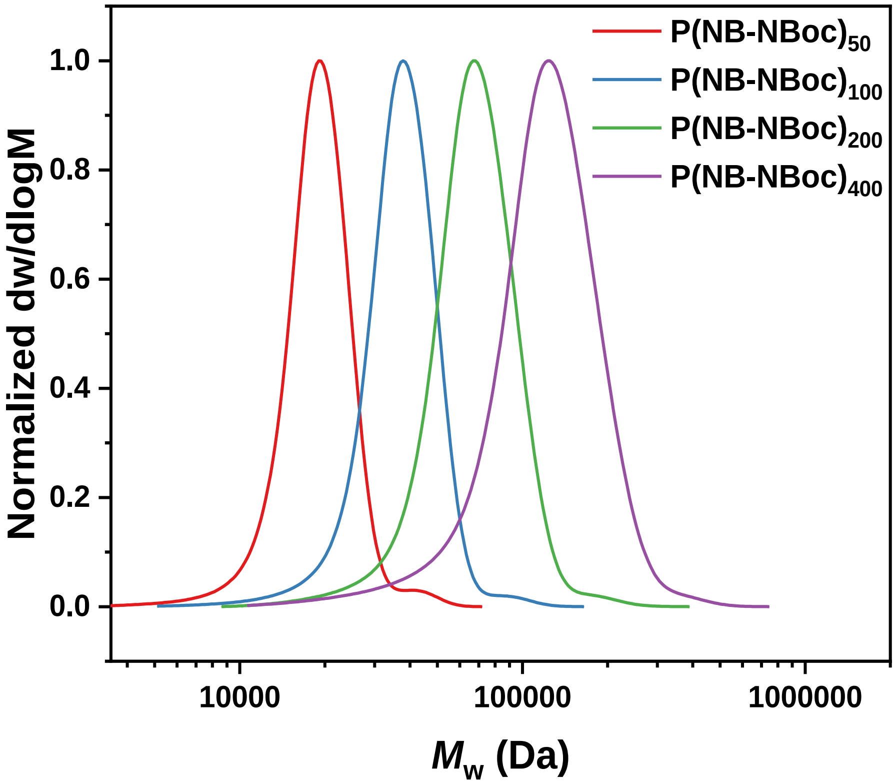

Figure S17. Normalized SEC RI molecular weight distributions for P(NB-NBoc)$_n$ (n = 50, 100, 200, 400) homopolymers using DMF + 5mM NH_4BF_4 as the eluent.

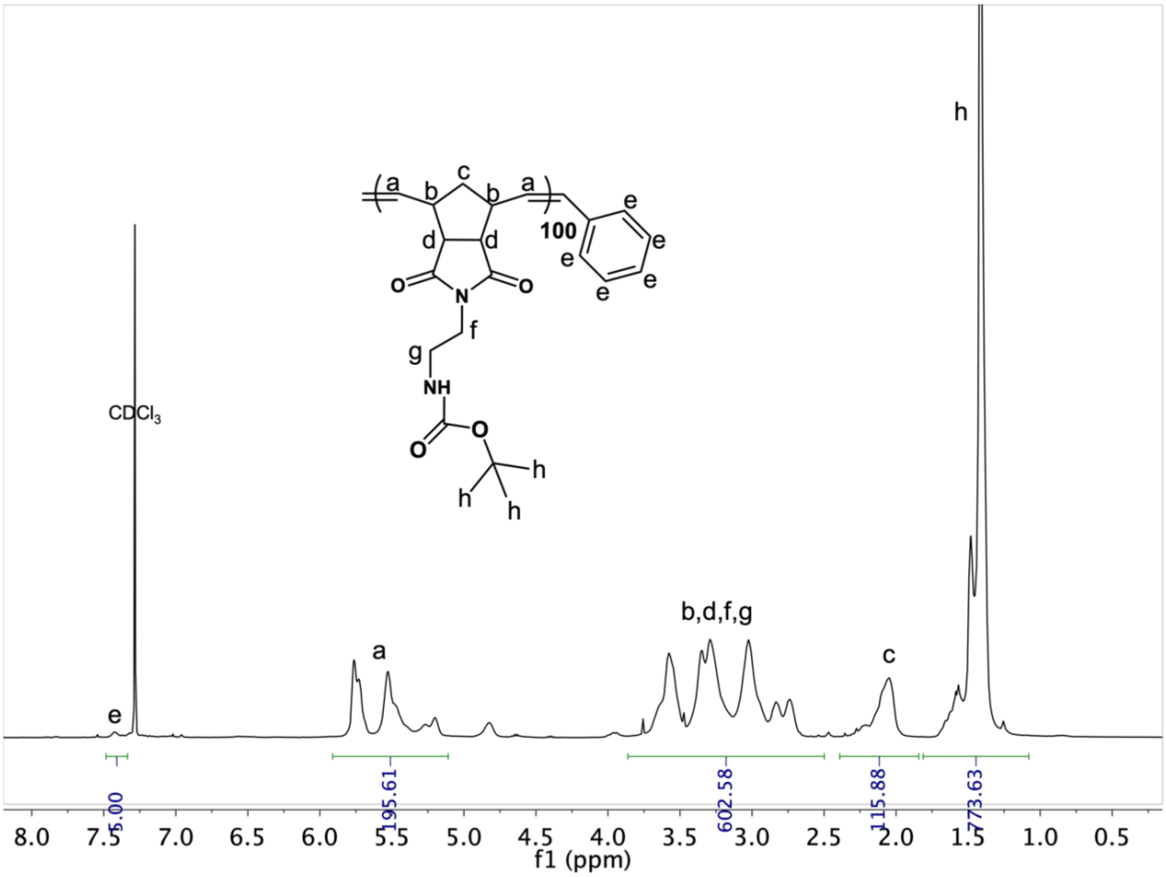

Figure S18. ^{1}H-NMR spectrum of P(NB-NBoc)$_{100}$ homopolymer in $CDCl_3$.

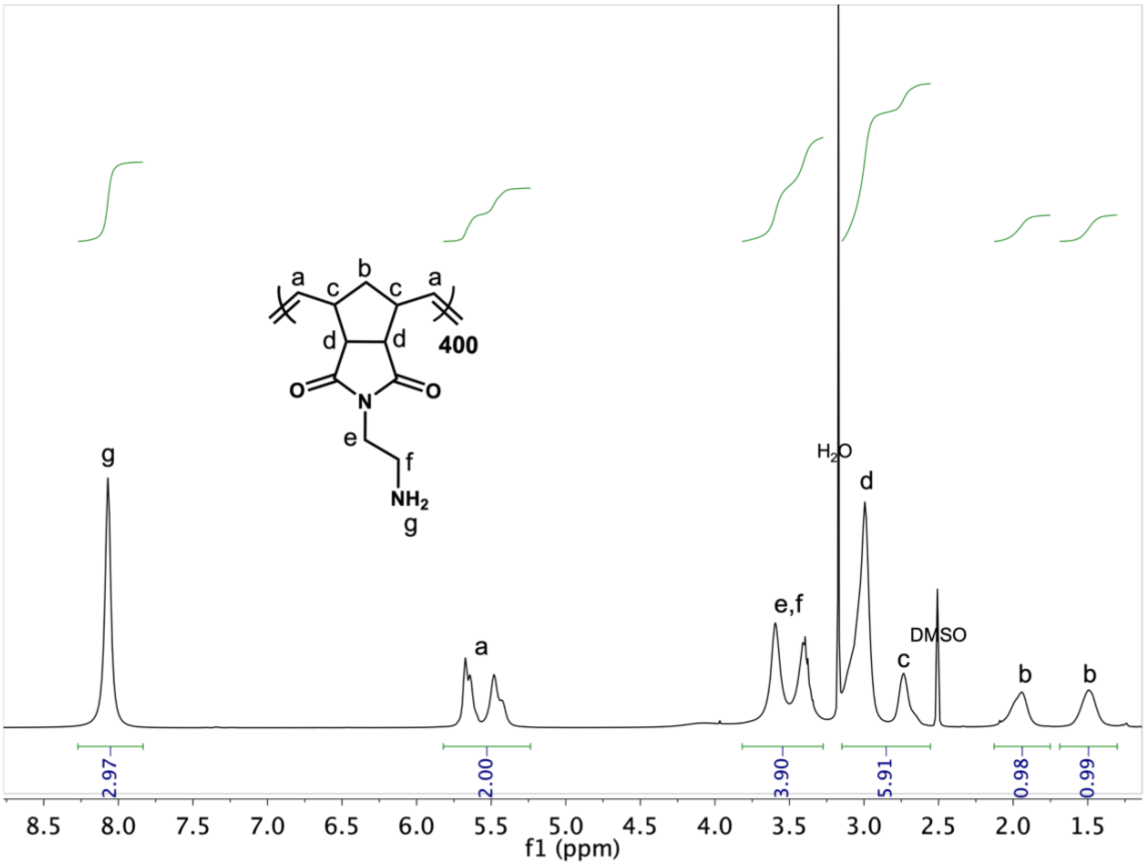

Figure S19. ^{1}H-NMR spectrum of P(NB-NH_2)$_{400}$ homopolymer in DMSO-d_6.

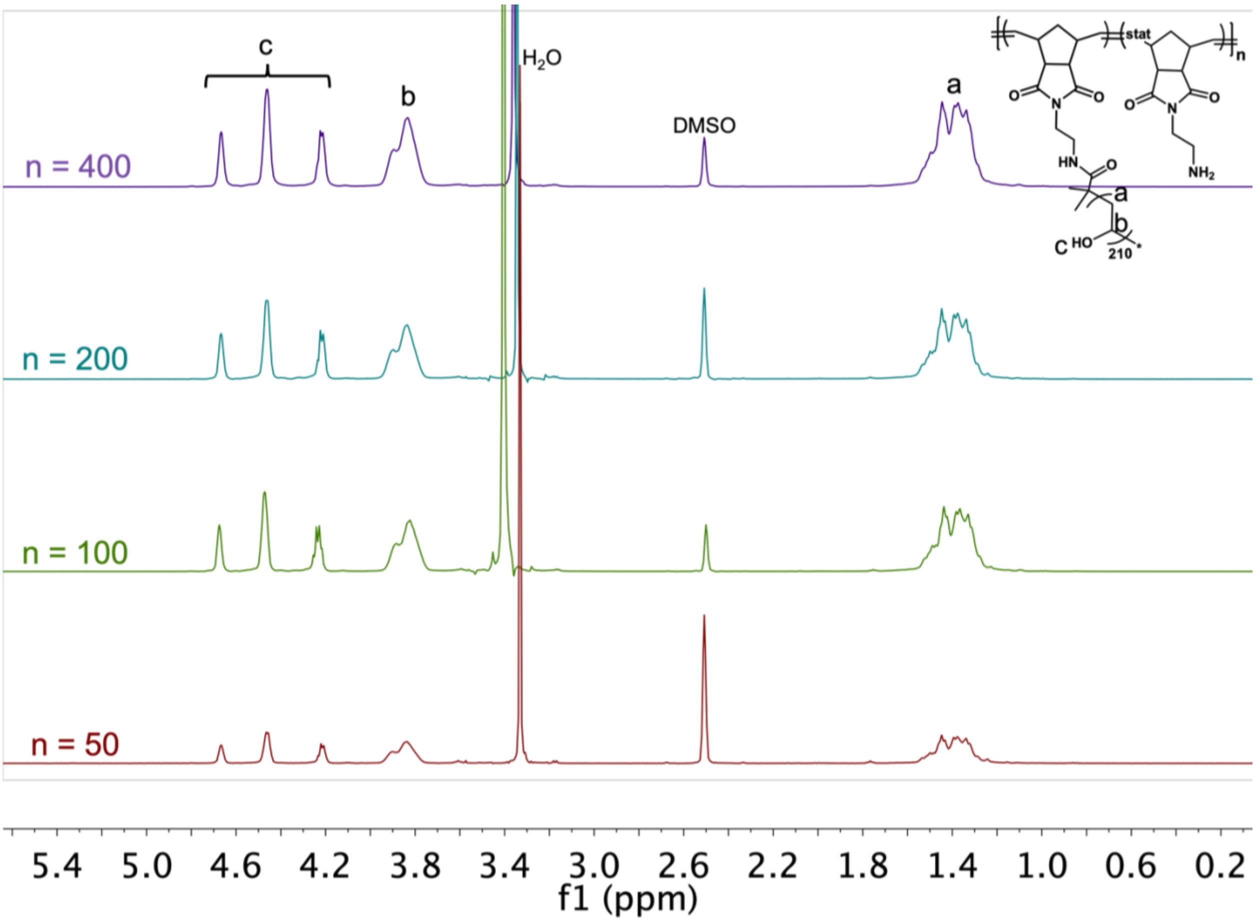

Figure S20. ^{1}H-NMR spectra of P((P(NB-NH)-*g*-PVA$_{210}$)-*stat*-P(NB-NH$_2$))$_n$ (n = 50, 100, 200, 400) graft copolymers recorded in DMSO-*d*$_6$.

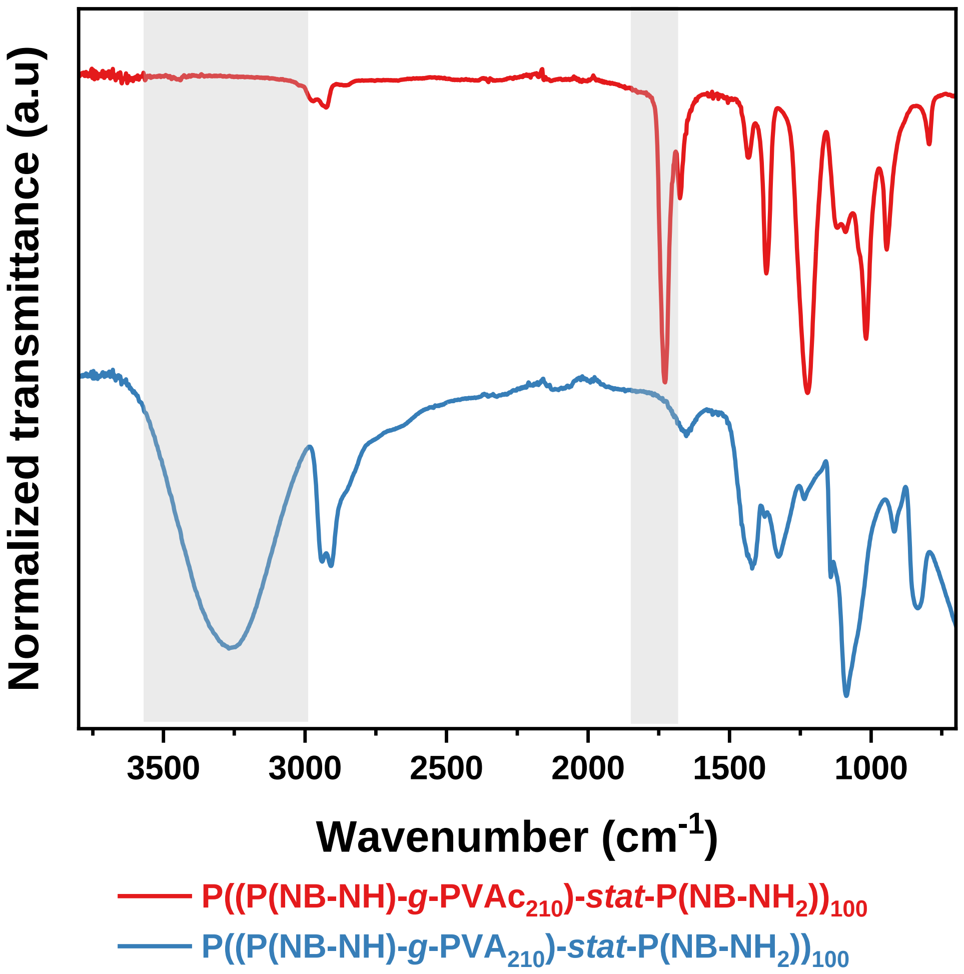

Figure S21. FTIR spectra recorded for P((P(NB-NH)-*g*-PVAc$_{210}$)-*stat*-P(NB-NH$_2$))$_{100}$ (red) and P((P(NB-NH)-*g*-PVA$_{210}$)-*stat*-P(NB-NH$_2$))$_{100}$ (blue) graft copolymers showing quantitative hydrolysis of the acetate esters.

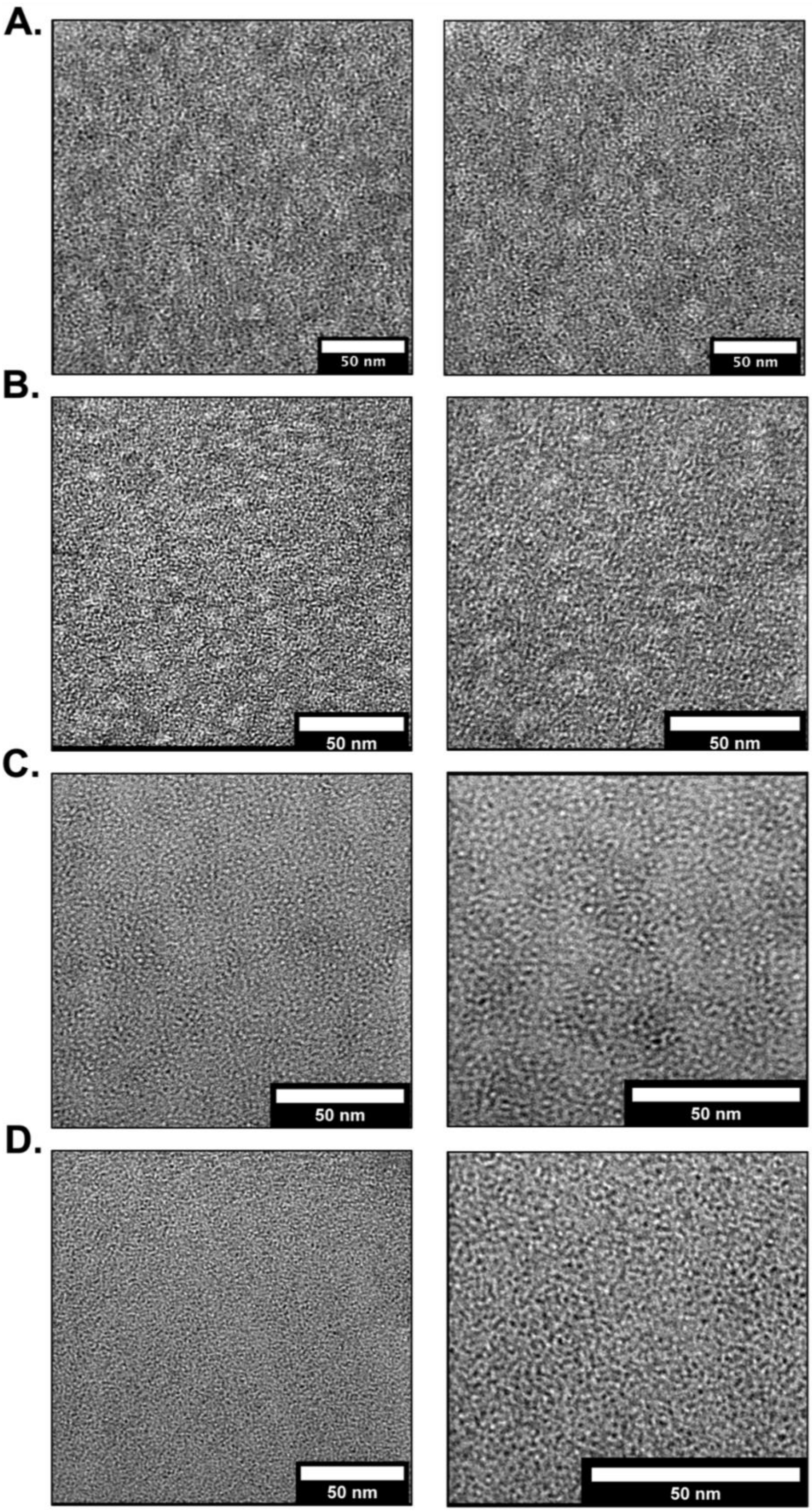

Figure S22. Representative dry-state TEM images obtained from a 5 mg.mL^{-1} solution of P((P(NB-NH)-*g*-PVA$_{210}$)-*stat*-P(NB-NH$_2$))$_n$ (n = 50 (A), 100 (B), 200 (C), 400 (D)) graft copolymers in water and stained with 1 wt % aqueous uranyl acetate (UA) solution.

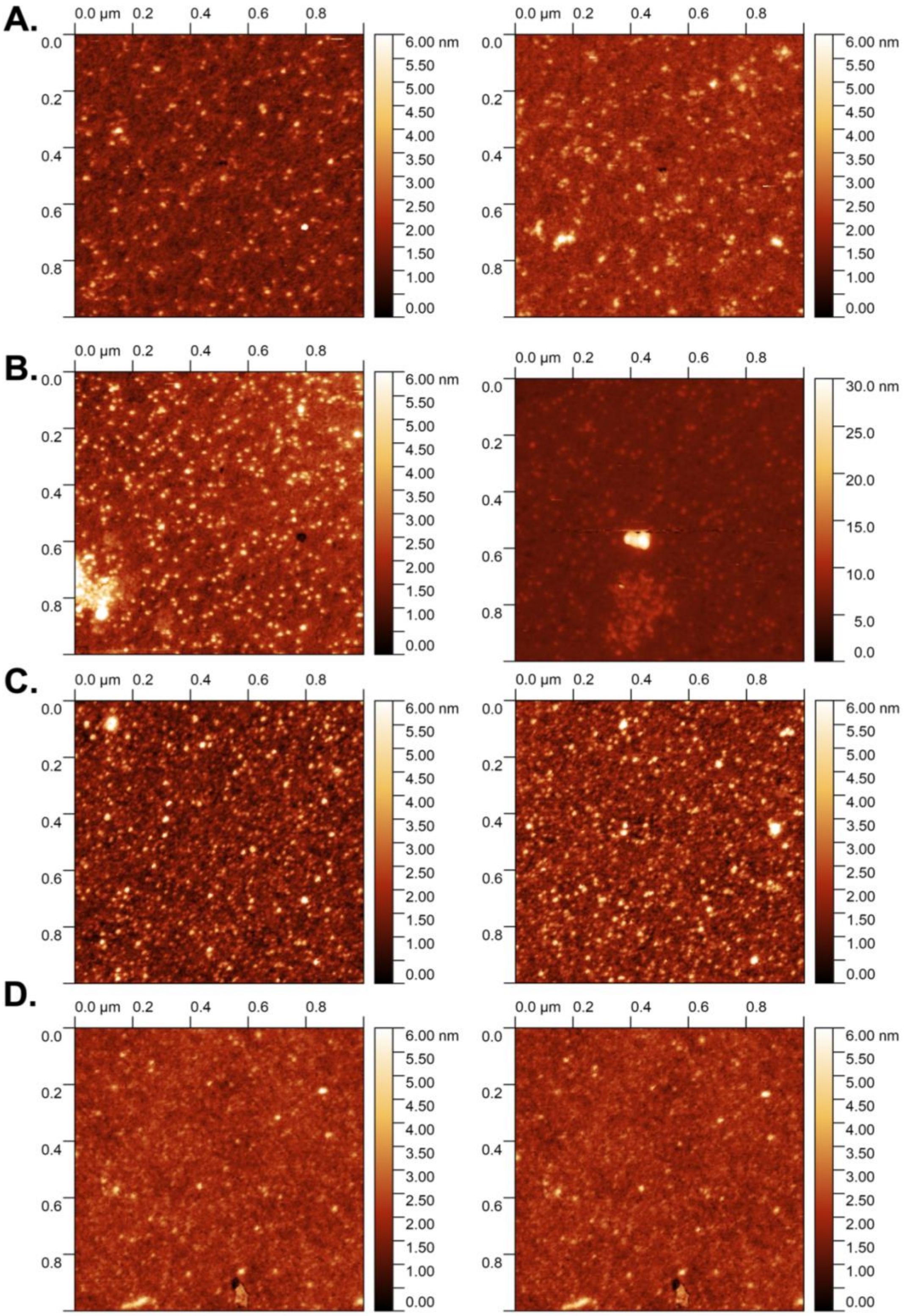

Figure S23. Representative AFM images obtained from a 5 mg.mL^{-1} solution of P((P(NB-NH)-*g*-PVA$_{210}$)-*stat*-P(NB-NH$_2$))$_n$ (n = 50 (A), 100 (B), 200 (C), 400 (D)) graft copolymers in water.

Supplementary Characterization Data for PNB$_n$-*g*-PVA$_{208}$ Bottlebrush Polymers

Table S3. Molecular characteristics of PNB$_n$-*g*-PVAc$_{208}$ (n = 20, 30, 40) bottlebrush polymers prepared *via* ROMP using different initial [NB-PVAc$_{208}$]/[G3] ratios, as determined by ^{1}H-NMR spectroscopy and SEC analysis

[NB-PVAc$_{208}$]/[G3]	% Conv.[a]	$M_{n,\ theo.}$ (kDa)[b]	$M_{n,\ SEC}$ (kDa)[c]	$Đ_{M,\ SEC}$[c]
20	>99	358.0	162.6	2.11
30	>99	537.0	241.1	2.41
40	>99	716.0	340.3	2.39

[a] Monomer conversion calculated from ^{1}H-NMR spectroscopy in $CDCl_3$. [b] Calculated from conversion. [c] M_n and $Đ_M$ values calculated from PMMA standards using DMF + 0.1% LiBr as the eluent.

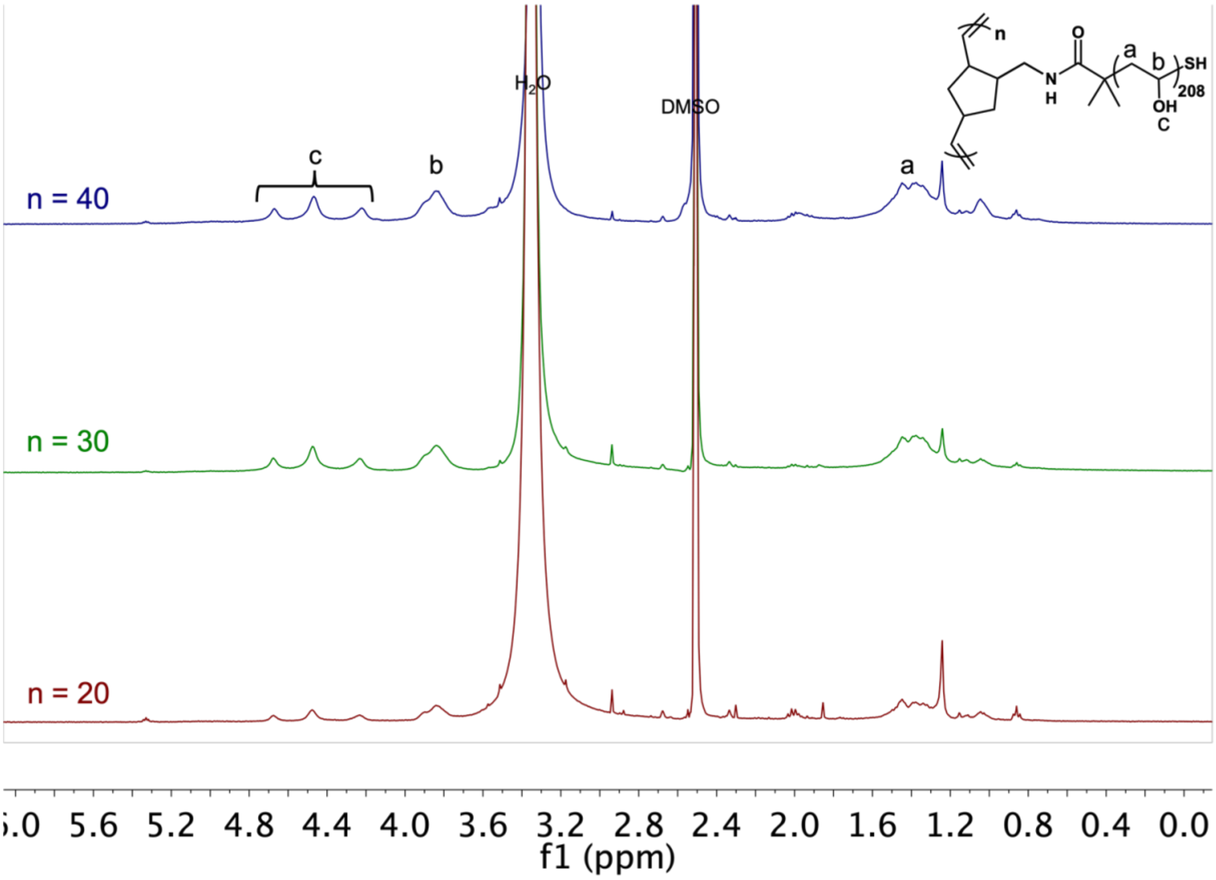

Figure S24. ^{1}H-NMR spectra of PNB$_n$-*g*-PVA$_{208}$ (n = 20, 30, 40) bottlebrush polymers recorded in DMSO-d_6.

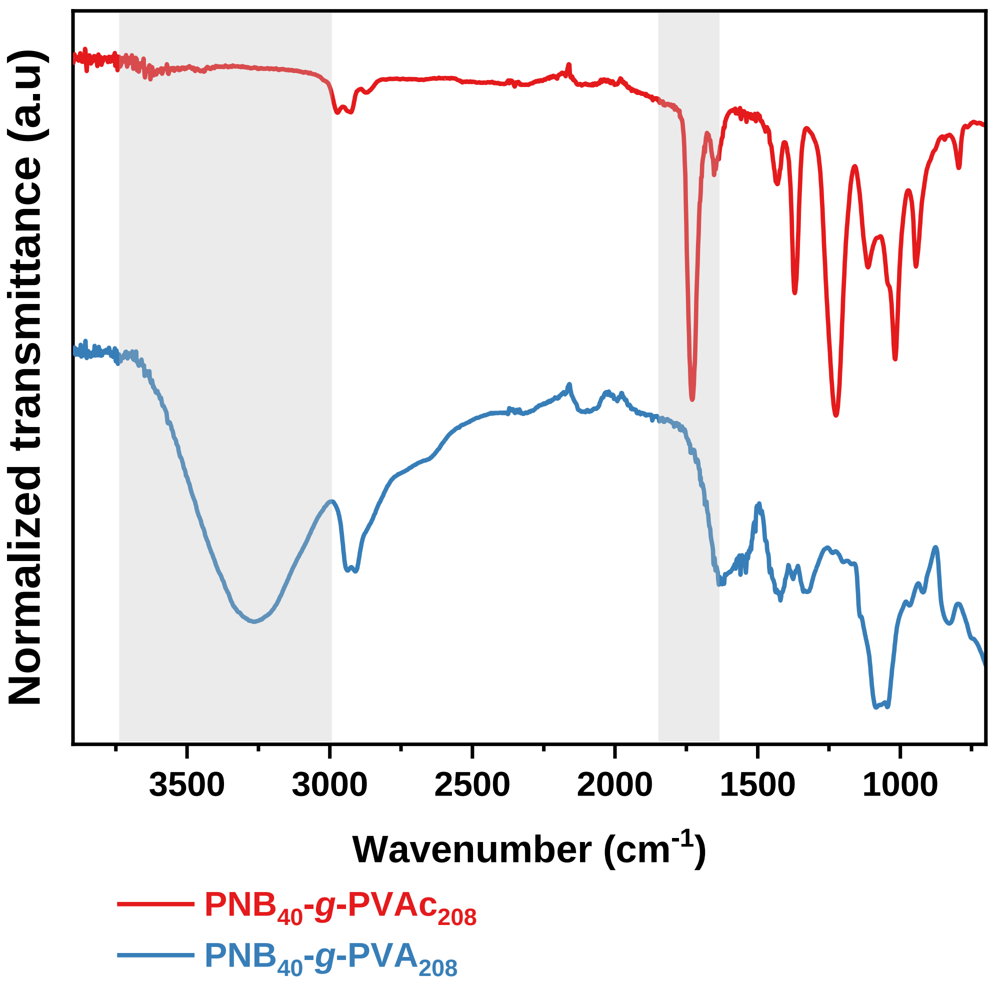

Figure S25. FTIR spectra recorded for PNB_{40}-*g*-$PVAc_{208}$ (red) and PNB_{40}-*g*-PVA_{208} (blue) bottlebrush polymers showing quantitative hydrolysis of the acetate esters.

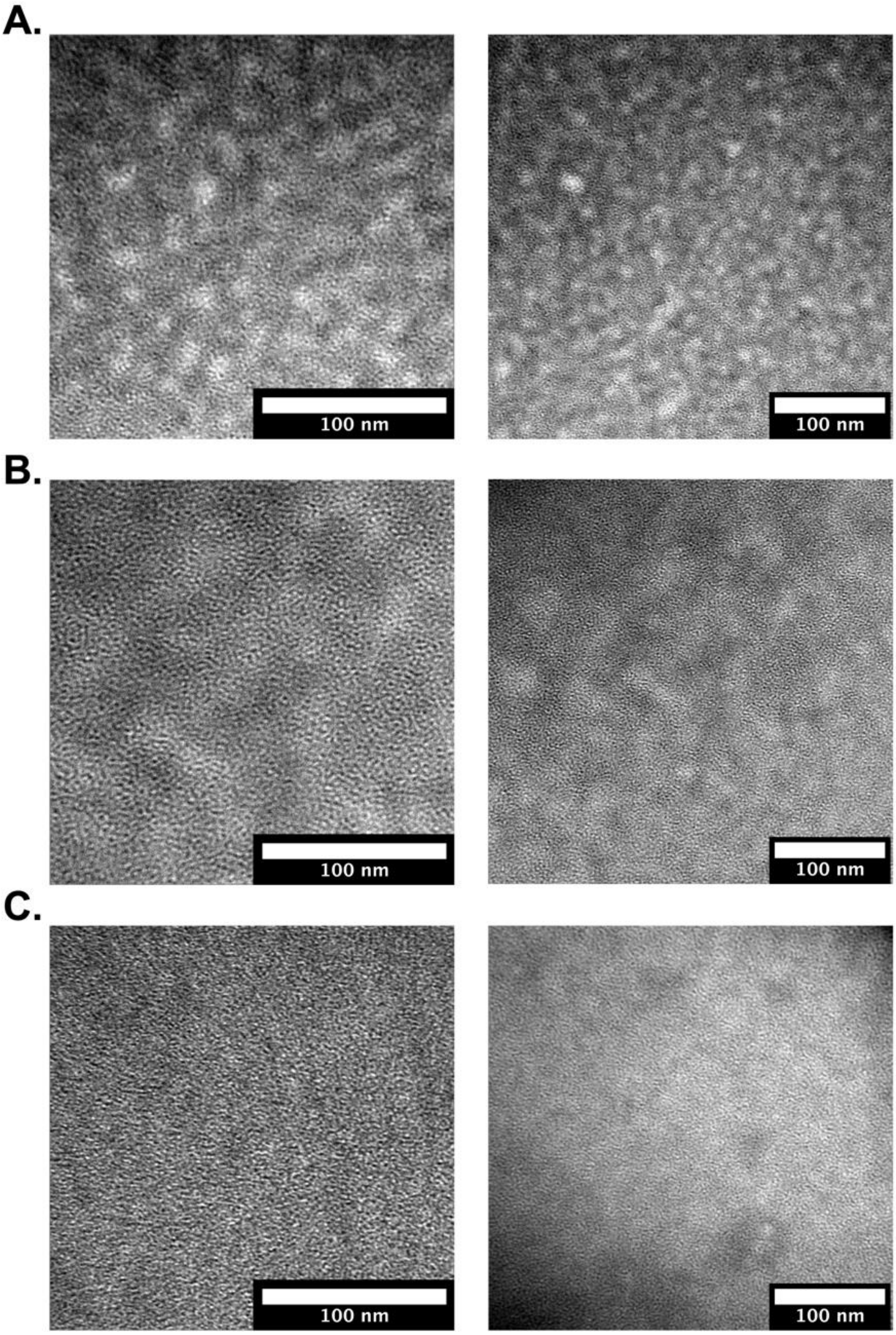

Figure S26. Representative dry-state TEM images obtained from a 5 mg.mL^{-1} solution of PNB$_n$-*g*-PVA$_{208}$ (n = 20 (A), 30 (B), 40 (C)) bottlebrush polymers in water and stained with 1 wt % aqueous uranyl acetate (UA) solution.

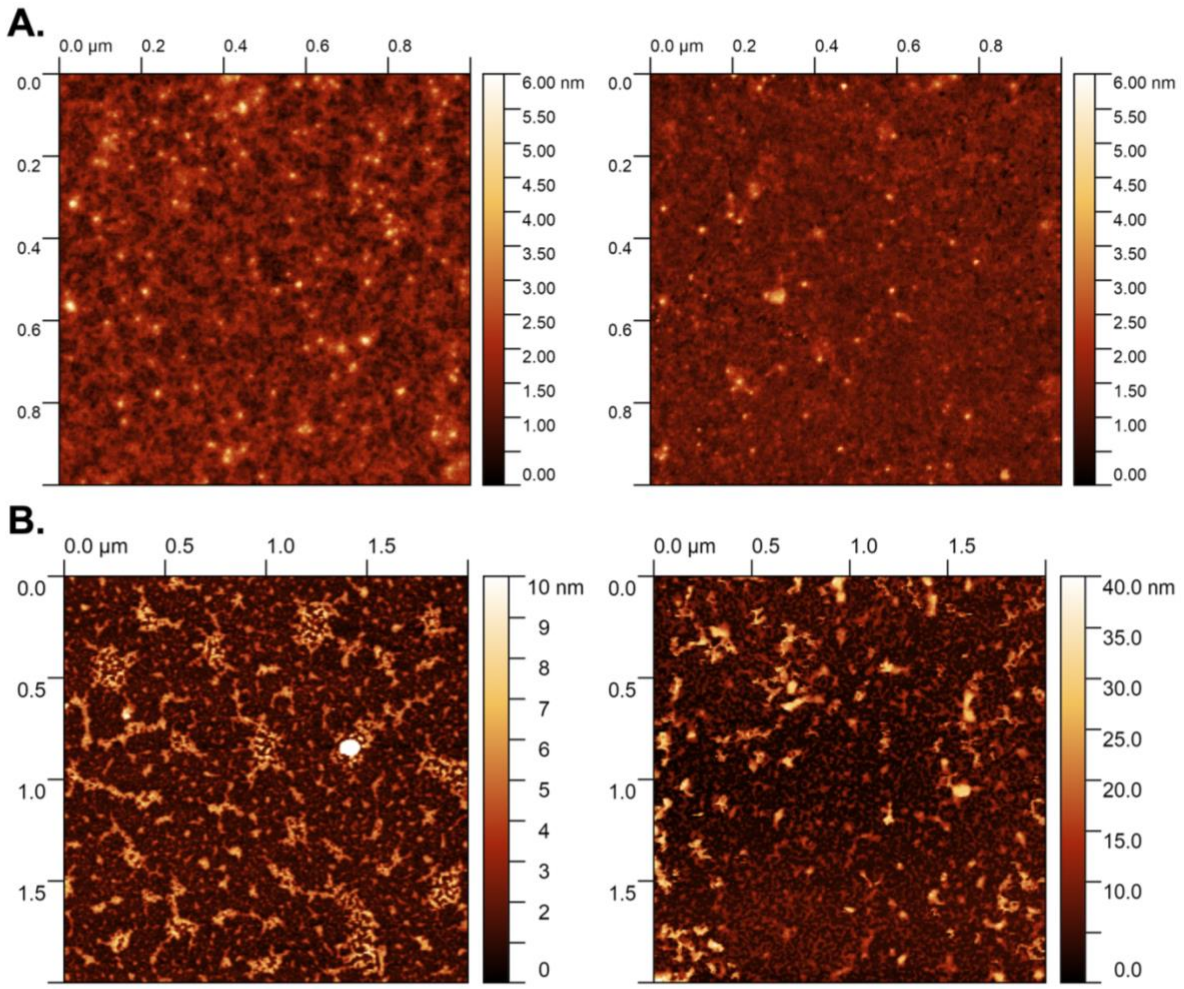

Figure S27. Representative AFM images obtained from a 5 mg.mL^{-1} solution of (A) PNB_{20}-*g*-PVA_{208} and (B) PNB_{40}-*g*-PVA_{208} bottlebrush polymers in water.

Supplementary Characterization Data for P((P(NB-NH)-*g*-PEG$_{227}$)-*stat*-P(NB-NH$_2$))$_{200}$ and P(PEGMA$_{20}$)$_{200}$ Control Samples

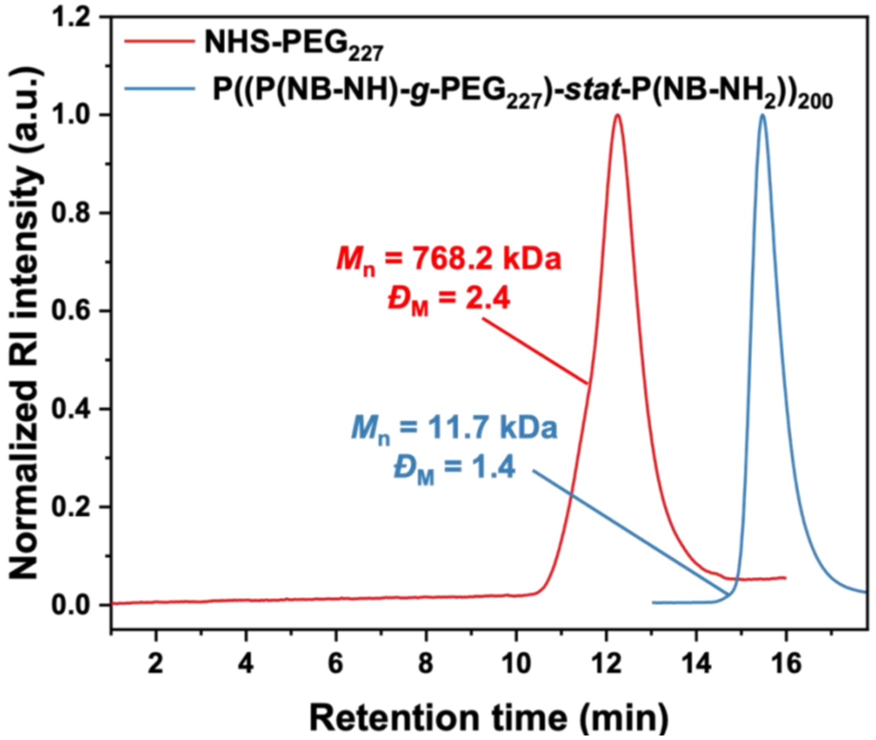

Figure S28. Normalized SEC RI molecular weight distributions for purified P((P(NB-NH)-*g*-PEG$_{227}$)-*stat*-P(NB-NH$_2$))$_{200}$ graft copolymer (blue trace) after dialysis and NHS-PEG$_{227}$ (red trace). M_n and $Đ_M$ values calculated from PMMA standards using DMF + 0.1% LiBr as the eluent.

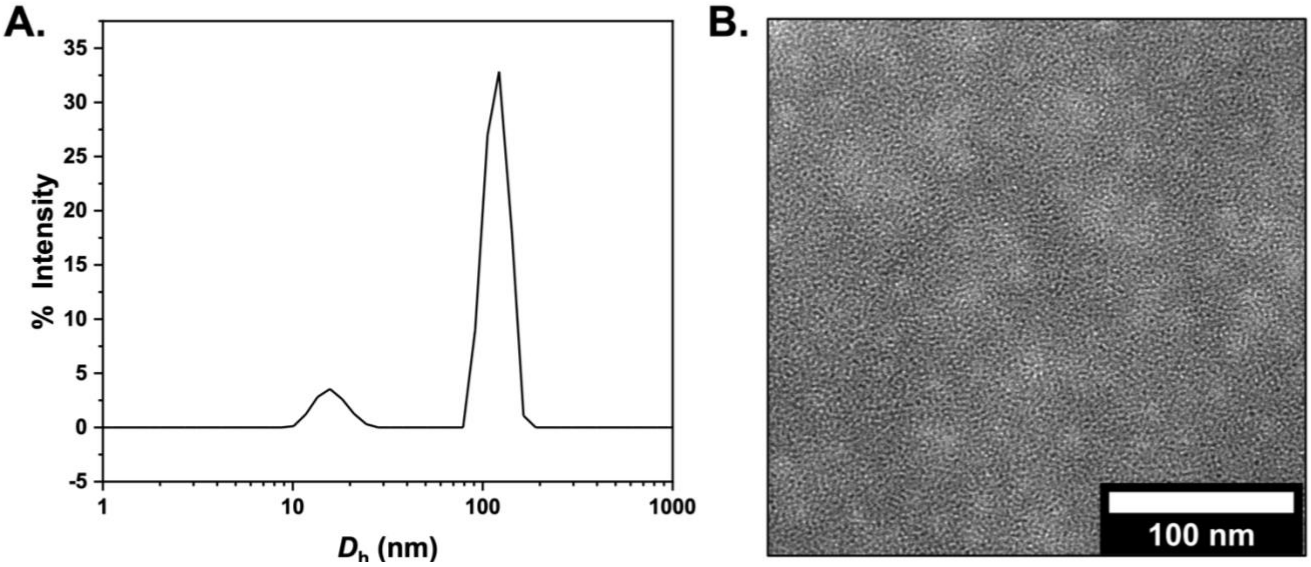

Figure S29. (A) Intensity-weighted size distribution obtained by DLS for P((P(NB-NH)-*g*-PEG$_{227}$)-*stat*-P(NB-NH$_2$))$_{200}$ graft copolymer in water. (B) Representative dry-state TEM image obtained from a 5 mg.mL^{-1} solution in water and stained with 1 wt % aqueous uranyl acetate (UA) solution.

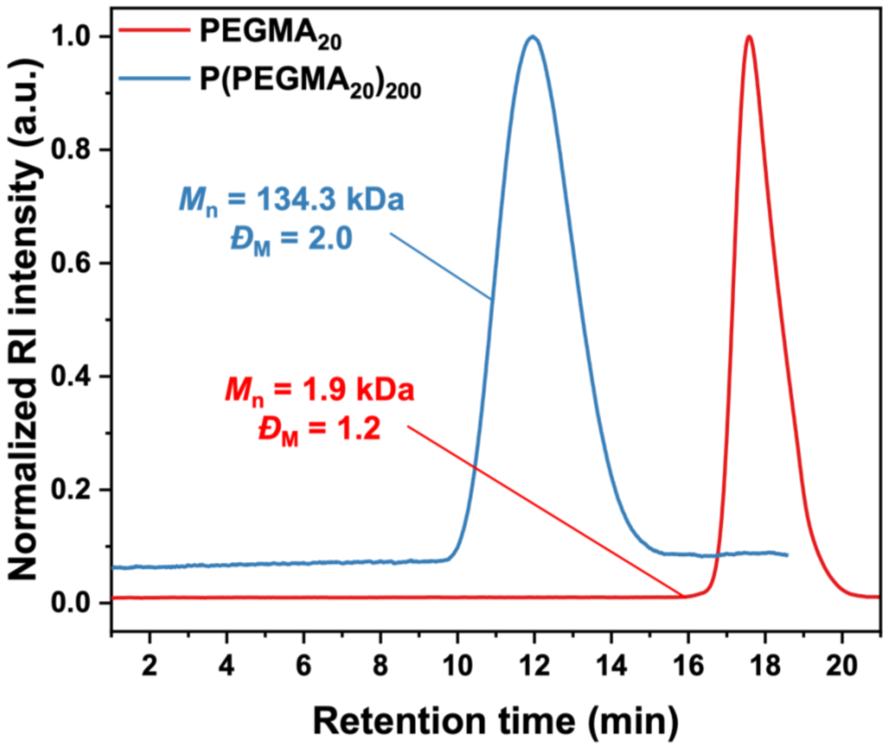

Figure S30. Normalized SEC RI molecular weight distributions for $P(PEGMA_{20})_{200}$ (blue trace) and $PEGMA_{20}$ (red trace). M_n and $Đ_M$ values calculated from PMMA standards using DMF + 5mM NH_4BF_4 as the eluent.

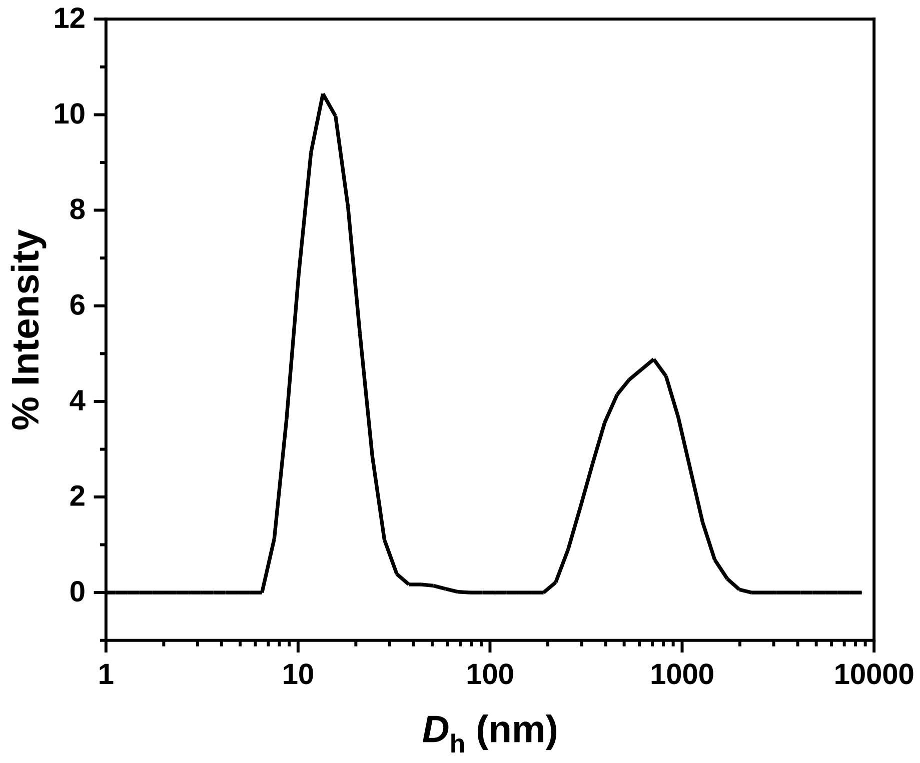

Figure S31. Intensity-weighted size distribution obtained by DLS for $P(PEGMA_{20})_{200}$ in water.

Supplementary Ice Shaping/Nucleation Data

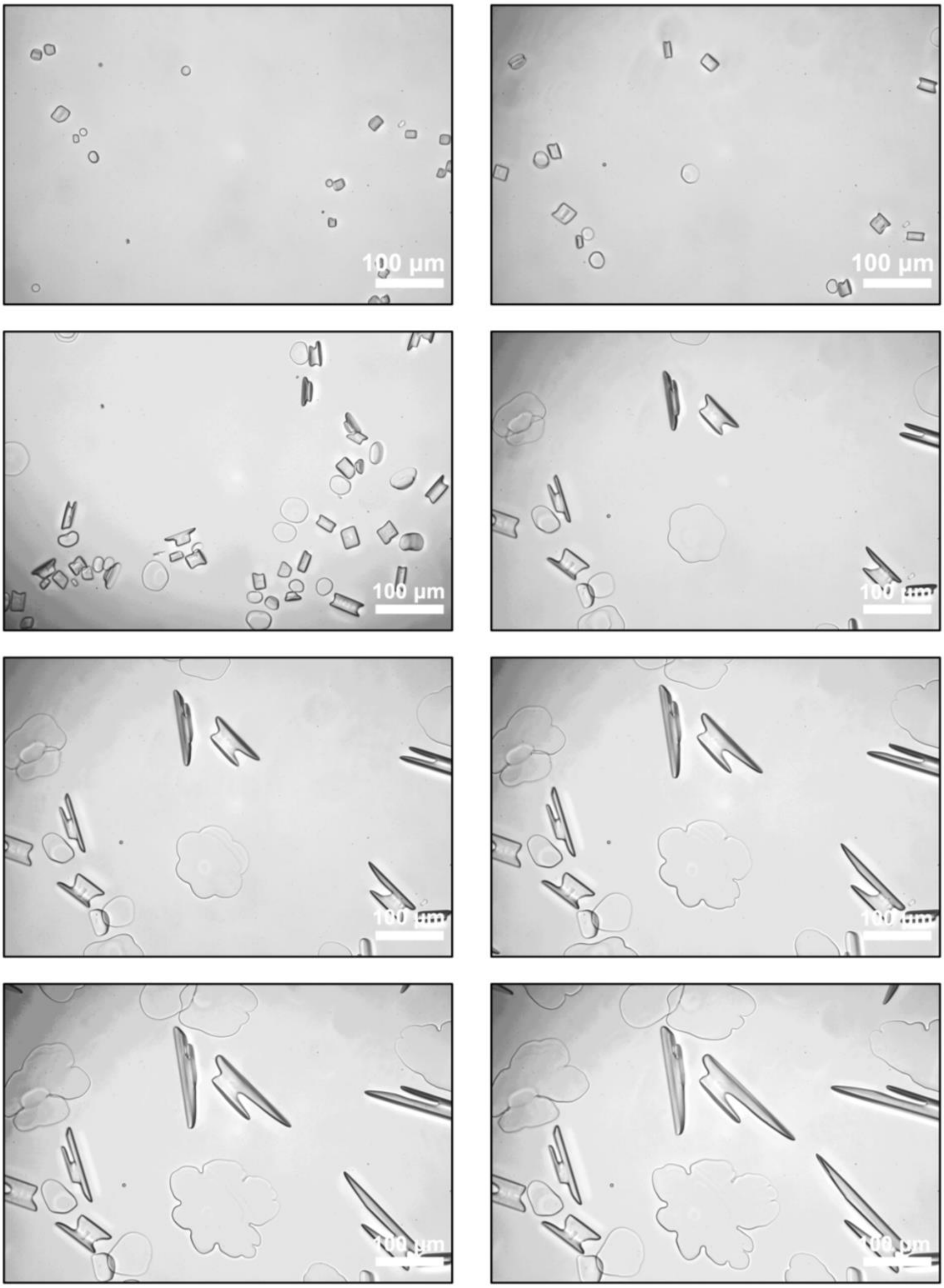

Figure S32. Modified sucrose "sandwich" ice shaping with no additive. Needle-shaped crystals are flat disks lying on their side rather than floating and their round basal plane uppermost.

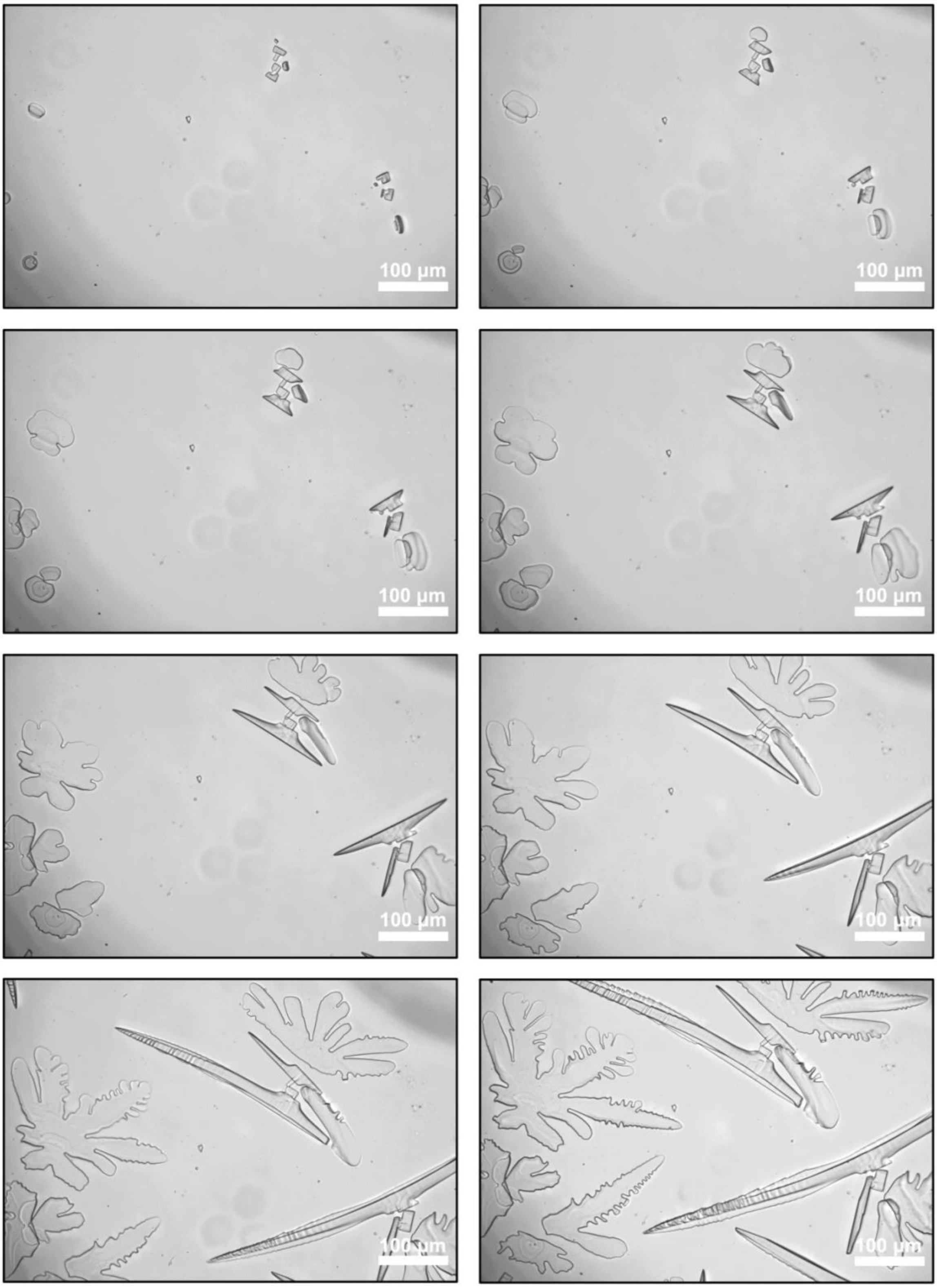

Figure S33. Modified sucrose "sandwich" ice shaping for 1 mg.mL^{-1} of PVA_{210} homopolymer. Needle-shaped crystals are flat disks lying on their side rather than floating and their round basal plane uppermost.

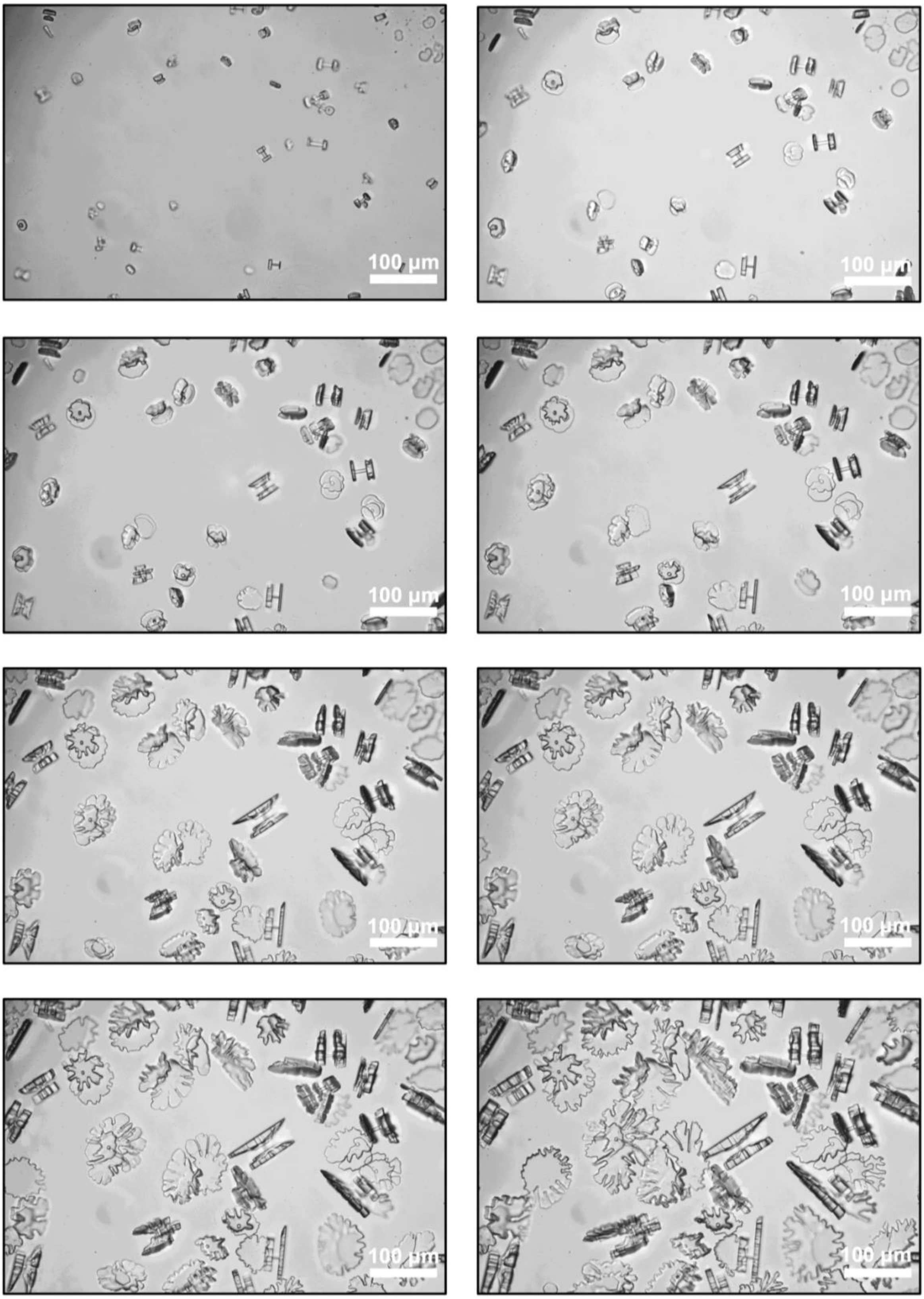

Figure S34. Modified sucrose "sandwich" ice shaping for 1 mg.mL^{-1} of P((P(NB-NH)-*g*-PVA$_{210}$)-*stat*-P(NB-NH$_2$))$_{400}$ graft copolymer. Needle-shaped crystals are flat disks lying on their side rather than floating and their round basal plane uppermost.

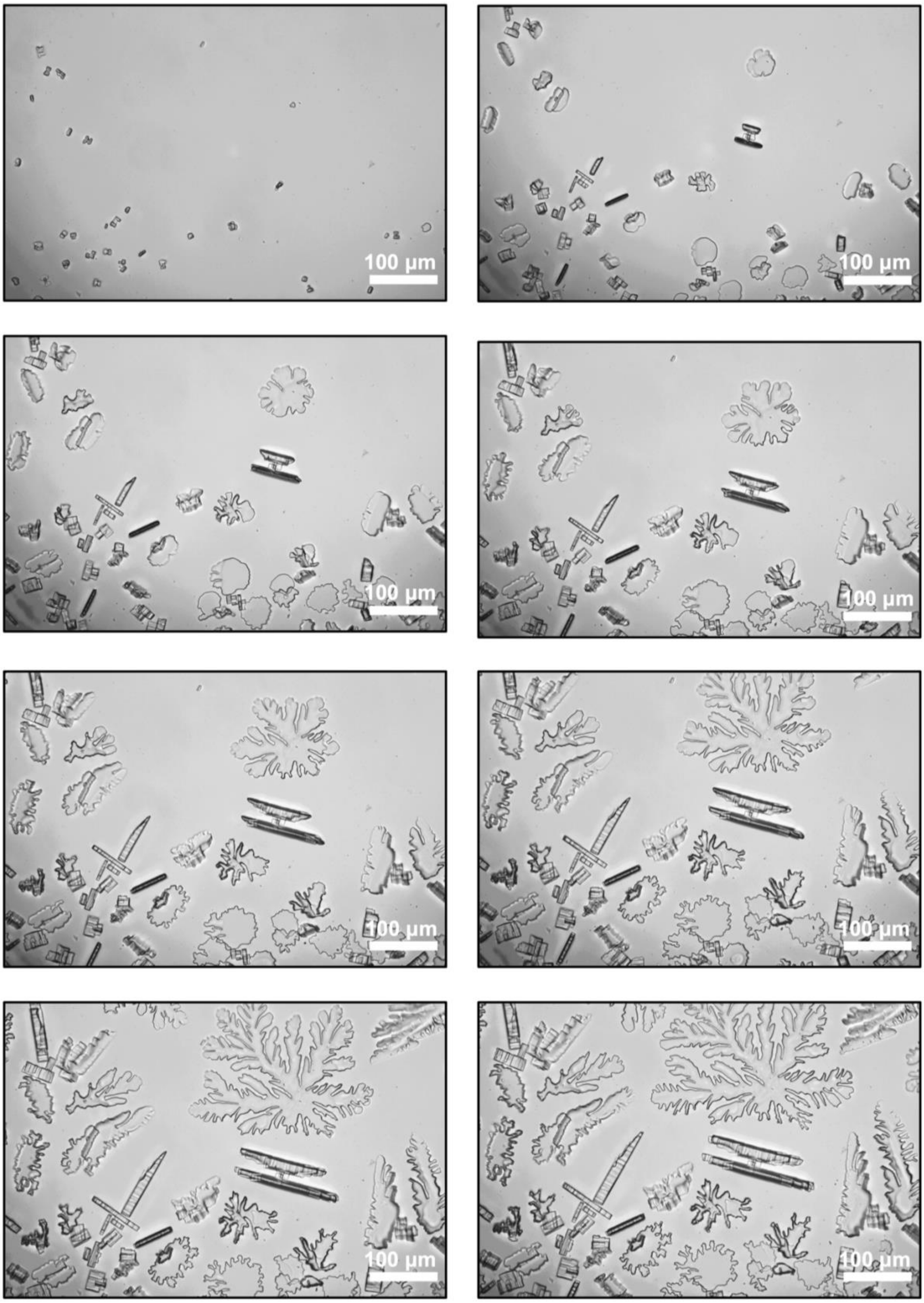

Figure S35. Modified sucrose "sandwich" ice shaping for 1 mg.mL^{-1} of PNB$_{40}$-*g*-PVA$_{208}$ bottlebrush polymer. Needle-shaped crystals are flat disks lying on their side rather than floating and their round basal plane uppermost.

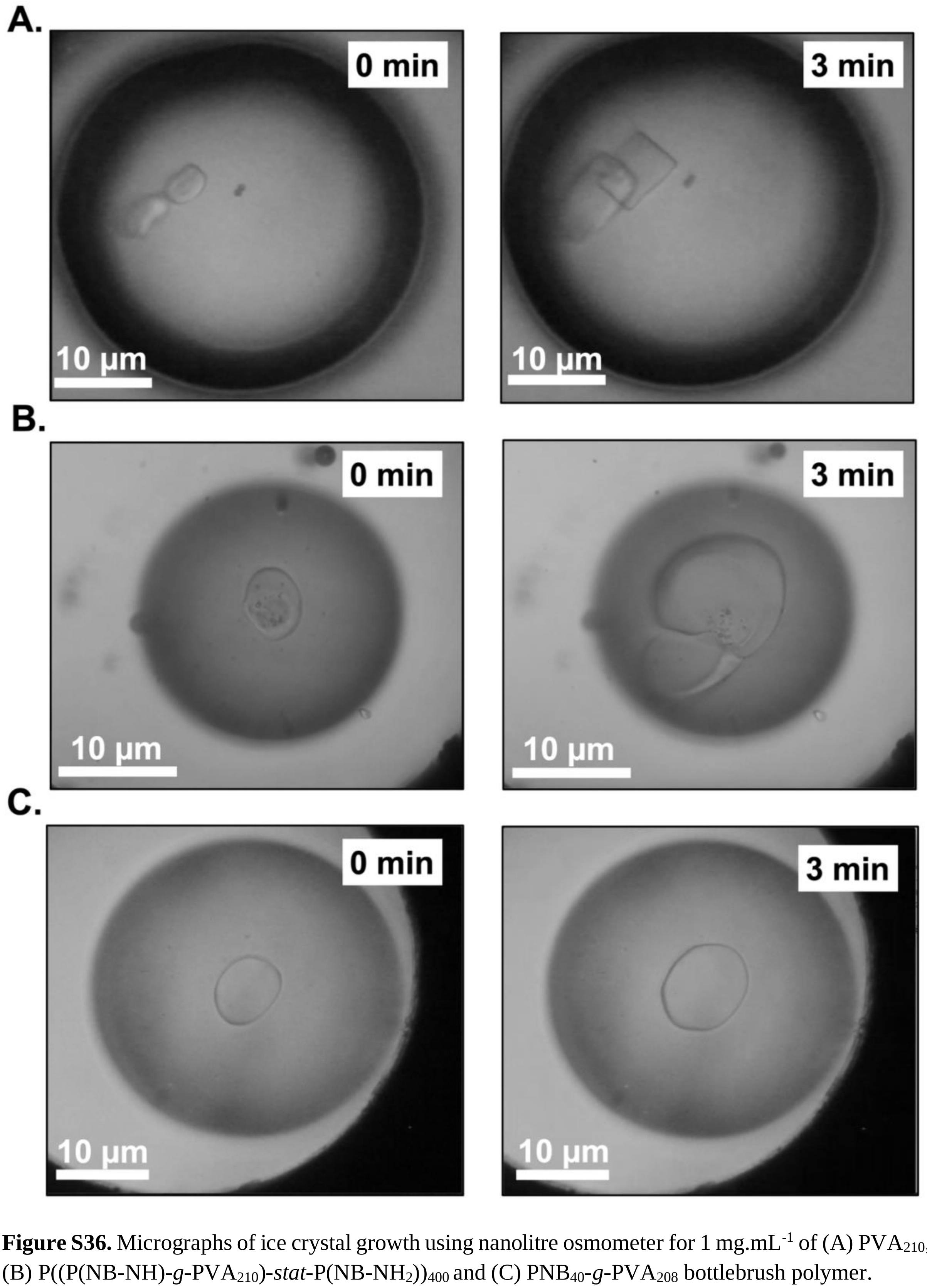

Figure S36. Micrographs of ice crystal growth using nanolitre osmometer for 1 mg.mL^{-1} of (A) PVA_{210}, (B) P((P(NB-NH)-*g*-PVA_{210})-*stat*-P(NB-NH_2))$_{400}$ and (C) PNB_{40}-*g*-PVA_{208} bottlebrush polymer.

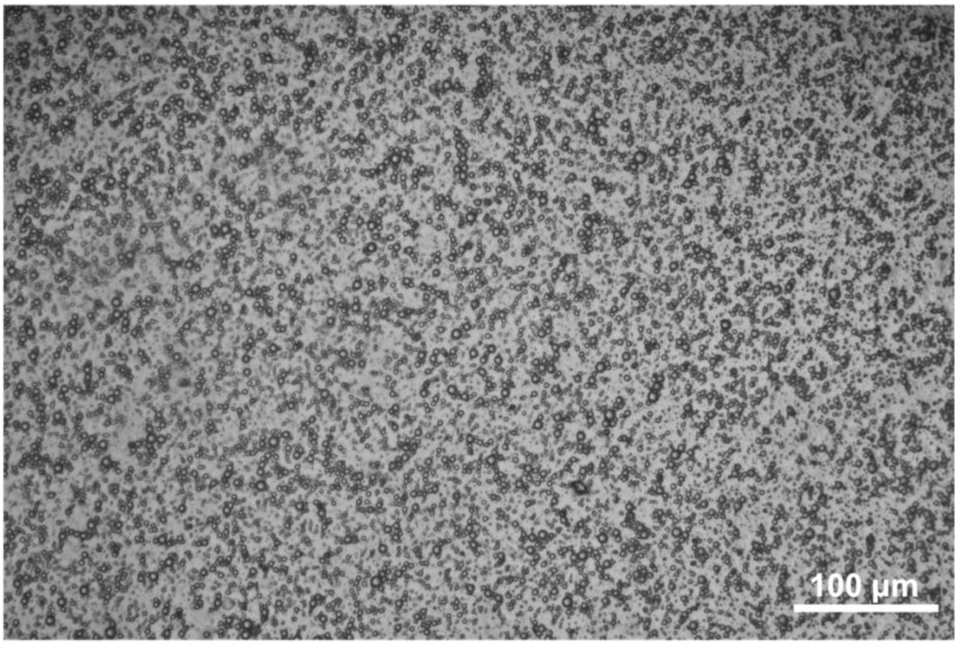

Figure S37. Representative optical microscopy images of initial emulsion for DSC.

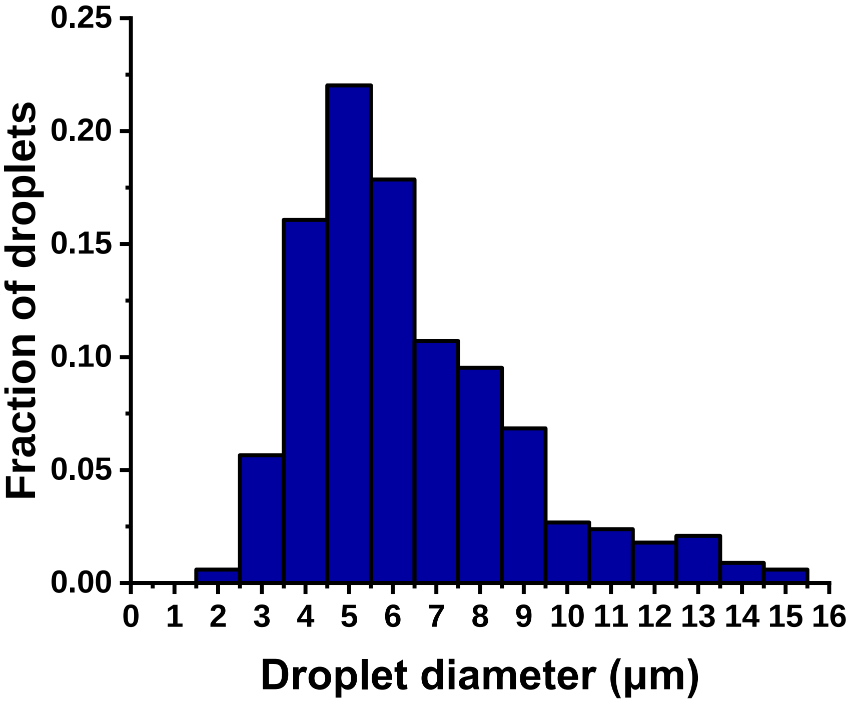

Figure S38. Droplet size distribution used for DSC analysis.

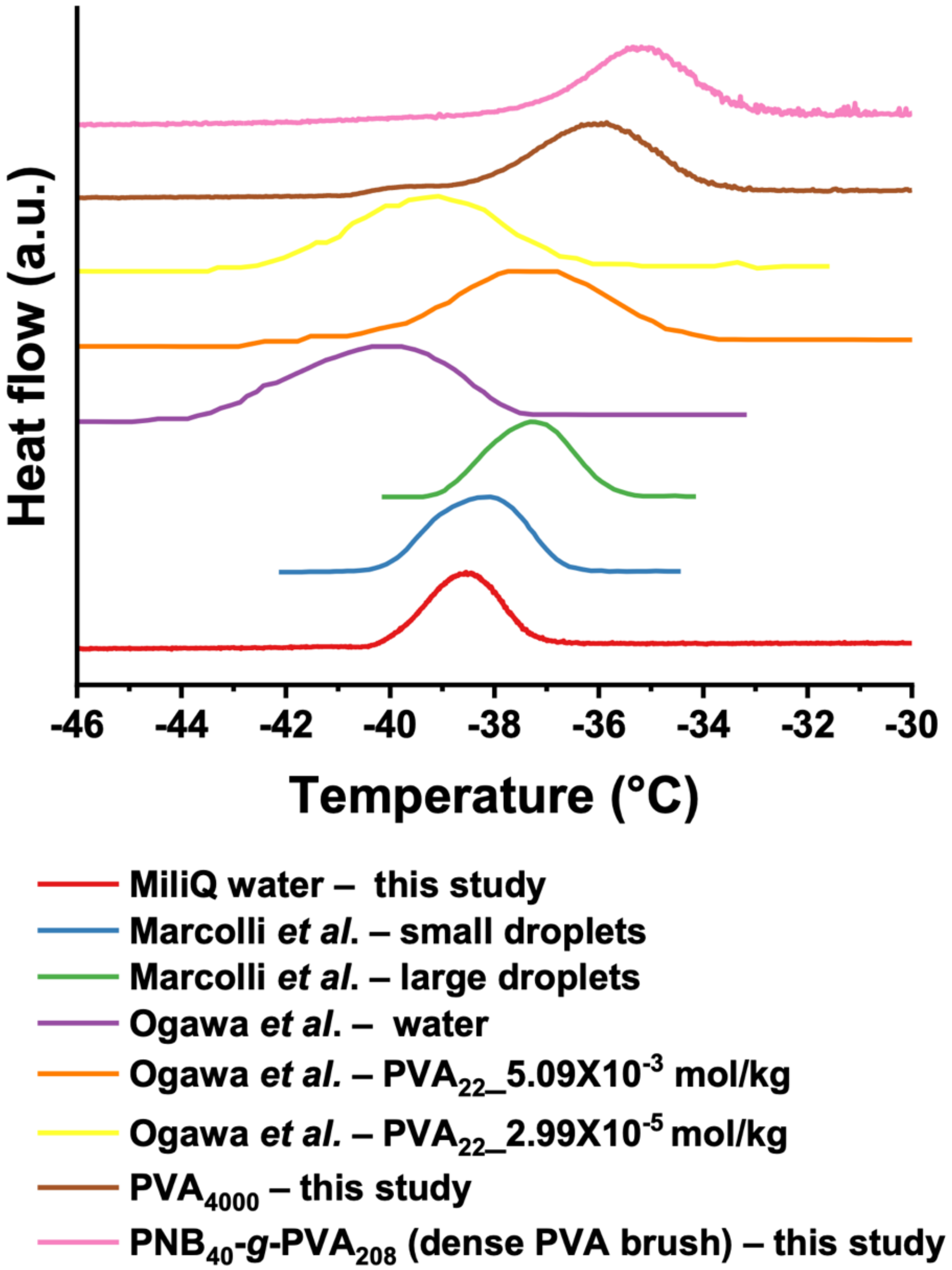

Figure S39. Comparison of ice nucleation DSC curves produced in this study and in literature studies.

References

[1] M. M. Tomczak, C. B. Marshall, J. A. Gilbert, P. L. Davies, *Biochem. Biophys. Res. Commun.* **2003**, *311*, 1041–1046.

[2] I. AU - Braslavsky, R. AU - Drori, *JoVE* **2013**, e4189.

[3] G. Bai, D. Gao, J. Wang, *Carbon N. Y.* **2017**, *124*, 415–421.

[4] T. F. Whale, B. J. Murray, D. O'Sullivan, T. W. Wilson, N. S. Umo, K. J. Baustian, J. D. Atkinson, D. A. Workneh, G. J. Morris, *Atmos. Meas. Tech.* **2015**, *8*, 2437–2447.

[5] G. Vali, *Atmos. Meas. Tech.* **2019**, *12*, 1219–1231.

[6] M. Polen, T. Brubaker, J. Somers, R. C. Sullivan, *Atmos. Meas. Tech.* **2018**, *11*, 5315–5334.

[7] C. Marcolli, S. Gedamke, T. Peter, B. Zobrist, *Atmos. Chem. Phys.* **2007**, *7*, 5081–5091.

[8] A. Kumar, C. Marcolli, B. Luo, T. Peter, *Atmos. Chem. Phys.* **2018**, *18*, 7057–7079.

[9] M. P. Thompson, L. M. Randolph, C. R. James, A. N. Davalos, M. E. Hahn, N. C. Gianneschi, *Polym. Chem.* **2014**, *5*, 1954–1964.

[10] S. Varlas, R. Keogh, Y. Xie, S. L. Horswell, J. C. Foster, R. K. O'Reilly, *J. Am. Chem. Soc.* **2019**, *141*, 20234–20248.

[11] L. E. Strong, L. L. Kiessling, *J. Am. Chem. Soc.* **1999**, *121*, 6193–6196.

[12] J. K. Pontrello, M. J. Allen, E. S. Underbakke, L. L. Kiessling, *J. Am. Chem. Soc.* **2005**, *127*, 14536–14537.

[13] S. Varlas, J. C. Foster, L. A. Arkinstall, J. R. Jones, R. Keogh, R. T. Mathers, R. K. O'Reilly, *ACS Macro Lett.* **2019**, *8*, 466–472.

[14] E. M. Kolonko, J. K. Pontrello, S. L. Mangold, L. L. Kiessling, *J. Am. Chem. Soc.* **2009**, *131*, 7327–7333.
